# Synthesis and Evaluation of Europium Complexes that Switch on Luminescence in Lysosomes of Living Cells

**DOI:** 10.1002/chem.202003992

**Published:** 2020-12-15

**Authors:** Matthieu Starck, Jack D. Fradgley, Robert Pal, Jurriaan M. Zwier, Laurent Lamarque, David Parker

**Affiliations:** ^1^ Department of Chemistry Durham University South Road Durham DH1 3LE UK; ^2^ Research and Development Cisbio Bioassays BP 84175 30200 Codolet France

**Keywords:** Cells, Europium, Luminescence, Lysosomes, pH

## Abstract

A set of four luminescent Eu^III^ complexes bearing an extended aryl‐alkynylpyridine chromophore has been studied, showing very different pH‐dependent behaviour in their absorption and emission spectral response. For two complexes with p*K*_a_ values of 6.45 and 6.20 in protein‐containing solution, the emission lifetime increases very significantly following protonation. By varying the gate time during signal acquisition, the ‘switch‐on’ intensity ratio could be optimised, and enhancement factors of between 250 to 1330 were measured between pH 8 and 4. The best‐behaved probe showed no significant emission dependence on the concentration of endogenous cations, reductants, and serum albumin. It was examined in live‐cell imaging studies to monitor time‐dependent lysosomal acidification, for which the increase in observed image brightness due to acidification was a factor of 50 in NIH‐3T3 cells.

## Graphical abstract

**Watching lysosomes age** with a ‘switch‐on’ optical sensor is shown to be possible by using four europium complexes with extended aryl‐alkylnyl pyridine antennae that show very different pH‐dependent behaviour. A ‘switch‐on’ of europium luminescence occurs at low pH, allowing confocal microscopy to reveal increases in image brightness of ageing lysosomes in live‐cell imaging studies.

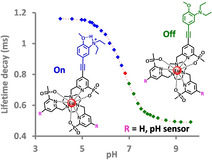

## Introduction

We introduce a series of four Eu^III^ complexes whose luminescence emission is switched on by over two orders of magnitude during acidification, following excitation in the range 330 to 370 nm. Such complexes have utility in monitoring acidification in living cells.

Receptor mediated endocytosis is a ubiquitous feature of uptake into cells, following surface receptor membrane binding.^[1]^ It leads to the internalisation of part of the cell surface receptors together with the bound molecular species. Thus, in a typical clathrin‐mediated cell uptake process^[2]^ with a G‐protein coupled receptor (GPCR), binding of an agonist at the receptor site may be followed by endosomal formation. The endosome may either be degraded, or its contents recycled, releasing the receptor to return to the plasma membrane. Much larger particles and species may be internalised by phagocytosis, after the cell surface membrane is ruffled, leading to the budding‐off of a phagosome within the cell. For species of intermediate molecular weight (MW) and for certain other low MW molecules and a large family of lanthanide coordination complexes, macropinocytosis may occur following invagination of the plasma membrane after surface receptor binding.^[3]^ The internalised macropinosome is considered to be more leaky than endosomes or vesicles, and discharges its contents more readily.

A common feature of the ageing process of endosomes and phagosomes is that their pH tends to fall with time over the cell cycle period, and endosomes may finally evolve into lysosomes. Thus, whilst cytosolic pH is around 7.2, endosomal pH ranges from 6.5 to 5.5 and the lowest pH is to be found in mature lysosomes and is typically around 4.5. The processes of internalisation and endosomal uptake can therefore be followed in time if the species being internalised (receptor or substrate) is for instance labelled with a luminescent pH sensitive dye whose emission intensity or lifetime varies with pH. Ideally, the observed change should be as large as possible;^[4]^ in the limit, this is akin to the idea of a ‘light switch’, although this term has sometimes been misused in the literature, and occasionally rather modest emission intensity variations have been unduly celebrated as ‘switch‐on’ sensors. Ratiometric systems of course offer greater scope. For this work, we set ourselves a target of a 100 % change in lifetime over the pH range 8–4, and a change in the long‐lived emission intensity of two orders of magnitude. Such desirable properties require careful consideration of the mechanism of the process leading to emission quenching at higher pH. In addition, the selection of the pH range determines the p*K*_a_ of the emissive species undergoing protonation. Our initial work focused on examining systems with p*K*_a_ around 5.5–6.

The majority of work reported has involved an adaptation of the molecular structure of common fluorophores, such as rhodamine, BODIPY and various cyanine dyes, in order to tune the p*K*_a_ value and permit conjugation. 
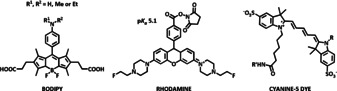
 Several of these examples are now commercially available. Thus, the *N*,*N′*‐fluoroethylrhodamine derivative (RH‐PEF) has been used to monitor antibody internalisation,^[5]^ the core structure has a p*K*_a_ around 5.1 and the intensity increase is reported to be a factor of 58 between pH 7.4 and 5.0. Related studies have used fluorescent cyanine dye labels and an assay was developed to track the internalisation of the HER‐2 antibody,^[6]^ while Grover et al.^[7]^ examined GPCR labelling with cyanine dyes, giving modest intensity enhancements of a factor of 5, between pH 5 and 8. Arguably the most promising approach has used a modified BODIPY core, and Nagano and colleagues^[8]^ reported switching factors of up to 300 using these systems. Here, the p*K*_a_ is easily tuned by variation of the aniline substituents R^1^ and R^2^: the p*K*_a_ increases from 3.8 to 6.0 in the sequence H<Me<Et, as lone pair conjugation is increasingly disfavoured and the conjugate acid is less strongly stabilised by solvation. Such systems were used to examine breast cancer tissue samples and permitted the internalisation of the antibody HER‐2 receptor complex to be followed by microscopy in mice, by labelling the immunotherapeutic agent Herceptin (trastuzumab) with a BODIPY dye.

These fluorescent systems offer neither significant lifetime modulation, nor a ratiometric response and have the inherent issues associated with autofluorescence. Others have therefore sought to use luminescent lanthanide labels, and benefit from the advantages (e.g., large Stokes shifts, long lifetimes), associated with time‐resolved spectroscopy or microscopy methods. Yuan and co‐workers,^[9]^ for example, has reported monitoring the change in the red/green Eu to Tb emission intensity ratio in a modified acyclic chelate based on a terpyridine ligand, leading to a system with a ratiometric ratio variation of a factor of 7. Papkovsky and colleagues^[10]^ described a rather weakly emissive Eu^III^ DTPA complex based on a carbostyril sensitiser, in which the lifetime and emission intensity at 614 nm changed by a factor of 7 between pH 7.5 and 6.5 (p*K*_a_ 6.5, *λ*_exc_ 370 nm), allowing extracellular acidification to be monitored for a series of high throughput assays. In another approach by Schäferling and co‐workers,^[10b]^ a Eu^III^ chelate was coupled to carboxynaphthofluorescein to create a ratiometric pH sensor, but this compound is unlikely to work in a cellular environment.

Fewer time‐resolved studies have examined pH change inside cells directly, where either confocal microscopy directly or spectral imaging can be employed, observing lifetime or emission intensity modulation. Using kinetically stable macrocyclic ligand systems based on triazacyclononane, McMahon et al.^[11]^ devised Eu^III^ complexes that permitted monitoring of the pH of the endoplasmic reticulum in live cells, based on emission lifetime variation; a 75 % change in the europium lifetime was observed over the pH range 6.5–7.5. Earlier, Smith et al.^[12]^ had described ratiometric methods that allow the real‐time monitoring of lysosomal pH, using confocal microscopy, based on analysis of the Eu/Tb emission intensity ratio, that varied from 1.3 at pH 4.6 to 2.9 at pH 6.5 (*λ*_exc_ 355 nm).

None of these lanthanide systems permits the conjugation of the emissive probe to a targeting vector or protein. Such an approach is straightforward to devise and simply requires a suitable linkage site to be introduced into the ligand structure. Accordingly, we set out to compare the pH response behaviour of a set of four luminescent Eu^III^ complexes, in which the site of protonation was either in partial conjugation with the sensitising chromophore, [Eu***L*** ^***3, 4***^], or where a methylene spacer insulates the amine site, [Eu***L*** ^***1, 2***^]. In the latter case, it was reasoned that acidification would lead to suppression of photo‐induced electron transfer, from the nitrogen lone pair to the chromophore excited state, enhancing the intensity of Eu emission by analogy with a plethora of published examples.[^4a^, ^13^] Such a system was not expected to show any change in the form of either emission or excitation spectra. With the amine group in conjugation with one of the aromatic rings, it was reasoned that the absorption spectrum would vary with pH and that significant Eu lifetime and intensity change was possible, as acidification would perturb the energy of the ligand centred and internal charge transfer transitions that exist in related examples of these ‘EuroTracker’ complexes.^[14]^ The nature of the substituents in the amine moiety was selected bearing in mind the p*K*_a_ values of simple anilines in aqueous media.

Thus, the series of Eu complexes [Eu***L*** ^***1–4***^] was designed and prepared and their photophysical behaviour examined, together with preliminary studies of their localisation and time‐dependent behaviour in living cells, in the expectation that the best examples could be taken forward as ‘core’ complexes, from which analogues would be designed to allow conjugation, for future internalisation studies. 
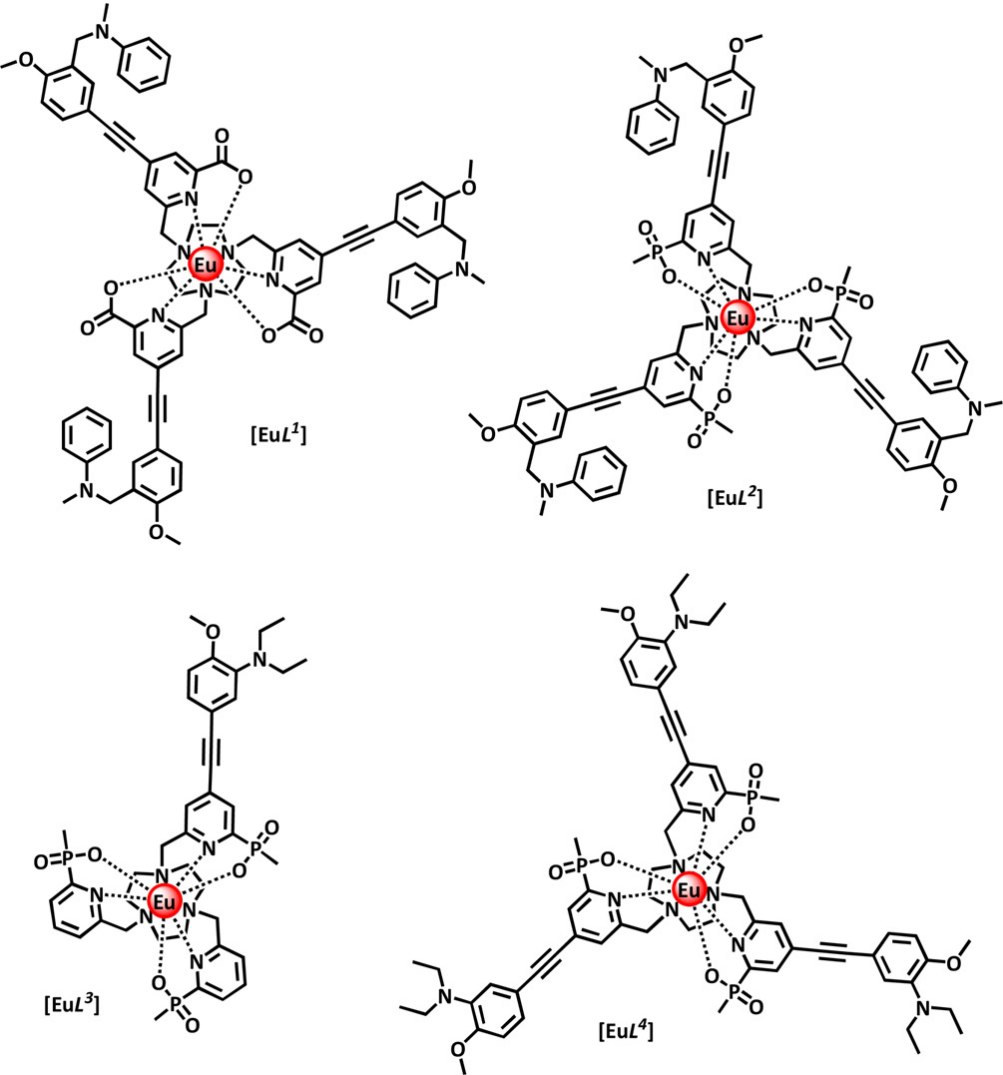


## Results and Discussion

### Synthesis of complexes

**Scheme 1:**
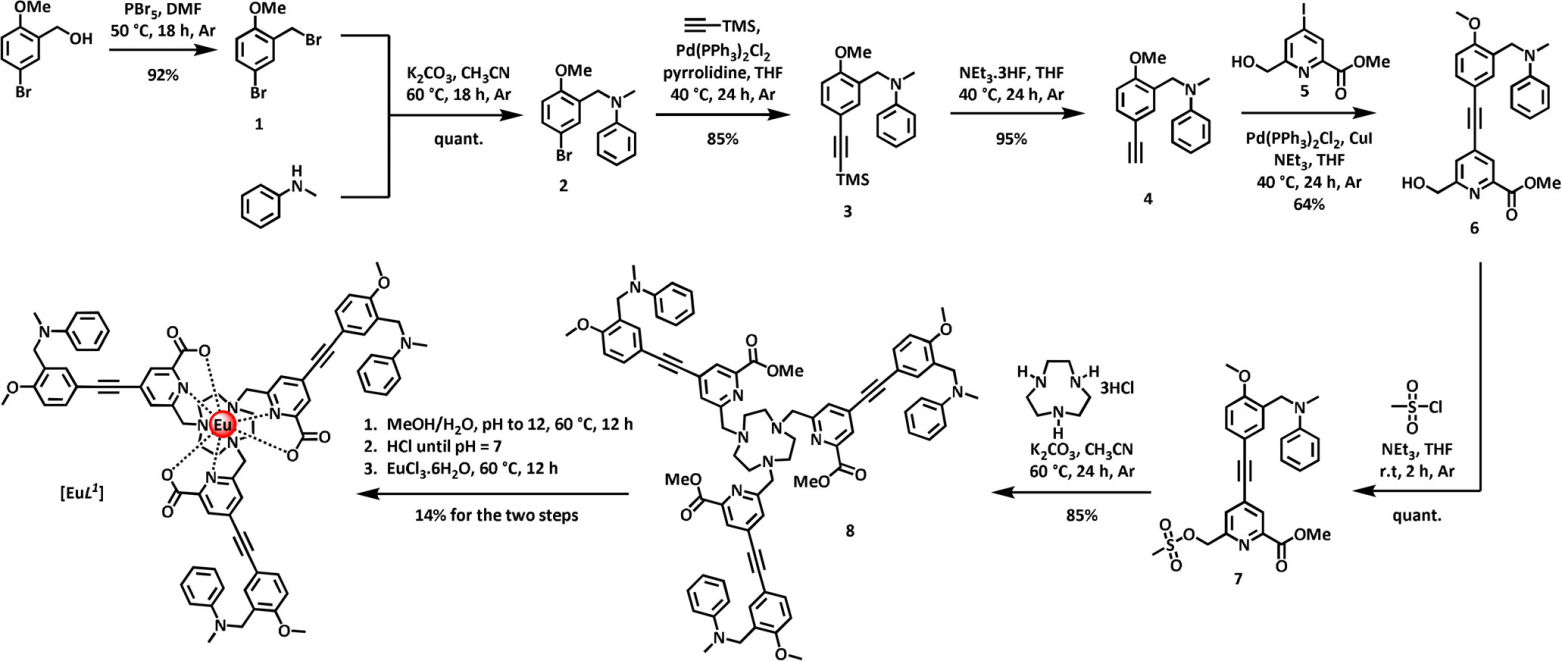
Synthesis of [Eu***L*** ^***1***^].

**Scheme 2:**
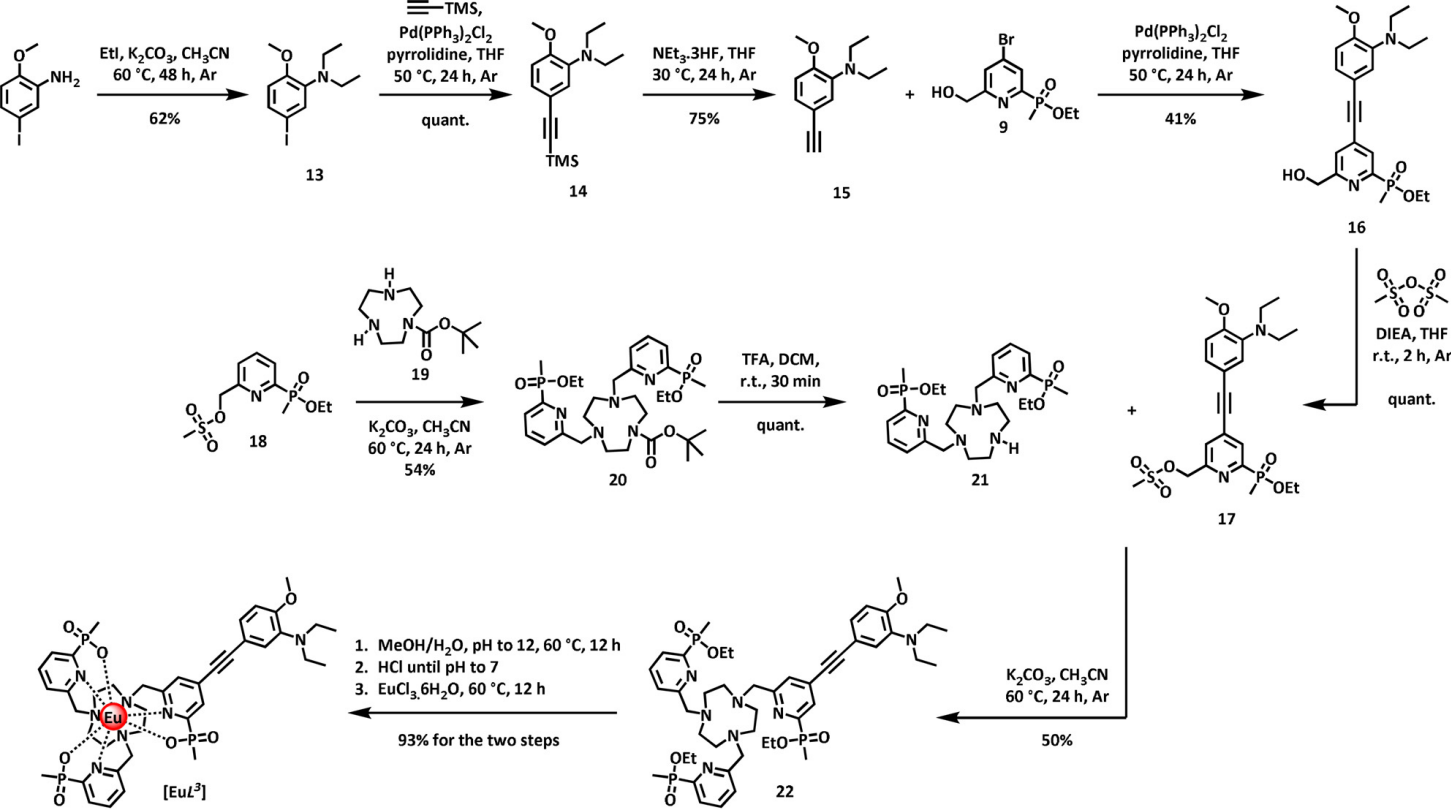
Synthesis of [Eu***L*** ^***3***^].

The synthesis of the target complexes [Eu***L*** ^***1***^] and [Eu***L*** ^***3***^] was undertaken as shown in Scheme 1 and Scheme 2. The structurally related complexes, [Eu***L*** ^***2***^] and [Eu***L*** ^***4***^], were made using similar methods and details are given in the Supporting Information.

Mono‐alkylation of *N*‐methylaniline with the substituted benzylic bromide, **1**, gave the tertiary amine **2**, which was coupled with trimethylsilylacetylene under palladium catalysis to afford the alkyne **3**, in 98 % yield. Following silyl deprotection with fluoride in THF, the terminal alkyne **4** was coupled with the 4‐iodo‐pyridine derivative **5**,^[15]^ in a copper(I) assisted reaction to give the conjugated alkyne **6**. Mesylation in THF afforded the sulfonate ester, **7**, which was used directly to alkylate triazacyclononane in acetonitrile to yield the C_3_ symmetric ligand precursor, **8**. Following base hydrolysis, [Eu***L*** ^***1***^] was prepared by reaction of the ligand with EuCl_3_ at ambient pH, and the neutral complex was purified by reversed‐phase HPLC (Scheme 1).

In the case of [Eu***L*** ^***3***^], the electron‐rich chromophore was introduced in the final alkylation step to avoid undue exposure to strong acid conditions during its preparation. The intermediate *N*,*N*‐diethyl‐substituted terminal alkyne, **15**, was made from 3‐amino‐4‐methoxy iodobenzene (Scheme 2) by a similar series of standard alkylation, coupling and deprotection steps, prior to a Sonagashira coupling reaction with the 4‐bromopyridine derivative, **9**, that has been reported earlier.^[16]^ The resultant disubstituted alkyne **16** was mesylated using methanesulfonic anhydride in THF in the presence of Hünig's base, to avoid the presence of chloride anion which can lead to unwanted displacement of the mesylate group, under certain conditions. In parallel, the diester, **20**, was prepared from the mesylate **18** ^[17]^ and mono‐BOC‐triazacyclononane **19**,^[18]^ and the Boc group removed by treatment with TFA. The resultant secondary amine **21**, was alkylated with the mesylate of the conjugated chromophore **17**, and the triethyl ester **22**, was hydrolysed in methanolic aqueous base followed by complexation with europium at ambient pH (Scheme 2). The desired charge neutral complex, [Eu***L*** ^***3***^] was purified by reversed‐phase HPLC.

### pH dependence of europium luminescence

**Figure 1:**
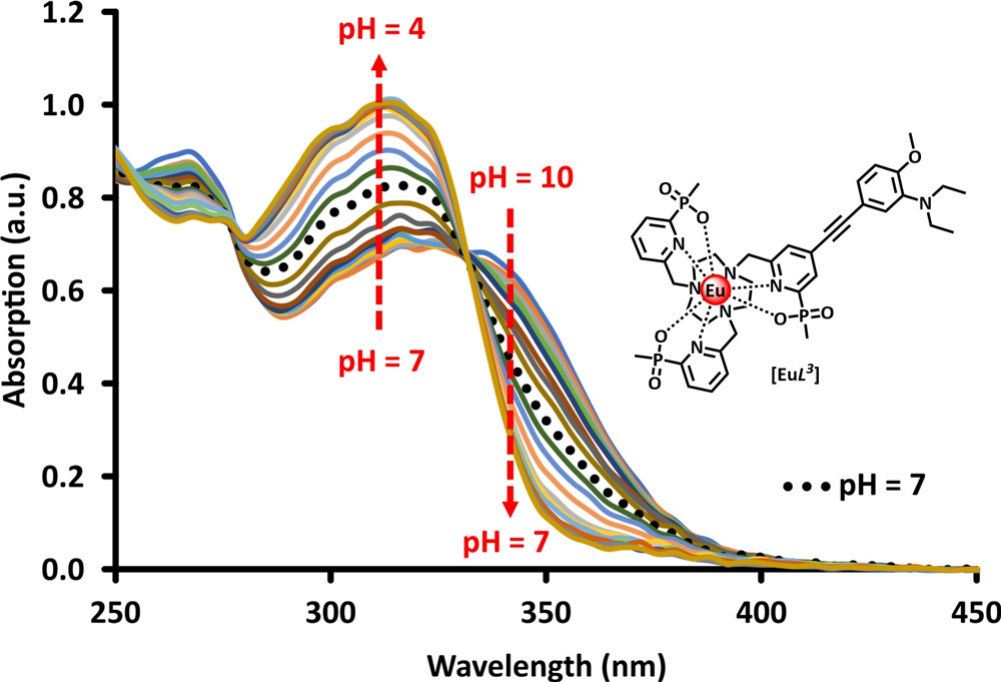
Variation of the absorption spectrum of [Eu***L*** ^***3***^] with pH revealing the isosbestic points at 278 and 331 nm (295 K, *c*_complex_=15 μm, 0.1 m NaCl).

**Figure 2:**
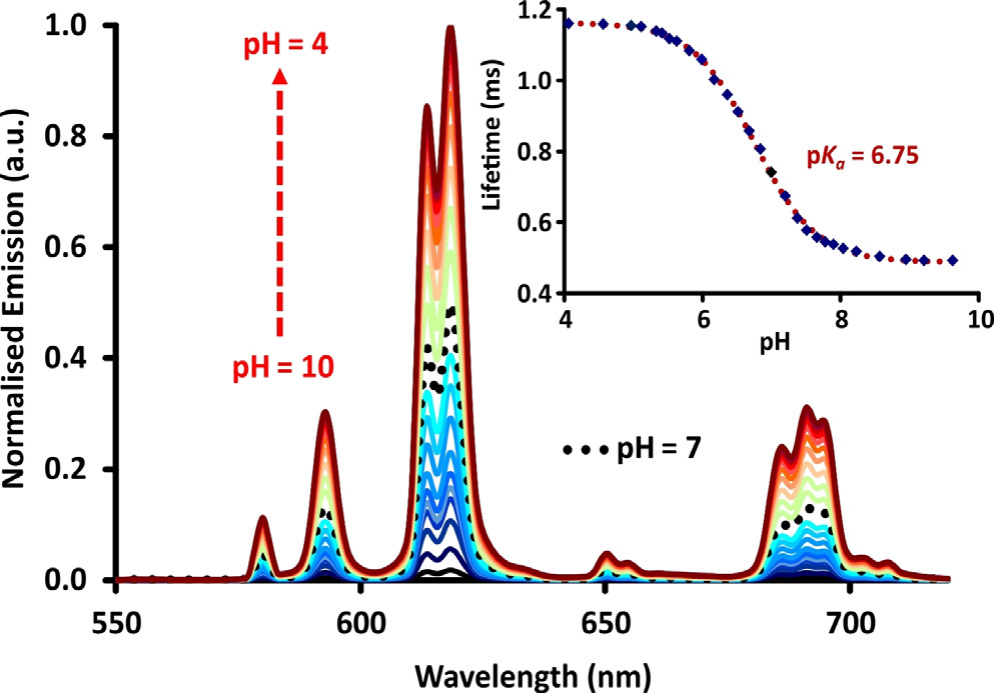
Variation of the europium emission spectrum and lifetime with pH for [Eu***L*** ^***3***^] (*λ*_exc_ 331 nm, 295 K, *c*_complex_=15 μm, 0.1 m NaCl) showing the fit (line) to the experimental data. Similar plots were obtained for excitation at 337 or 355 nm.

The pH dependence of the absorption, emission and excitation spectra for each complex was examined, together with measurements of the europium emission lifetime (Table 1). Considering first the complex [Eu***L*** ^***3***^], as it contains only one site of protonation at nitrogen. Protonation shifts the position of the main π–π* transition from 340 nm to 320 nm, characterised by the occurrence of two isosbestic points at 331 and 278 nm (Figure 1). The Eu emission intensity increased markedly as the pH was lowered from 10 to 4 (Figure 2) and was accompanied by an increase in lifetime from 0.45 ms to 1.16 ms. Overall emission quantum yields measured at pH 4 and pH 8 were 17 % and 0.5 % respectively. By fitting the variation of the Eu emission intensity or its lifetime with pH, a p*K*_a_ value was estimated by nonlinear least squares regression analysis. A value of 6.75(±0.05) was found (Table 2, 295 K, 0.1 m NaCl). Identical behaviour was found for both acidimetric and alkalimetric titrations consistent with full reversibility.

**Table 1:** Summary of photophysical properties for Eu^III^ complexes (295 K, *c*_complexes_=15 μm in 0.1 m NaCl).

Complex	*λ*_exc_ [nm]	*ϵ* [M^−1^⋅cm^−1^]	*τ* [ms]	*ϕ* [%]	*q*	*B* [M^−1^⋅cm^−1^]
[Eu***L*** ^***1***^]	324	8100^[a]^ 26 000^[b]^	0.70^[a]^ 0.65^[b]^	4.0^[a]^ 12.0^[b]^	0	324^[a]^ 3120^[b]^
[Eu***L*** ^***2***^]	340	21 000^[a]^ 34 200^[b]^	0.65^[a]^ 0.60^[b]^	12.0^[a]^ 18.7^[b]^	0	2520^[a]^ 6498^[b]^
[Eu***L*** ^***3***^]	331	12 000^[c]^	0.53^[a]^ 1.16^[b]^	0.5^[a]^ 17.0^[b]^	0	60^[a]^ 2040^[b]^
[Eu***L*** ^***4***^]	331	46 000^[a]^ 60 000^[b]^	0.25^[a]^ 0.84^[b]^	0.1^[a]^ 17.0^[b]^	0	46^[a]^ 10 200^[b]^

[a] Values at pH 8; [b] values at pH 4; [c] values at the isosbestic point. Experimental errors on lifetime are ±5 % and for quantum yields, ±15 %.

**Table 2:** Summary of p*K*_a_ values (±0.05) determined for [Eu***L*** ^***1–4***^] (*c*_complexes_=15 μm) in the stated media.

Complex	Conditions	p*K*_a_
[Eu***L*** ^***1***^]	0.1 m NaCl	n.d.^[a]^
[Eu***L*** ^***2***^]	0.1 m NaCl	6.48
[Eu***L*** ^***3***^]	0.1 m NaCl 0.1 m NaCl, 0.1 mm BSA	6.75 6.45
[Eu***L*** ^***4***^]	0.1 m NaCl 0.1 m NaCl, 0.1 mm BSA NIH‐3T3 cell lysate	6.21 6.20 5.75

[a] Not determined; see Supporting Information.

**Figure 3:**
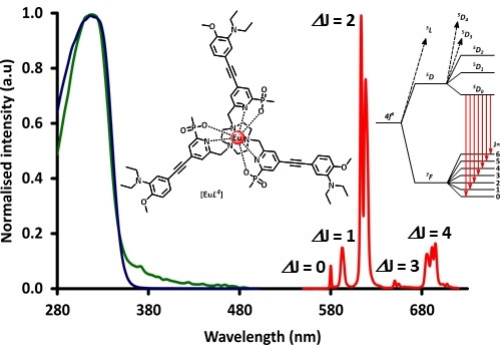
Absorption, excitation and metal‐based emission spectra for [Eu***L*** ^***4***^] (*λ*_exc_ 331 nm, 295 K, *c*_complex_=15 μm, 0.1 m NaCl), showing the highest‐energy transitions from the ^5^D_0_ excited state.

**Figure 4:**
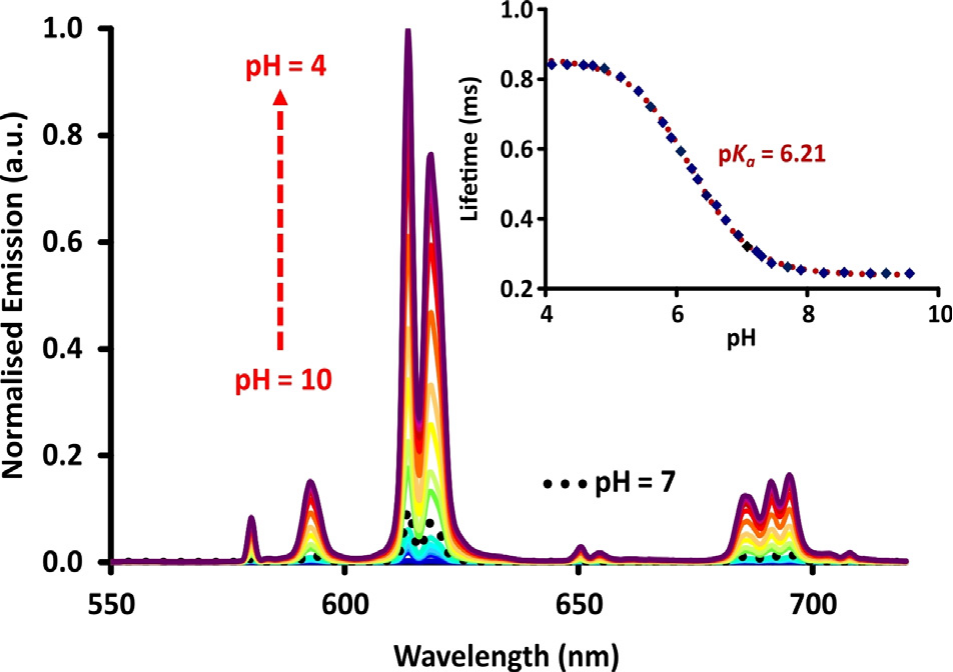
Variation of the europium emission spectrum and lifetime with pH for [Eu***L*** ^***4***^] (*λ*_exc_ 331 nm, 295 K, *c*_complex_=15 μm, 0.1 m NaCl) showing the fit (red) to the data points. Similar plots were obtained for excitation at 337 and 355 nm, or using emission intensity variations.

Similar overall behaviour was found in the pH dependence of [Eu***L*** ^***4***^], for which more complex absorption spectral changes occurred (Supporting Information). In this case, the emission quantum yield increased from 0.1 to 17 % from pH 8 to 4, and the lifetime increased from 0.24 to 0.84 ms (Figure 3 and Figure 4). The form of the Eu emission spectrum for [Eu***L*** ^***3***^] and [Eu***L*** ^***4***^] was more or less unchanged, with only minor changes observed in the hypersensitive Δ*J*=2 and 4 transitions, for example, a switching in the relative intensity of the two main bands in the region between 610 to 625 nm.

The variation of lifetime with pH allowed an apparent p*K*_a_ to be measured for [Eu***L*** ^***4***^] (Table 2). In this case, three successive protonation steps occur, but the data was fitted to a single protonation event. The sites of protonation are separated by at least 15 Å, and so can be considered to act independently. The apparent p*K*_a_ was 6.21(±0.05) in this case, and it did not change when 0.1 % bovine serum albumin solution was present, suggesting little interaction with this common serum protein. However, in a cell lysate background, the p*K*_a_ fell to 5.75.

The Eu complexes of ***L*** ^***1***^ and ***L*** ^***2***^ are sparingly soluble in water, and slowly came out of solution on standing at higher pH, with evidence for pH induced aggregation (Supporting Information Figures S3 and S5). However, each complex dissolved readily in methanol, in which they were strongly emissive, with overall photoluminescence quantum yields of 24 and 48 % respectively for the protonated species. In water, quantum yields were lower (4 and 12 % at pH 7; 12 and 19 % at pH 4), as found for related EuroTracker^TM^ probes that also possess a strong internal charge transfer transition.[^14a^, ^14b^, ^19^] The europium emission lifetime at pH 7 was 0.70 ms for each complex, and decreased by only 10 and 14 % respectively, between pH 8 and 4. At the same time, the Eu emission intensity showed an increase in intensity: for [Eu***L*** ^***1***^], the intensity increase was 330 % from pH 8 to 4, and for [Eu***L*** ^***2***^] the corresponding change was 55 %. Such behaviour accords with predominant suppression of photoinduced electron transfer of an intermediate ligand based excited state, following complex protonation. Given the inferior solubility and pH dependent switching properties of [Eu***L*** ^***1, 2***^], further studies focused on the properties of the complexes of ***L*** ^***3***^ and ***L*** ^***4***^, and [Eu***L*** ^***4***^] was selected for particular attention by virtue of its more favourable p*K*_a_ value, with a view to its use in cellulo.

**Figure 5:**
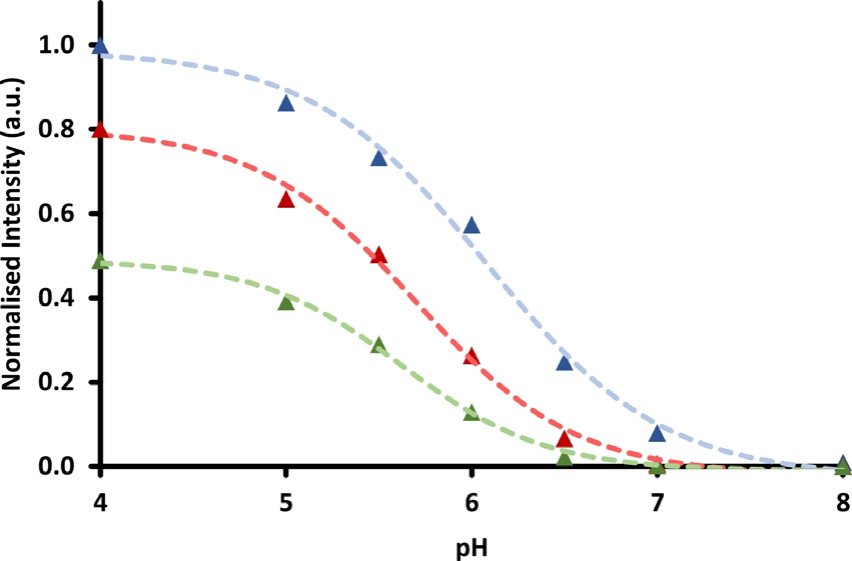
Emission intensity of [Eu***L*** ^***4***^] (*λ*_em_ 613 nm) as a function of pH with different time periods of acquisition (blue=60–460 μs, red=1000–2000 μs, green=1500–2500 μs). Data are normalised to a 60–460 μs time window at pH 4. Measurements were taken in aqueous solutions of NH_4_OAc (pH 4 and 5), MES (pH 5.5, 6, and 6.5), HEPES (pH 7) and NH_4_HCO_3_ (pH 8) buffers (*c*_complex_=15 μm, 0.1 m buffer in 0.1 m NaCl).

The large increase of the Eu emission lifetime that occurs on protonation with [Eu***L*** ^***4***^] allows the apparent switching ratio between the ‘on’ and ‘off’ states to be varied, by changing the time delay in signal acquisition. Two cases were investigated in which either a fixed time delay was introduced, or a different time window for spectral acquisition was selected (Table 3 and Figure 5). The emission intensities were measured in buffered solution at pH 4 and pH 8, and the ratios of the overall europium emission intensities were measured in each case. By changing the delay time from 0.06 ms to 1 ms, the ‘switch‐on’ factor increased from 250 to 650, with an apparent p*K*_a_ of 5.7(±0.1) under these different sets of experimental conditions. By choosing different time windows, a slightly greater increase in this factor was found, with the maximum ratio found for the time window 1.5 to 2.5 ms, notwithstanding the decrease in overall signal intensity that occurs using an acquisition period that is rather longer than the emission lifetime. Such behaviour augurs well for such conditions to be employed in either a time‐gated assay, or in future time‐resolved microscopy studies.

**Table 3:** Ratios of emission intensities (‘switch‐on’ factors) for the stated single gate delay times or differing time gate periods, showing the effect on the apparent p*K*_a_ values.^[a]^

*t* [μs]	pH 4/pH 8	app. p*K*_a_
*single gate delay times*		
60	250	5.8
460	623	5.7
1000	650	5.6
*differing time gate periods*		
60–460	111	5.8
1000–2000	560	5.7
1500–2500	1334	5.6

[a] 295 K, *c*=15 μm, 0.1 m NaCl; buffers, NH_4_OAc (0.1 m, pH 4), NH_4_HCO_3_ (0.1 m, pH 8).

### Cell imaging studies and control experiments

**Figure 6:**
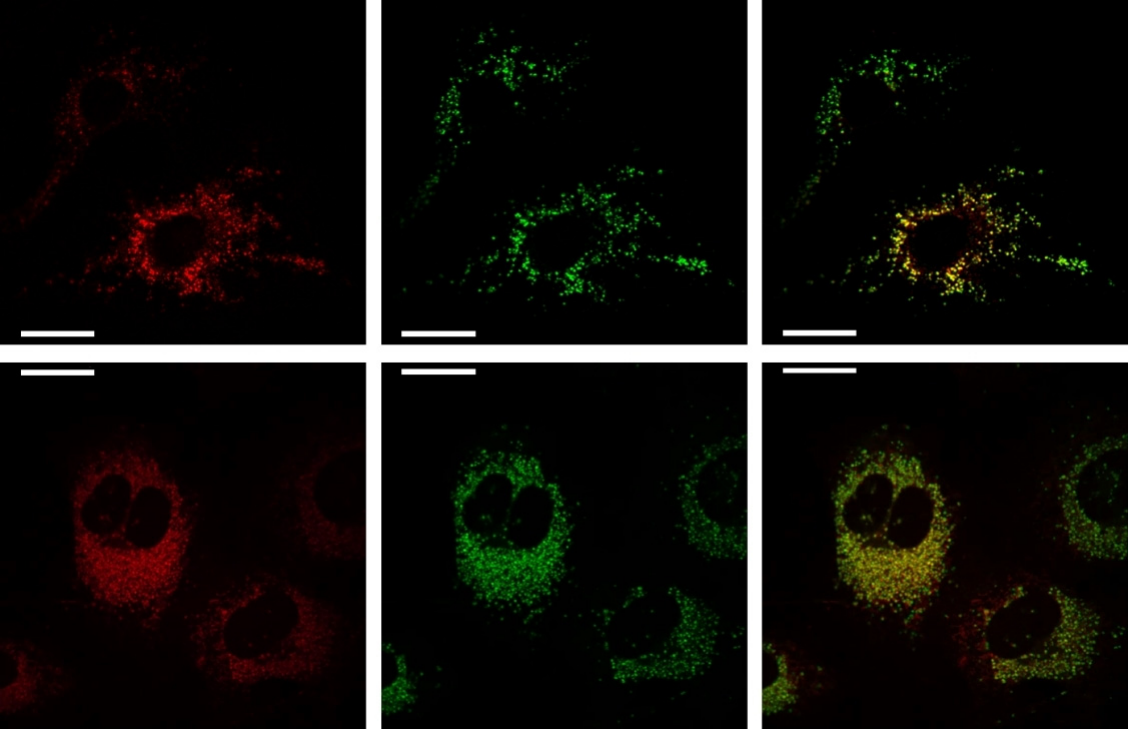
Live cell images (NIH‐3T3) with [Eu***L*** ^***4***^] after 16 h. Left: [Eu***L*** ^***4***^] (*c*_complex_=30 μm, *λ*_exc_=355 nm, *λ*_em_=600–720 nm); Centre: Lysotracker Green (*λ*_exc_=488 nm, *λ*_em_=500–530 nm); Right: overlay shows co‐localisation (P>0.95). Note the observation of normal cell division at 16 h.

Cell uptake and co‐localisation studies were undertaken for [Eu***L*** ^***4***^] in living mouse skin fibroblasts (NIH‐3T3) and human breast cancer cells (MCF‐7), following methods previously reported.[^3b^, ^14c^, ^19^] The extent of complex uptake was monitored by assessing image brightness as a function of time. In parallel, complex concentrations at fixed time points were measured by analysing the Eu concentration in these cell samples, using ICP‐mass spectrometry (Figure S11). Co‐staining experiments using Lysotracker Green (Figure 6), verified that the complex was localised in the lysosomes, with higher correlation coefficients as probe brightness increased, within a given cell cycle.

The microscopy study revealed a time dependent increase in brightness that maximised at around 16 to 18 hours. Such behaviour coincides with the time period of mitosis for NIH‐3T3 cells; during unaltered proliferation, they divide every 16 hours. In a separate study, cell toxicity was assessed using live/dead assay image cytometry (Supporting Information), and no evidence (±5 %) for perturbation of natural cell homeostatic function and proliferation was found over a period of 24 hours, for concentrations of up to 100 μm.

**Figure 7:**
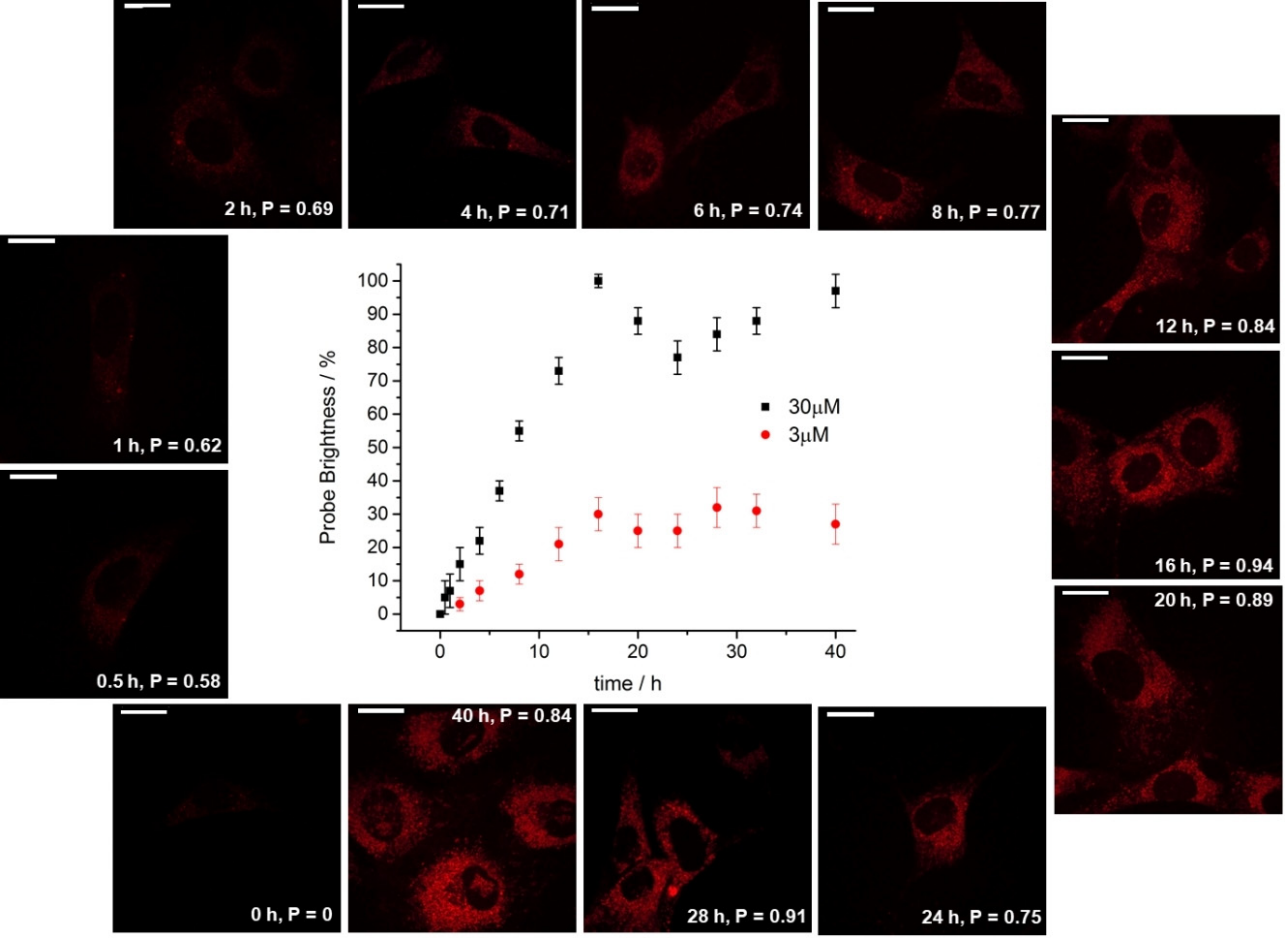
Brightness profile of [Eu***L*** ^***4***^] as a function of time for incubation concentrations of 30 μm (black) and 3 μm (red) in living NIH‐3T3 cells; images shown are for 30 μm. Scale bar: 20 μm. The cells were monitored for 72 h, when the cells were proliferating normally.

After cell division, the daughter cells appeared less bright, in line with the increase in cell volume. Each daughter cell possesses about 50 % of the internalised complex of the parent cell, as they begin their independent life cycle. The concentration of complex found inside the cell then increases again, as the daughter cells mature and embark on homeostatic uptake of nutrients and [Eu***L*** ^***4***^] present in the cell growth medium. However, this process has a time lag, resulting in the observed time vs. brightness profile (Figure 7). Similar overall behaviour was found in a separate study where the concentration of the Eu complex was 10 times lower.

**Figure 8:**
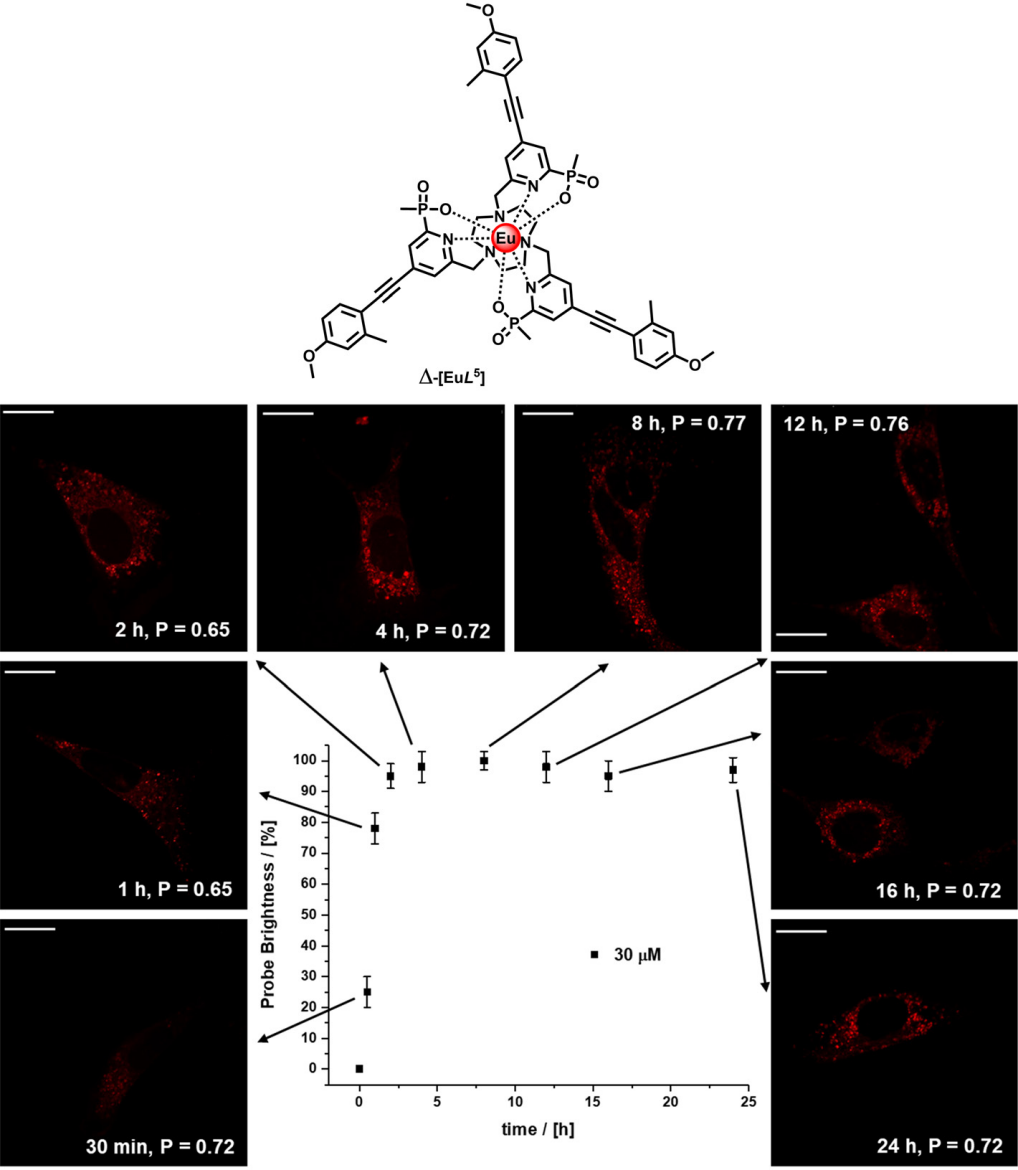
Brightness profile of *Δ*‐[Eu***L*** ^5^] as a function of time in live NIH‐3T3 cells; images shown are for a 30 μm incubation. Scale bar: 20 μm. The cells were monitored for 48 h, and were proliferating normally.

In a control experiment with a structurally analogous complex, *Δ*‐[Eu***L*** ^***5***^], that shows no pH dependent behaviour and a predominant lysosomal staining pattern, very different behaviour was observed.^[20]^ In this case (Figure 8), under the same incubation conditions in NIH‐3T3 cells, the mean image brightness reached a plateau after 4 h, and remained constant thereafter, with a minor inflection around 16–18 h. Such a kinetic intensity profile is characteristic of the EuroTracker series of Eu complexes, studied in depth for several living cells.[^14^, ^19^]

**Figure 9:**
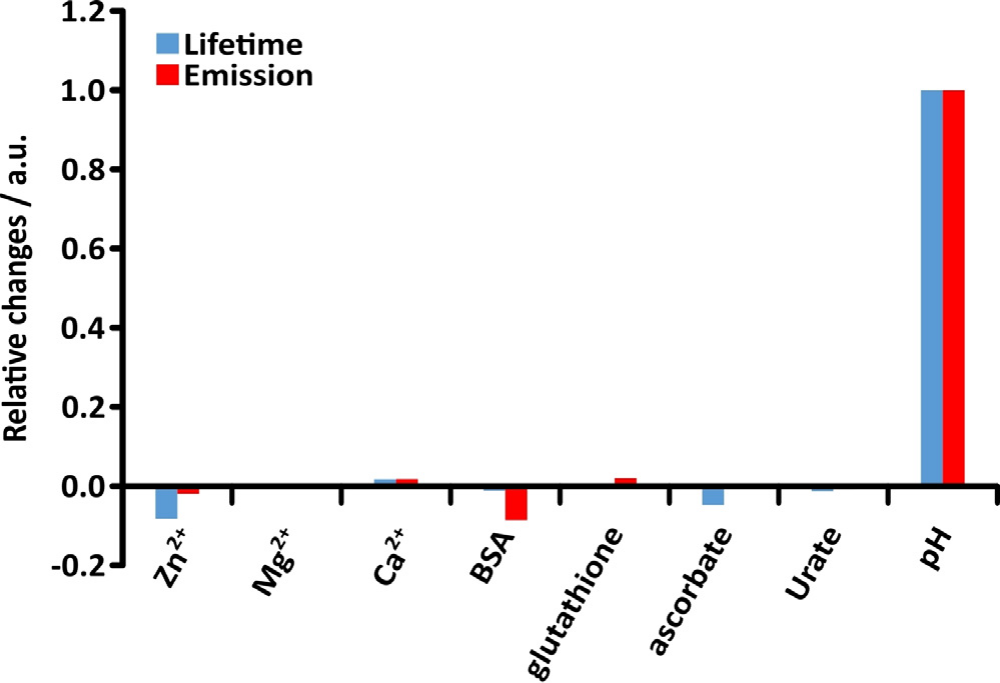
Total change in emission lifetime (blue) and emission intensity (red) following addition of 5 equivalents of analyte to [Eu***L*** ^4^] (*c*_complex_=30 μm, 0.1 m NaCl, 0.1 m NH_4_HCO_3_ buffer, pH 5) relative to the pH change observed from 8 to 4 (normalised to unity). Lifetime and intensity data are given in Figure S8.

A series of control experiments in vitro was also undertaken, in order to verify that the changes in image brightness in the cell imaging work were not due to any excited state quenching by common endogenous species whose concentration could hypothetically change in the same time period in the given organelles. Accordingly, the Eu lifetime and observed emission intensity were measured for [Eu***L*** ^***4***^] in the presence of increasing concentrations of different species that are likely to be present inside the cell compartment. No significant changes in lifetime or intensity were observed following addition of up to 5 equivalents (corresponding to a limiting 150 micromolar concentration) of Mg, Ca and Zn ions. Similarly, no changes were found, within experimental error, for addition of the endogenous reductants ascorbate, urate and glutathione that can potentially quench the probe excited state by electron transfer. Finally, no major variations (±10 %) were found for added bovine serum albumin that is present in the cell incubation medium. The insensitivity to change compared with the direct dependence on local pH is highlighted below (Figure 9).

The time dependent increase in brightness in the cell experiments with [Eu***L*** ^***4***^] in theory can be attributed both to changes in the extent of uptake (likely proceeding via macropinocytosis as found in related studies),[^3b^, ^20^] and the pH drop in the ageing endosomes as they transform into lysosomes. Control experiments measuring the intracellular concentration of the Eu‐complex directly using ICP‐MS were therefore undertaken at 2, 4, 8, 12, 16 and 24 hours (Figure S11). These studies showed a significantly less steep increase, with the intracellular Eu concentration doubling between 2 and 16 hours, while the observed image brightness increased by a factor of 50.

Such behaviour is consistent with the idea that the brightness enhancement is not simply due to the slow increase in the total amount of complex internalised, but directly relates to the effect of diminishing pH in the ageing endosomes/lysosomes, with the associated large increase of Eu emission intensity as the percentage of the more emissive protonated complex increases. This hypothesis is also consistent with the observed time dependent increase in Pearson's co‐localisation coefficient (P), in the parallel microscopy study using LysoTracker Green (Figure 7).

After a separate 16 hours incubation in NIH‐3T3 cells with [Eu***L*** ^***4***^] (30 μm), nigericin (200 nm) was added. This is an agent that has been shown to increase lysosomal pH quickly, from ∼4.5 to 6.5. After 5 minutes, confocal microscopy studies showed that the Eu complex appeared to be considerably less bright overall, consistent with the increased size of the treated cell's lysosomes and the decreased probe brightness at the higher pH value (Figure S9). In an additional control experiment, the internalised complex concentration of both treated and untreated cells, for a given time incubation time point, was found to be identical within experimental error, using ICP‐MS. Parallel results were observed, in terms of localisation behaviour and time‐dependent brightness change, using the MCF‐7 breast cancer cell line. An MTT assay assessment was also undertaken, with no evidence found for cellular toxicity with [Eu***L*** ^***4***^] monitoring mitochondrial redox status for 24 h over the concentration range 10 to 100 μm (Figure S10).

It should be noted in a word of caution, that the limitations of the experimental approach where additions of agents that greatly perturb cellular permeability have been noted.[^3b^, ^21^] The use of the fluorescent LysoSensor probe for example, has been advocated for lysosomal pH assessment, but it is only useful for very short perturbational periods, as it induces changes itself on the local pH and is only applicable to imaging studies carried out over a few minutes,^[21a]^ or in dead (fixed) cells. Studies with fixed cells have very clear limitations with respect to the monitoring of living processes. The observations reported here, of the continuous monitoring of cellular acidification over a period of up to 24 h in live cells, have no obvious precedent for a pH sensitive phosphorescent probe that has no apparent effect on cellular homeostasis.

## Conclusions

Four new luminescent Eu^III^ complexes have been prepared and evaluated in a series of comparative studies, assessing their absorption and emission spectral behaviour as a function of pH. The Eu complexes of ***L*** ^***1***^ and ***L*** ^***2***^, where the site of protonation is separated from the main chromophore by a methylene group, showed no significant variation in absorption spectral form and exhibited the smallest changes in emission intensity and lifetime. In contrast, the protonation of complexes of ***L*** ^***3***^ and ***L*** ^***4***^, where the amine group is conjugated into the phenyl ring, caused a hypsochromic shift in the main absorption band and an increase in the Eu lifetime of 120 and 240 % respectively. By varying the delay time or the time period for acquisition of Eu emission spectral intensity data, intensity ratios of between 250 and 1300 were measured for [Eu***L*** ^***4***^], comparing pH 4 and pH 8 samples. The Eu emission lifetime and intensity was more or less invariant to the concentration of Ca, Mg and Zn ions, to the natural electron‐rich reductants ascorbate, urate and glutathione and to the protein BSA, each of which may potentially perturb the behaviour of a probe luminophore in the living cell. Such large pH induced ‘switching on’ factors are well suited to assays where acidification needs to be monitored, as occurs during the maturation of endosomes or in the ageing of lysosomes, following uptake of a labelled protein or antibody.

The complex [Eu***L*** ^***4***^] was examined in two different cell lines and was found to localise mainly in the cellular lysosomes, without compromising cellular viability or proliferation. Using laser scanning confocal microscopy, exciting the complex at 355 nm, an increase in probe brightness was observed in the living cells as a function of time, over the period 2 h to 16 h. The observed brightness was 25 times greater than the change associated with the independently measured increase in probe uptake. In control experiments with a related complex, [Eu***L*** ^***5***^], that exhibits no dependence of emission on pH, very different behaviour was observed and no significant changes in image brightness were observed beyond the dependence on the extent of uptake. Taken together, such behaviour is consistent with the monitoring of acidification of the cell's lysosomes as they age using this pH sensitive probe. Given that the cells were healthy, cell division occurred after 16–18 h and after a dip in probe brightness, the daughter cells continued to take up the complex and their lysosomes were subsequently observed to reach maturation.

Thus, the complex [Eu***L*** ^***4***^] is well suited as the basis of probe design for new systems that can be linked to targeting vectors and antibodies to monitor receptor internalisation. Such new derivatives can be used to tag proteins selectively, to monitor the time‐dependence of uptake into more acidic organelles and their recycling, in order to enhance the performance of current state‐of‐the‐art, time‐resolved internalisation assays.^[22]^ Such studies have been undertaken and the promising behaviour of these pH sensitive probes will be reported in due course.

## Experimental Section

Details of general experimental methods, instrumentation, cell microscopy and the photophysical techniques used are given in the Supporting Information. Methyl 6‐(hydroxymethyl)‐4‐iodopicolinate **5**,^[15]^ 4‐bromo‐2‐hydroxymethyl‐6‐methylphosphinate pyridine **9**,^[16]^ (6‐(ethoxy(methyl)phosphoryl)pyridin‐2‐yl)methyl methanesulfonate) **18**,^[17]^ and *tert*‐butyl 1,4,7‐triazacyclonane‐1‐carboxylate **19**,^[18]^ were prepared using reported procedures. Details of the syntheses of [Eu***L*** ^***2***^] and [Eu***L*** ^***4***^] are given in the Supporting Information, together with spectral data for ligand intermediates, additional spectral information for each complex, and LC–MS data for the Eu complexes.

**4‐Bromo‐2‐(bromomethyl)‐1‐methoxybenzene, 1**: To a solution of (5‐bromo‐2‐methoxy‐benzyl alcohol (623 mg, 2.87 mmol) dissolved in anhydrous DMF (15 mL) under argon was added PBr_5_ (1.85 g, 4.32 mmol). The solution was stirred at 50 °C for 18 h under argon. Solvent was removed under reduced pressure to yield an orange oil. The residue was dissolved in CH_2_Cl_2_ (20 mL) and water (20 mL) was added. The aqueous layer was extracted with CH_2_Cl_2_ (3×10 mL), and the combined organic layers were dried over Na_2_SO_4_, filtered and concentrated to dryness to yield a light‐yellow oil that was purified by column chromatography (SiO_2_, CH_2_Cl_2_ 100 %) to yield a white solid (740 mg, 92 %); *R*_f_=0.76 (SiO_2_, 100 % CH_2_Cl_2_); mp: 114–116 °C; ^1^H NMR (298 K, 400 MHz, CDCl_3_): *δ*_H_=7.47 (1 H, d, *J=*2.5 Hz), 7.40 (1 H, dd, *J=*9.0 Hz, *J=*2.5 Hz), 6.78 (1 H, d, *J=*9.0 Hz), 4.48 (2 H, s), 3.88 (3 H, s); ^13^C NMR (298 K, 100 MHz, CDCl_3_): *δ*_C_=156.6, 133.6, 132.8, 128.3, 112.8, 112.7, 56.0, 27.7; (GC–MS) *t*_R_=4.46 min, *m*/*z* 279.950 [*M* ^+^], 199.000 [*M*−Br]^+^⋅, elemental analysis calculated for C_8_H_8_Br_2_O (%): C=34.32, H=2.88; found: C=34.09, H=2.88.

***N*** **‐(5‐Bromo‐2‐methoxybenzyl)‐*N*‐methylaniline, 2**: Compound **1** (78 mg, 0.28 mmol) was dissolved in dry CH_3_CN (2 mL). *N*‐Methyl aniline (31 μL, 0.28 mmol) and K_2_CO_3_ (78 mg, 0.56 mmol) were added under argon. The reaction was stirred at 50 °C for 24 h under argon and progress was monitored by LC–MS. The excess inorganic salts were removed by filtration and the filtrate was evaporated to dryness. The residue was dissolved in a CH_2_Cl_2_/water solution (20 mL, 1:1) and the aqueous layer was extracted with CH_2_Cl_2_ (3×10 mL). The combined organic layers were dried over Na_2_SO_4_, filtered and concentrated to dryness affording a colourless oil (80 mg, 94 %). NMR analysis and LC–MS shown that compound **2** was sufficiently pure to be used in the next step without further purification; *R*_f_=0.90 (SiO_2_, 100 % CH_2_Cl_2_); ^1^H NMR (298 K, 400 MHz, CDCl_3_): δ_H_=7.42 (1 H, d, *J=*2.5 Hz), 7.36–7.28 (3 H, m), 6.90–6.76 (4 H, m), 4.57 (2 H, s), 3.92 (3 H, s), 3.13 (3 H, s); ^13^C NMR (298 K, 100 MHz, CDCl_3_): *δ*_C_=156.2, 149.6, 130.4, 129.8, 129.3, 129.2, 116.6, 113.1, 112.1, 111.8, 55.5, 52.0, 38.7; (HRMS+) *m*/*z* 306.0490 [*M*+H]^+^ (C_15_H_17_NOBr requires 306.0494).

***N*** **‐(2‐Methoxy‐5‐((trimethylsilyl)ethynyl)benzyl)‐*N*‐methylaniline, 3**: Compound **2** (105 mg, 0.34 mmol) was dissolved in dry THF (3 mL) followed by addition of Pd(PPh_3_)_2_Cl_2_ (35 mg, 0.05 mmol), TMS‐acetylene (250 μL, 1.7 mmol) and pyrrolidine (168 μL, 2 mmol) under argon. The reaction was stirred at 50 °C for 48 h under argon. After LC–MS analysis indicated reaction completion, solvent was removed under reduced pressure and the residual dark‐brown oil was dissolved in CH_2_Cl_2_/water (40 mL, 1:1). The aqueous layer was extracted with CH_2_Cl_2_ (3×10 mL) and the combined organic layers were dried over Na_2_SO_4_, filtered and concentrated to dryness to give a crude dark‐brown oil that was purified by column chromatography (SiO_2_, hexane to EtOAc 2 % in hexane) to afford a yellow oil (108 mg, 98 %); *R*_f_=0.32 (SiO_2_, 2 % EtOAc in hexane); ^1^H NMR (298 K, 400 MHz, CDCl_3_): *δ*_H_=7.40 (1 H, dd, *J=*8.0 Hz, *J=*2.0 Hz), 7.28–7.21 (3 H, m), 6.83 (1 H, d, *J=*8.0 Hz), 6.78–6.68 (3 H, m), 4.46 (2 H, s), 3.88 (3 H, s), 3.05 (3 H, s), 0.23 (9 H, s); ^13^C NMR (298 K, 100 MHz, CDCl_3_): *δ*_C_=157.4, 149.8, 132.3, 130.6, 129.1, 127.0, 116.3, 115.1, 112.1, 109.8, 105.5, 92.3, 55.4, 52.2, 38.5, 0.1; (HRMS+) *m*/*z* 324.1779 [*M*+H]^+^ (C_20_H_26_NOSi requires 324.1784).

***N*** **‐(5‐Ethynyl‐2‐methoxybenzyl)‐*N*‐methylaniline, 4**: Compound **3** (335 mg, 1.04 mmol) was dissolved in dry THF (4.5 mL) before NEt_3_⋅3 HF (4.5 mL, 24 mmol) was added. The solution was stirred at 30 °C for 24 h under argon, when solvent was removed under reduced pressure and the residual oil was dissolved in CH_2_Cl_2_/water (40 mL, 1:1). The aqueous layer was extracted with CH_2_Cl_2_ (3×20 mL) and the combined organic layers were dried over Na_2_SO_4_, filtered and concentrated to dryness to yield a light‐orange oil. The residual oil was purified by column chromatography (SiO_2_, hexane to 2 % EtOAc in hexane) to yield a light‐yellow oil (255 mg, 98 %); *R*_f_=0.25 (SiO_2_, 2 % EtOAc in hexane); ^1^H NMR (298 K, 400 MHz, CDCl_3_): *δ*_H_=7.42 (1 H, dd, *J=*8.0 Hz, *J=*2.0 Hz), 7.28–7.21 (3 H, m), 6.85 (1 H, d, *J=*8.0 Hz), 6.78–6.68 (3 H, m), 4.49 (2 H, s), 3.90 (3 H, s), 3.07 (3 H, s), 2.96 (1 H, s); ^13^C NMR (298 K, 100 MHz, CDCl_3_): *δ*_C_=157.6, 149.6, 132.3, 130.9, 129.2, 127.2, 116.4, 114.0, 112.1, 109.9, 84.0, 75.7, 55.4, 52.0, 38.6; (HRMS+) *m*/*z* 252.1383 [*M*+H]^+^ (C_17_H_18_NO requires 252.1388).

**Methyl‐6‐(hydroxymethyl)‐4‐((4‐methoxy‐3‐((methyl(phenyl)amino)methyl)phenyl)ethynyl)picolinate, 6**: Methyl 6‐(hydroxymethyl)‐4‐iodopicolinate **5** (64 mg, 0.22 mmol) was dissolved in dry THF (1 mL) and NEt_3_ (150 μL, 1.1 mmol) was added. The solution was degassed three times (freeze–thaw cycles). Compound **4** (60 mg, 0.24 mmol) and Pd(PPh_3_)_2_Cl_2_ (21 mg, 0.03 mmol) were added and the solution was degassed once more. CuI (11.5 mg, 0.06 mmol) was added and the reaction was stirred at 60 °C for 24 h under argon. After this time, solvent was removed under reduced pressure and the residual oil was purified by column chromatography (SiO_2_, CH_2_Cl_2_ to 2 % MeOH in CH_2_Cl_2_) to yield a red oil (58 mg, 64 %); *R*_f_=0.30 (SiO_2_, 2 % MeOH in CH_2_Cl_2_); ^1^H NMR (298 K, 400 MHz, CDCl_3_): *δ*_H_=8.07 (1 H, s), 7.60 (1 H, s), 7.49 (1 H, dd, *J=*8.0 Hz, *J=*2.0 Hz), 7.33 (1 H, d, *J=*2 Hz), 7.28–7.23 (2 H, m), 6.92 (1 H, d, *J=*8.0 Hz), 6.78–6.70 (3 H, m), 4.86 (2 H, s), 4.51 (2 H, s), 4.00 (3 H, s), 3.93 (3 H, s), 3.65 (1 H, br. s), 3.10 (3 H, s); ^13^C NMR (298 K, 100 MHz, CDCl_3_): *δ*_C_=165.3, 160.6, 158.3, 149.6, 146.9, 133.9, 132.3, 130.6, 129.2, 128.7, 128.5, 127.6, 116.4, 113.6, 112.0, 110.2, 96.3, 85.1, 64.5, 55.5, 53.0, 52.0, 38.7; (HRMS+) *m*/*z* 417.1807 [*M*+H]^+^ (C_25_H_25_N_2_O_4_ requires 417.1814).

**Methyl‐4‐((4‐methoxy‐3‐((methyl(phenyl)amino)methyl)phenyl)ethynyl)‐6‐(((methylsulfonyl)oxy)methyl)picolinate, 7**: The alcohol **6** (30 mg, 72 μmol) was dissolved in anhydrous THF (1 mL) under argon. Mesyl chloride (8 μL, 108 μmol) and NEt_3_ (34 μL, 252 μmol) were added under argon and the reaction was stirred at ambient temperature for 2 h. After this time, solvent was removed under reduced pressure and the residue was dissolved in CH_2_Cl_2_/water (1:1, 20 mL). The aqueous layer was extracted with CH_2_Cl_2_ (3×10 mL). The combined organic layers were dried over Na_2_SO_4_, filtered and concentrated to dryness to yield a pale‐pink oil (35.7 mg, quant) that was sufficiently pure to be used for the next step without further purification; ^1^H NMR (298 K, 400 MHz, CDCl_3_): *δ*_H_=8.15 (1 H, d, *J=*2.0 Hz), 7.70 (1 H, d, *J=*2.0 Hz), 7.51 (1 H, dd, *J=*8.0 Hz, *J=*2 Hz), 7.35 (1 H, d, *J=*2.0 Hz), 7.26 (2 H, td, *J=*7.0, Hz, *J* ^2^=2 Hz), 6.93 (1 H, d, *J=*8.0 Hz), 6.79–6.69 (3 H, m), 5.41 (2 H, s), 4.51 (2 H, s), 4.02 (3 H, s), 3.94 (3 H, s), 3.17 (3 H, s), 3.11 (3 H, s). UPLC (ES^+^ MS, CH_3_CN/H_2_O, 0.1 % formic acid) *t*_R_=3.40 min, *m*/*z* 496.517 (69 %, [*M*+H]^+^), 989.170 (100 %, [2 m]^+^).

**Trimethyl‐6,6′,6′′‐((1,4,7‐triazacyclononane‐1,4,7‐triyl)tris(methylene))tris(4‐((4‐methoxy‐3‐((methyl(phenyl)amino)methyl)phenyl)ethynyl)picolinate), 8**: 1,4,7‐Triazacyclononane trihydrochloride (5.7 mg, 24 μmol) and the mesylate **7** (35.7 mg, 72 μmol) were dissolved in anhydrous CH_3_CN (2 mL) and K_2_CO_3_ (20.0 mg, 144 μmol) was added. The mixture was stirred at 60 °C for 24 h under argon. The excess potassium salts were removed by filtration and the filtrate was concentrated to dryness to yield an orange oil (27 mg, 85 %). This compound was used directly in the next step without further purification; ^1^H NMR (298 K, 400 MHz, CDCl_3_): *δ*_H_=8.01 (3 H, d, *J=*2.0 Hz), 7.82 (3 H, br. s), 7.45 (3 H, dd, *J* ^1^=8.0 Hz, *J=*2 Hz), 7.31 (1 H, d, *J=*2.0 Hz), 7.21 (6 H, td, *J=*7.0, Hz, *J=*2 Hz), 6.89 (3 H, d, *J=*8.0 Hz), 6.71–6.68 (9 H, m), 4.48 (6 H, s), 3.96 (6 H, s), 3.92 (18 H, s), 3.05 (9 H, s), 2.92 (12 H, br. s); ^13^C NMR (298 K, 100 MHz, CDCl_3_): *δ*_C_=165.5, 158.1, 149.6, 147.3, 132.3, 132.0, 130.6, 129.1, 128.6, 128.5, 127.9, 127.5, 116.4, 113.8, 112.0, 110.1, 95.7, 85.3, 64.1, 55.5, 52.9, 52.1, 38.6, 31.9; UPLC (CH_3_CN/H_2_O, 0.1 % formic acid) *t*_R_=3.59 min; (HRMS+) *m*/*z* 1324.622 [*M*+H]^+^ (C_81_H_82_N_9_O_9_ requires 1324.624).

**[EuL^1^]**: Compound **8** (27 mg, 20.4 μmol) was dissolved in a solution of CH_3_OH (1 mL), and a solution of aqueous sodium hydroxide (1 mL, 0.1 m) was added to reach pH 12. The solution was heated at 60 °C overnight, before it was adjusted to pH 7 by addition of a 1 m HCl solution. EuCl_3_⋅6 H_2_O (8.5 mg, 23 μmol) was added and the solution was stirred at 60 °C for 12 h. After this time, the solution was removed under reduced pressure to yield the crude Eu^III^ complex as a yellow solid (bright red under long‐wave UV light). The complex was purified by reversed‐phase HPLC (CH_3_CN/25 mm ammonium bicarbonate buffer) to give a yellow powder (4 mg, 14 %) after freeze drying; UPLC (CH_3_CN/H_2_O, 0.1 % formic acid) *t*_R_=3.65 min; (HRMS+) *m*/*z* 1430.471 [*M*+H]^+^ (C_78_H_73_N_9_O_9_^151^Eu requires 1430.473); *τ*_H2O_=0.70 ms (pH 7); *τ*_D2O_=0.79 ms; *τ*_MeOH_=0.85 ms; *q*=0; *λ*_max_=340 nm; *ϵ*_340_ n_m_=34 200 m ^−1^⋅cm^−1^ (pH 7); *Φ*_H2O_=4.0 % (pH 7), *Φ*_MeOH_=24 %.

***N***,***N*** **‐Diethyl‐5‐iodo‐2‐methoxyaniline, 13**: 2‐Iodo‐2‐methoxyaniline (2.83 g, 8.03 mmol) was dissolved in anhydrous CH_3_CN (15 mL) and iodoethane (7 mL, 87 mmol) and K_2_CO_3_ (6.3 g, 45.6 mmol) were added under argon. The reaction mixture was heated at 60 °C for 48 h. After total conversion of the starting material, the excess of K_2_CO_3_ was removed by filtration and the filtrate was concentrated to dryness. The residual oil was dissolved in CH_2_Cl_2_/water (40 mL, 1:1), and the aqueous layer was extracted with CH_2_Cl_2_ (3×20 mL). The combined organic layers were dried over Na_2_SO_4_, filtered and concentrated to dryness to give a light‐yellow oil that was purified by column chromatography (SiO_2_, 100 % CH_2_Cl_2_ to 2 % MeOH in CH_2_Cl_2_) to afford a colourless oil (2.16 g, 62 %); *R*_f_=0.18 (SiO_2_, 100 % CH_2_Cl_2_). ^1^H NMR (298 K, 400 MHz, CDCl_3_): *δ*_H_=7.26 (1 H, dd, *J=*8.0 Hz, *J=*2.0 Hz), 7.17 (1 H, d, *J=*2.0 Hz), 6.60 (1 H, d, *J=*8.0 Hz), 3.83 (3 H, s), 3.14 (4 H, q, *J=*7.0 Hz), 1.04 (6 H, t, *J=*7.0 Hz); ^13^C NMR (298 K, 100 MHz, CDCl_3_): *δ*_C_=153.6, 141.1, 131.1, 130.1, 113.5, 83.1, 55.5, 46.0, 12.0; (HRMS+) *m*/*z* 306.0353 [*M*+H]^+^ (C_11_H_17_NOI requires 306.0355).

***N***,***N*** **‐Diethyl‐2‐methoxy‐5‐((trimethylsilyl)ethynyl)aniline, 14**: Compound **13** (1.58 g, 5.18 mmol) was dissolved in dry THF (10 mL) before addition of Pd(PPh_3_)_2_Cl_2_ (364 mg, 0.5 mmol), TMS‐acetylene (3.80 mL, 25.9 mmol) and pyrrolidine (2.20 mL, 25.9 mmol). The reaction was stirred at 50 °C for 24 h under argon. After LC–MS analysis indicated reaction completion, solvent was removed and the residual dark‐brown oil was dissolved in CH_2_Cl_2_/water (40 mL, 1:1). The aqueous layer was extracted with CH_2_Cl_2_ (3×10 mL). The combined organic layers were dried over Na_2_SO_4_, filtered and concentrated to dryness to give a crude dark‐brown oil. This was purified by column chromatography (SiO_2_, CH_2_Cl_2_ 100 %) to give a dark‐brown oil (1.41 g, quant.); *R*_f_=0.65 (SiO_2_, 2 % MeOH in CH_2_Cl_2_); ^1^H NMR (700 MHz, CDCl_3_): *δ*_H_=7.12 (1 H, d, *J=*8.0 Hz), 7.03 (1 H, s), 6.75 (1 H, d, *J=*8.0 Hz), 3.85 (3 H, s), 3.14 (4 H, q, *J=*7.0 Hz), 1.01 (6 H, t, *J=*7.0 Hz), 0.24 (9 H, s); ^13^C NMR (176 MHz, CDCl_3_): *δ*_C_=154.5, 139.1, 127.2, 125.3, 115.1, 111.2, 105.9, 91.9, 55.6, 46.1, 12.1, 0.3.; (HRMS+) *m*/*z* 276.1770 [*M*+H]^+^ (C_16_H_26_NOSi requires 276.1784).

***N***,***N*** **‐Diethyl‐5‐ethynyl‐2‐methoxyaniline, 15**: Compound **14** (1.41 g, 5.13 mmol) was dissolved in dry THF (10 mL), then NEt_3_⋅3 HF (5.0 mL, 26.7 mmol) was added. The reaction was stirred at 30 °C for 24 h under argon. After this time, solvent was removed and the residual oil was dissolved in CH_2_Cl_2_/water (40 mL, 1:1). The aqueous layer was extracted with CH_2_Cl_2_ (3×20 mL) and the combined organic layers were dried over Na_2_SO_4_, filtered and concentrated to dryness to yield a light‐orange oil that was purified by column chromatography (SiO_2_, hexane to 2 % EtOAc in hexane) to afford a light‐brown oil (0.73 g, 75 %); *R*_f_=0.65 (SiO_2_, 2 % MeOH in CH_2_Cl_2_); ^1^H NMR (700 MHz, CDCl_3_): *δ*_H_=7.14 (1 H, dd, *J=*8.0 Hz, *J=*2.0 Hz), 7.06 (1 H, d, *J=*2.0 Hz), 6.78 (1 H, d, *J=*8.0 Hz), 3.86 (3 H, s), 3.15 (4 H, q, *J=*7.0 Hz), 2.98 (1 H, s), 1.02 (6 H, t, *J=*7.0 Hz); ^13^C NMR (176 MHz, CDCl_3_): *δ*_C_=154.6, 139.2, 127.1, 125.3, 113.9, 111.3, 84.4, 75.3, 55.7, 46.1, 12.1; (HRMS+) *m*/*z* 204.1401 [*M*+H]^+^ (C_13_H_18_NO requires 204.1388).

**Ethyl‐(4‐((3‐(diethylamino)‐4‐methoxyphenyl)ethynyl)‐6‐(hydroxy methyl)pyridin‐2‐yl)(methyl)phosphinate, 16**: To a solution of compound **15** (55 mg, 0.27 mmol) and compound **9** (73 mg, 0.25 mmol) in anhydrous THF (2 mL) under argon was added pyrrolidine (250 μL) and Pd(dppf)Cl_2_⋅CH_2_Cl_2_ (20 mg, 0.03 mmol). The reaction mixture was subsequently heated at 50 °C for 24 h. After this time, solvent was removed under reduced pressure and the residue was dissolved in CH_2_Cl_2_/water (20 mL, 1:1). The aqueous layer was extracted with CH_2_Cl_2_ (3×10 mL) and the combined organic layers were dried over Na_2_SO_4_, filtered and concentrated to dryness to yield a crude brown residue that was purified by reverse‐phase HPLC (CH_3_CN/water) to afford a pale‐yellow oil (42 mg, 41 %). ^1^H NMR (600 MHz, CDCl_3_): *δ*_H_=8.03 (1 H, d, *J=*6.0 Hz), 7.49 (1 H, s), 7.18 (1 H, d, *J=*8.0), 7.09 (1 H, s), 6.82 (1 H, d, *J=*8.0 Hz), 4.80 (2 H, s), 4.14–4.06 (1 H, m), 3.87 (3 H, s), 3.92–3.82 (1 H, m), 3.16 (4 H, q, *J=*7.0 Hz), 1.77 (3 H, d, *J=*15.0 Hz), 1.26 (3 H, t, *J=*7.0 Hz), 1.03 (6 H, t, *J=*7.0 Hz); ^13^C NMR (151 MHz, CDCl_3_): *δ*_C_=160.8 (d, *J=*20.0 Hz), 155.2, 153.2 (d, *J=*160.0 Hz), 139.5, 133.3 (d, *J=*11.0 Hz), 128.3 (d, *J=*22.0 Hz), 127.2, 125.0, 124.1 (d, *J=*3.0 Hz), 113.5, 111.5, 97.0, 84.9, 64.2, 61.3 (d, *J=*6.0 Hz), 55.7, 46.0, 16.5 (d, *J=*6.0 Hz), 13.6 (d, *J=*105.0 Hz), 12.0; ^31^P{^1^H} NMR (243 MHz, CDCl_3_): *δ*_P_=39.44; UPLC (CH_3_CN/H_2_O, 0.1 % FA) *t*_R_=1.22 min; (HRMS+) *m*/*z* 417.1954 [*M*+H]^+^ (C_22_H_30_N_2_O_4_P requires 417.1943).

**(4‐((3‐(Diethylamino)‐4‐methoxyphenyl)ethynyl)‐6‐(ethoxy(methyl)phosphoryl)pyridin‐2‐yl)methyl methanesulfonate, 17**: The alcohol **16** (22 mg, 0.05 mmol), methanesulfonic anhydride (22 mg, 0.11 mmol) and triethylamine (25 μL, 0.18 mmol) were combined under argon in anhydrous THF (1.5 mL) under argon. The solution was subsequently stirred at room temperature for 3 h. After this time, solvent was removed under reduced pressure to yield a red oil which was dissolved in CH_2_Cl_2_ (10 mL). The organic layer was washed with water (3×10 mL), dried over Na_2_SO_4_ and evaporated to dryness to afford the mesylate **17** (20 mg, 77 %). The compound was used directly in the next step without further purification; ^1^H NMR (298 K, 400 MHz, CDCl_3_): *δ*_H_=8.07 (1 H, d, *J=*6.0 Hz), 7.58–7.54 (3 H, m), 6.89 (1 H, d, *J=*7.0 Hz), 4.86 (2 H, s), 4.17–4.11 (1 H, m), 4.00–3.85 (3 H, m), 3.24 (4 H, br. s), 3.19 (3 H, s), 1.81 (3 H, d, *J=*15.0 Hz), 1.30 (3 H, t, *J=*7.0 Hz), 1.09 (6 H, t, *J=*7.0 Hz), UPLC (CH_3_CN/H_2_O, 0.1 % FA) *t*_R_=1.39 min.

***tert*** **‐Butyl 4,7‐bis((6‐(ethoxy(methyl)phosphoryl)pyridin‐2‐yl)methyl)‐1,4,7‐triazacyclonane‐1‐carboxylate, 20**: 1‐*tert‐*Butoxycarbonyl‐1,4,7‐triazacyclononane **19** (60 mg, 0.26 mmol) and freshly prepared compound **18** (200 mg, 0.65 mmol) were dissolved in dry CH_3_CN (5 mL) and K_2_CO_3_ (152 mg, 1.12 mmol) was added. The reaction mixture was stirred at 60 °C for 12 h under argon at which point the mixture was allowed to cool to room temperature. The excess potassium salts were removed by filtration and the filtrate was concentrated under reduced pressure. The residue was subsequently dissolved in CH_2_Cl_2_/water (40 mL, 1:1). The aqueous layer was extracted with CH_2_Cl_2_ (3×20 mL) and the combined organic layers were dried over Na_2_SO_4_, filtered and evaporated to dryness to yield an orange oil. The residual oil was purified by reverse‐phase HPLC (CH_3_CN/water with 0.1 % formic acid) to yield compound **20** (88 mg, 54 %) as an orange oil; ^1^H NMR (298 K, 700 MHz, CDCl_3_): *δ*_H_=7.91–7.86 (2 H, dd, *J=*8.0 Hz, *J=*6.0 Hz), 7.77–7.70 (2 H, m), 7.61 (1 H, d, *J=*8.0 Hz), 7.54 (1 H, d, *J=*8.0 Hz), 4.09–4.02 (2 H, m), 3.92 (4 H, s), 3.85–3.79 (2 H, m), 3.43–2.54 (12 H, m), 1.73 (3 H, d, *J=*15.0 Hz), 1.72 (3 H, d, *J=*15.0 Hz), 1.43 (9 H, s), 1.22 (2×3 H, 2×t, *J=*7.0 Hz); ^13^C NMR (298 K, 100 MHz, CDCl_3_): *δ*_C_=165.0, 159.4 (d, *J=*20.0 Hz), 158.9 (d, *J=*20.0 Hz), 155.4, 153.8 (d, *J=*150.0 Hz), 136.7 (d, *J=*9.0 Hz) 126.3 (d, *J=*3.0 Hz), 126.1 (d, *J=*3.0 Hz), 126.0, 80.0, 62.4, 61.0 (d, *J=*4.0 Hz), 60.9 (d, *J=*4.0 Hz), 55.1, 54.3, 53.9, 53.4, 49.6, 48.9, 28.5, 16.4 (d, *J=*6.0 Hz), 13.4 (d, *J=*100.0 Hz); ^31^P{^1^H} NMR (298 K, 162 MHz, CDCl_3_): *δ*_P_=40.18, 40.01; UPLC (CH_3_CN/H_2_O, 0.1 % FA) *t*_R_=1.47 min; (HRMS+) *m*/*z* 624.3088 [*M*+H]^+^ (C_29_H_48_N_5_O_6_P_2_ requires 624.3080).

**Diethyl‐(((1,4,7‐triazacyclononane‐1,4‐diyl)bis(methylene))bis(pyridine‐6,2‐diyl))bis(methylphosphinate), 21**: The mono‐carbamate **20** (88 mg, 140 μmol) was dissolved in dry CH_2_Cl_2_ (3 mL) before the addition of trifluoroacetic acid (0.6 mL). The solution was stirred at room temperature for 30 min after which the solvent was removed under reduced pressure. The residue was treated with CH_2_Cl_2_ (2 mL) and the solvent removed under reduced pressure again; this process was repeated several times. The crude mixture was purified by reversed‐phase HPLC (CH_3_CN/water with 0.1 % formic acid) to afford a dark‐brown oil (73.1 mg, quant.); ^1^H NMR (298 K, 400 MHz, CDCl_3_): *δ*_H_=7.86–7.78 (4 H, m), 7.44 (2 H, d, *J=*8.0 Hz), 4.19 (4 H, s), 4.16–4.08 (2 H, m), 3.94–3.88 (2 H, m), 3.40–3.04 (12 H, m), 1.74 (6 H, d, *J=*15.0 Hz), 1.28 (6 H, t, *J=*7.0 Hz); ^13^C NMR (298 K, 100 MHz, CDCl_3_): *δ*_C_=158.0 (d, *J=*20 Hz), 153.8 (d, *J=*150 Hz), 137.3 (d, *J=*9.0 Hz) 126.0 (d, *J=*20.0 Hz), 125.8 (d, *J=*2.0 Hz), 61.6 (d, *J=*6.0 Hz), 60.0, 51.5, 48.9, 44.5, 16.4 (d, *J=*6.0 Hz), 13.7 (d, *J=*100 Hz); ^31^P{^1^H} NMR (298 K, 162 MHz, CDCl_3_): *δ*_P_=40.12; UPLC (CH_3_CN/H_2_O, 0.1 % FA) *t*_R_=1.09 min; (HRMS+) *m*/*z* 524.2557 [*M*+H]^+^ (C_24_H_40_N_5_O_4_P_2_ requires 524.2556).

**Diethyl‐(((7‐((4‐((3‐(diethylamino)‐4‐methoxyphenyl)ethynyl)‐6‐(ethoxy(methyl)phosphoryl)pyridin‐2‐yl)methyl)‐1,4,7‐triazacyclo nonane‐1,4‐diyl)bis(methylene))bis(pyridine‐6,2‐diyl))bis(methyl phosphinate), 22**: The disubstituted triazacyclononane **20** (8.5 mg, 16.2 μmol), mesylate **17** (16.0 mg, 32.4 μmol) and K_2_CO_3_ (5 mg, 35.4 μmol) were combined in anhydrous CH_3_CN (1.0 mL) and stirred at 60 °C for 12 h under argon. After this time, the solvent was removed under reduced pressure and the residue was dissolved in CH_2_Cl_2_/water (40 mL). The aqueous layer was extracted with CH_2_Cl_2_ (3×20 mL). The combined organic layers were dried over Na_2_SO_4_, filtered and concentrated to dryness. The crude product was purified by reversed‐phase HPLC (CH_3_CN/25 mm ammonium bicarbonate buffer) to afford a yellow oil (7.4 mg, 50 %); ^1^H NMR (298 K, 400 MHz, CDCl_3_): *δ*_H_=8.04 (1 H, dd, *J=*6.0 Hz, *J=*2.0 Hz), 7.93 (2 H, app. t, *J=*6.0 Hz), 7.83–7.78 (2 H, m), 7.69–7.65 (3 H, m), 7.21 (1 H, br. d, *J*=8.0 Hz), 7.12 (1 H, d, *J=*2.0 Hz), 6.87 (1 H, d, *J=*8.0 Hz), 4.18–3.76 (15 H, m), 3.20 (4 H, q, *J=*7.0 Hz) 2.93–2.89 (12 H, m), 1.79 (3 H, d, *J=*15.0 Hz), 1.78 (6 H, d, *J=*15.0 Hz), 1.29 (6 H, t, *J=*7.0 Hz), 1.27 (3 H, t, *J=*7.0 Hz), 1.07 (6 H, t, *J=*7.0 Hz);^13^C NMR (101 MHz, CDCl_3_): *δ*_C_=161.5 (2×d, *J=*12.0 Hz), 155.0, 153.6 (2×d, *J=*160.0 Hz), 139.4, 136.4 (d, *J=*9.0 Hz), 133.3 (d, *J=*16.0 Hz), 127.8 (d, *J=*20.0 Hz), 127.0, 126.4 (d, *J=*4.0 Hz), 125.9, 125.6 (d, *J=*20.0 Hz), 124.8, 113.4, 111.3, 96.4, 85.1, 64.4 (2×s), 60.9 (2×d, *J=*6.0 Hz), 55.6, 45.9, 16.5 (2×d, *J=*6.0 Hz), 13.4 (2×d, *J=*105.0 Hz), 11.9; ^31^P{^1^H} NMR (298 K, 162 MHz, CDCl_3_): *δ*_P_=40.24 (2P), 39.99 (1P); UPLC (CH_3_CN/H_2_O, 0.1 % FA) *t*_R_=1.35 min; (HRMS+) *m*/*z* 922.4320 [*M*+H]^+^ (C_46_H_67_N_7_O_7_P_3_ requires 922.4315);

**[Eu*L*** ^***3***^ **]**: The ligand **22** (2 mg, 2 μmol) was dissolved in a mixture of CH_3_OH/H_2_O (1:1, 2 mL total) and the pH was adjusted to 12 using aqueous NaOH solution (0.1 m). The solution was stirred at 60 °C for 14 h. After cooling and adjustment to pH 7 using dilute hydrochloric acid (0.1 m), EuCl_3_⋅6 H_2_O (3 mg, 8 μmol) was added and the reaction mixture was stirred at 60 °C for 15 h. The reaction mixture was purified by reverse phase HPLC (CH_3_CN/water) to yield a white powder after freeze drying (2 mg, 93 %); (HRMS+) *m*/*z* 986.2327 [*M*+H]^+^ (C_40_H_52_N_7_O_7_^151^EuP_3_ requires 986.2340); UPLC (CH_3_CN/H_2_O, 0.1 % TFA) *t*_R_=1.54 min; τ_H2O_=(ms)=0.50 (pH 9), 0.53 (pH 8), 0.74 (pH 7), 1.06 (pH 6), 1.15 (pH 5), 1.16 (pH 4); *τ*_MeOH_=0.89 ms; *q*=0; *λ*_exc_=331 nm; *ϵ*_331_  n_m_=12 000 m ^−1^⋅cm^−1^; *Φ*_H2O_=0.01 % (pH 9), 6.3 % (pH 4).

## Conflict of interest

The authors declare no conflict of interest.

## Supporting information

As a service to our authors and readers, this journal provides supporting information supplied by the authors. Such materials are peer reviewed and may be re‐organized for online delivery, but are not copy‐edited or typeset. Technical support issues arising from supporting information (other than missing files) should be addressed to the authors.

Supplementary
