# Supplementary material for: Synthesis and Evaluation of Europium Complexes that Switch on Luminescence in Lysosomes of Living Cells
Source: Chemistry. 2020 Dec 15;27(2):766–77. doi: 10.1002/chem.202003992 (PMC7839496; doi:10.1002/chem.202003992)
Supplement: Supplementary file 1 — Supplementary [file CHEM-27-766-s001.pdf]

Chemistry–A European Journal

Supporting Information

Synthesis and Evaluation of Europium Complexes that Switch on Luminescence in Lysosomes of Living Cells

Matthieu Starck,*[a] Jack D. Fradgley,[a] Robert Pal,[a] Jurriaan M. Zwier,[b] Laurent Lamarque,[b] and David Parker*[a]

General Experimental 2
Analytical Methods 2
Optical measurements 3
Synthesis of [Eu*L*[2]] 5
Synthesis of [Eu*L*[4]] 6
Photophysical Spectral Data 8
Cell Imaging 12
NMR Spectra of Intermediates and Ligands 14
GC and HPLC Traces 56
References 61

General Experimental

All reagents were purchased and used as received. Analytical solvents were purchased from Fisher Scientific and Sigma Aldrich and were HPLC grade. Anhydrous solvents were freshly distilled over the appropriate drying agent and stored under argon in a septum-capped bottle. Water was purified by the 'Purite$_{STILL}$plus' system, with conductivity of ≤ 0.04 μS cm^{-1}. Air sensitive reactions were carried out under an atmosphere of argon using Schlenk-line techniques.

Analytical Methods

Thin Layer Chromatography was carried out on neutral aluminium silica plates (Merck 5554) or neutral aluminium oxide plates (Merck 5550) and visualised under UV irradiation (254/365 nm). Preparative column chromatography was performed using silica gel (Merck Silica Gel 60, 230-400 mesh) or neutral aluminium oxide (Merck 90, 70-320 mesh).

Proton (^{1}H) and carbon (^{13}C) NMR spectra were recorded on a Bruker Avance III-HD-400 (400.07 MHz for ^{1}H, 100.60 MHz for ^{13}C and 161.95 MHz for ^{31}P), Bruker Neo-400 (400.20 MHz for ^{1}H, 100.63 MHz for ^{13}C and 162.00 MHz for ^{31}P), Varian DD2-500 (499.53 MHz for ^{1}H, 125.61 MHz for ^{13}C and 202.21 MHz for ^{31}P), Varian VNMRS-600 (599.42 MHz for ^{1}H, 150.72 MHz for ^{13}C and 242.65 MHz for ^{31}P), or Varian VNMRS-700 (699.73 MHz for ^{1}H, 175.95 MHz for ^{13}C and 283.25 MHz for ^{31}P) spectrometers. Spectra were recorded in commercially available deuterated solvents. ^{1}H and ^{13}C chemical shift values are quoted in ppm relative to tetramethylsilane and coupling constants are given in Hz. The operating temperature of the spectrometers (295 K) was measured using an internal calibration solution of ethylene glycol.

Melting points were recorded using a Gallenkamp (Sanyo) apparatus and are uncorrected.

Reverse phase preparative HPLC purification was undertaken at 295 K using a Shimadzu system consisting of a Degassing Unit (DGU-20A$_{5R}$), a Prominence Preparative Liquid Chromatograph (LC-20AP), a Prominence UV/Vis Detector (SPD-20A) and a Communications Bus Module (CBM-20A). An XBridge C18 OBD 19 x 100 mm, i.d. 5 μM column was used (flow rate of 17 mL/min (prep). The elutant was ammonium bicarbonate buffer (25 mM, pH 7.55) / acetonitrile (isocratic 10% acetonitrile in buffer (3 min), linear gradient to 100% acetonitrile (10 min), isocratic 100% acetonitrile (5 min).

Mass spectrometry analysis was performed on a QToF Premier equipped with an Acquity UPLC (Waters Corp.). The reverse phase gradient separation was undertaken using an Acquity UPLC BEH C18 column 1.7 μm (2.1 mm x 100 mm) (Waters Corp.). The solvent gradient ran from 100% water containing 0.1% formic acid to 100% acetonitrile over a period of 5 minutes (0.4 mL/min). Positive ions from the electrospray ion source were recorded as a full MS spectrum, and the appropriate precursor ion was mass selected by the quadrupole, with an isolation window that permitted

transmission of the full isotopic envelope. A reference spray provided a 'lock mass' that allowed a calibration correction to be performed for the accurate mass determination of the MS data.

Optical measurements

Absorption spectroscopy. UV/Vis absorption measurements were recorded using a Perkin-Elmer Lambda 900 absorption spectrophotometer, using matched quartz cells.

Luminescence. Emission spectra were measured using a Horiba-Jobin Yvon Fluorolog-3® spectrofluorimeter. The steady-state luminescence was excited by unpolarised light from a 450W xenon CW lamp and detected at an angle of 90° for diluted solution measurements (10 mm quartz cell) by a red-sensitive Hamamatsu R928 photomultiplier tube. Spectra were reference corrected for both the excitation source light intensity variation (lamp and grating) and the emission spectral response (detector and grating). Phosphorescence lifetimes (> 30 μs) were obtained by pulsed excitation using a FL-1040 UP Xenon Lamp. Luminescence decay curves were fitted by least-squares analysis using Origin®. Photoluminescence quantum yields were measured in diluted aqueous solution with an absorbance lower than 0.1, using the following equation (1):

$$\frac{Q_\mathrm{x}}{Q_\mathrm{r}} = \left[\frac{A_\mathrm{r}(\lambda)}{A_\mathrm{x}(\lambda)}\right]\left[\frac{n_\mathrm{x}^2}{n_\mathrm{r}^2}\right]\left[\frac{D_\mathrm{x}}{D_\mathrm{r}}\right]\left[\frac{I_\mathrm{lamp}(\lambda)_\mathrm{x}}{I_\mathrm{lamp}(\lambda)_\mathrm{r}}\right] \qquad (1)$$

where *A* is the absorbance at the excitation wavelength (λ), η is the refractive index, *D* is the integrated luminescence intensity, I_{lamp} is the energy intensity contribution of the excitation lamp at the excitation wavelength (λ). "r" and "x" stand for reference and sample. Here, the reference is $[Ru(bipy)_3]Cl_2$ in non-degassed water (Φ = 2.8%).[1] Estimated errors are ±15%. Spectrometric titrations were performed according to methods described in the literature.[2]

Confocal Microscopy. Cell microscopy imaging of europium complexes in cells was undertaken using a custom built epifluorescence microscope (modified Zeiss Axiovert 200M), using a Zeiss APOCHROMAT 63x/1.40 NA objective, combined with a low voltage 365 nm pulsed UV LED focused and collimated excitation source (1.2 W). For rapid spectral acquisition, the microscope was equipped at the X1 port with a Peltier cooled 2D-CCD detector (Ocean Optics), used in an inverse 100 Hz time gated sequence. The spectrum was recorded from 400-800 nm with a resolution of 0.24 nm and the final spectrum was acquired using an averaged 10,000 scan duty cycle.

Probe lifetimes were measured on the same microscope platform using a novel cooled PMT detector (Hamamatsu H7155), interchangeable on the X1 port. Both the control and detection algorithm were written in LabView2011. Time gated images were recorded using a high resolution cooled EO-1312M CCD camera (Thor labs). All duty cycle and gating sequences were established and controlled using ' in house' LabView software.[3]

High resolution LSCM images were recorded on a modified Leica SP5 II microscope, equipped with a new SIM technique called PhMoNa.[4] In order to achieve excitation with maximal probe emission, the microscope was coupled by an optical fibre to a Coherent CW laser (Nd:YAG, 355 nm), operating at 8 mW power. A He/Ne or Ar ion laser was used when commercially available organelle-specific stains (e.g. LysoTrackerGreen™) were used to corroborate cellular compartmentalization.

The microscope was equipped with a triple channel imaging detector, comprising two conventional PMT systems and a HyD hybrid avalanche photodiode detector. The latter part of the detection system, when operated in the BrightRed mode, is capable of improving imaging sensitivity above 550 nm by 25%, reducing signal to noise by a factor of 5. The pinhole was always determined by the Airy disc size, calculated from the objective in use (HCX PL APO 63x/1.40 NA αBlue), using the lowest excitation wavelength (355 nm). Scanning speed was adjusted to 100 Hz in a unidirectional mode, to ensure both sufficient light exposure and enough time to collect the emitted light from the lanthanide based optical probes (2048 x 2048 frame size, a pixel size of 62 x 62 nm and depth of 789 nm). Spectral imaging on this Leica system is possible with the *xyλ*-scan function, using the smallest allowed spectral band-pass (5nm) and step-size (3nm) settings. However, much improved spectral imaging in cells was achieved using a custom built and Peltier cooled CCD detector (Ocean Optics, HR2000plus) synchronized to the X1 port.

Synthesis of [EuL^2]

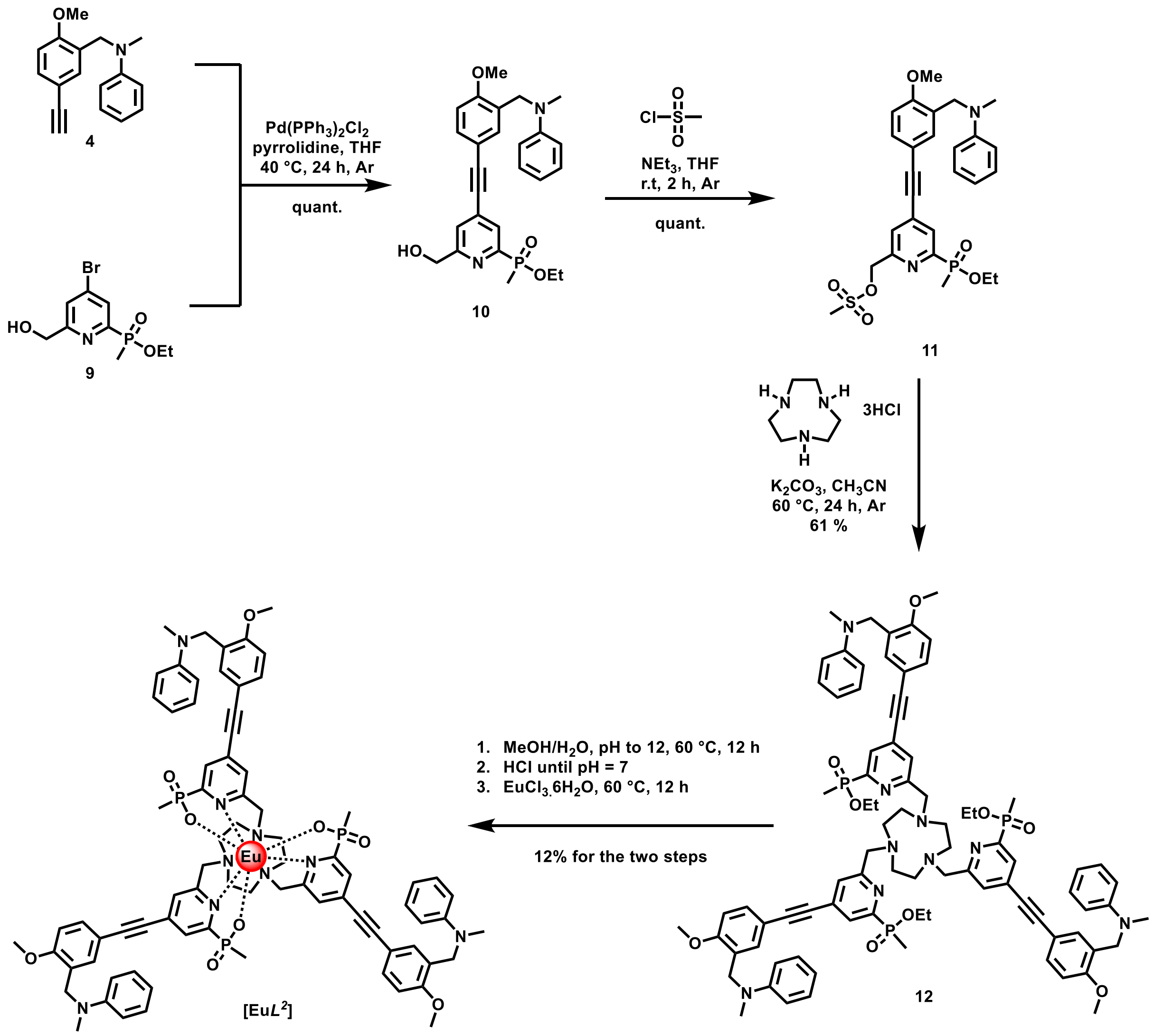

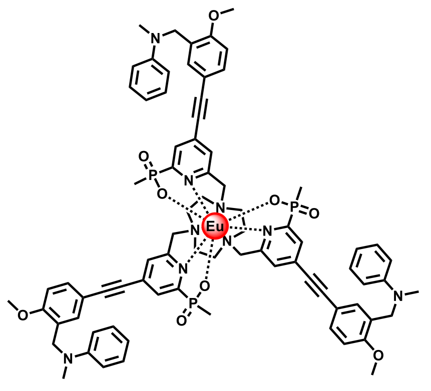

Europium complex of (((1,4,7-triazacyclonane-1,4,7-triyl)tris(methylene))tris(4-((4-methoxy-3-((methyl(phenyl) amino)methyl)phenyl)ethynyl)pyridine-6,2-diyl))tris(methyl phosphinate), [Eu*L*[2]]. Compound **12** (21 mg, 20.4 μmol) was dissolved in a solution of CH_3OH (1 mL), and aqueous sodium hydroxide solution (1 mL, 0.1 M) was added to reach pH = 12. The solution was stirred at 60 °C overnight. After LC/MS analysis indicated complete ester hydrolysis, the pH was adjusted to 7 with addition of a 1 M HCl solution. $EuCl_3.6H_2O$ (8.5 mg, 23 μmol) was added and the solution was stirred at 60 °C for 12 h. After this time, the solution was evaporated to yield the crude Eu(III) complex as a yellow solid (bright red under long wave U.V. light). The complex was purified by reverse phase HPLC (CH_3CN/25 mM ammonium bicarbonate buffer) to give a white powder (2.6 mg, 12%) after freeze- drying; **UPLC** (CH_3CN/Ammonium bicarbonate buffer) t_R = 6.57 min; **(HRMS+)** *m/z*

1532.462 [M+H]$^+$ ($C_{78}H_{82}N_9O_9{}^{151}EuP_3$ requires 1532.465); $\boldsymbol{\tau}_{\mathbf{H_2O}}^{\mathbf{pH\,7}}$ = 0.65 ms; $\boldsymbol{\tau}_{\mathbf{D_2O}}$ = 0.99 ms; $\boldsymbol{\tau}_{\boldsymbol{MeOH}}$ = 1.07 ms; $\boldsymbol{q}$ = 0; $\boldsymbol{\lambda}_{\mathbf{max}}$ = 324 nm; $\boldsymbol{\varepsilon}_{\mathbf{324\,nm}}^{\mathbf{pH\,7}}$ = 21000 M^{-1}.cm^{-1}; $\boldsymbol{\phi}_{\mathbf{H_2O}}^{\mathbf{pH\,7}}$ = 12%, $\boldsymbol{\phi}_{\mathbf{MeOH}}$ = 46%.

Synthesis of [Eu*L*^{4}]

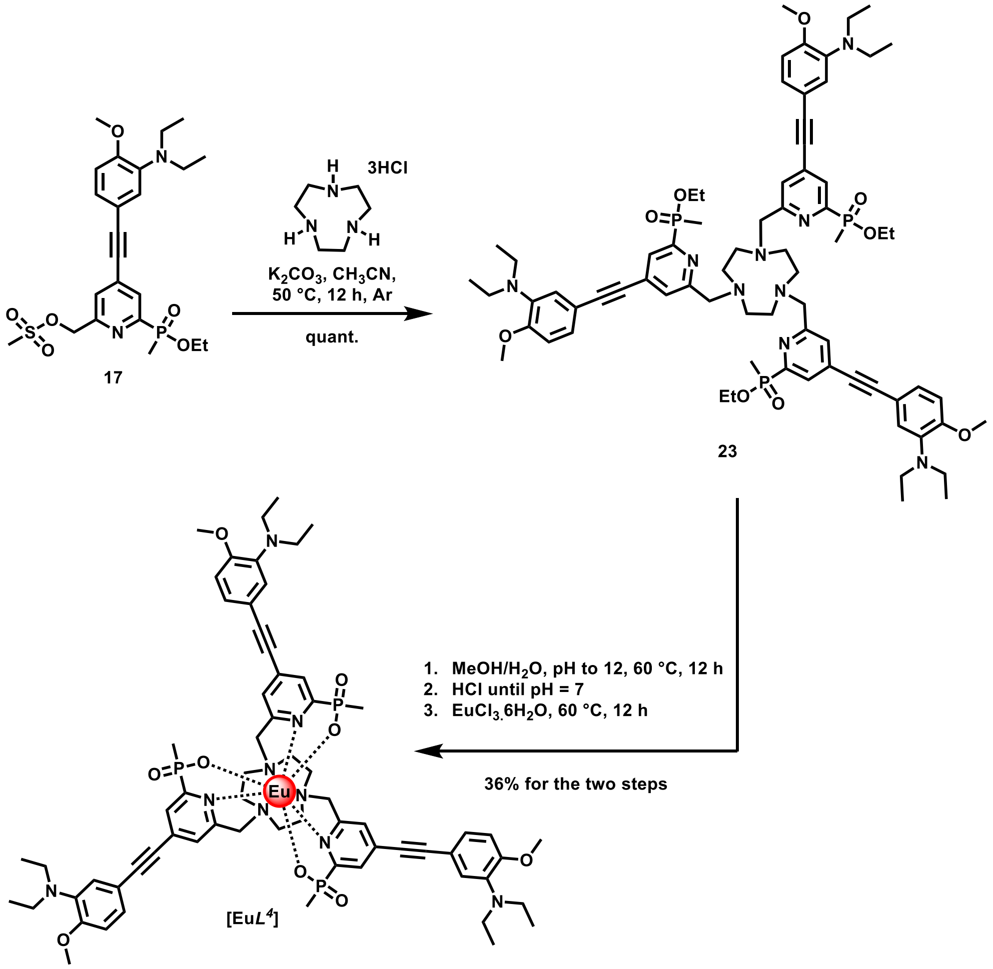

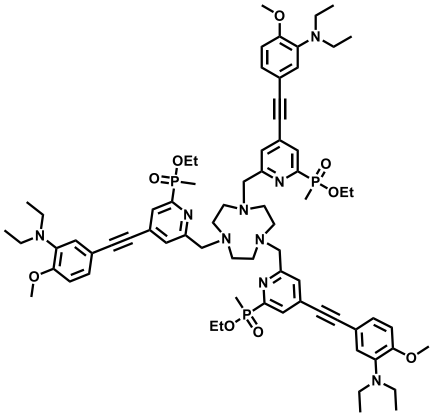

Triethyl (((1,4,7-triazacyclononane-1,4,7-triyl)tris(methylene)) tris(4-((3-(diethylamino)-4-methoxyphenyl)ethynyl)pyridine-6,2-diyl))tris(methylphos-phinate), 23. 1,4,7-Triazacyclo nonane trihydrochloride (6.7 mg, 28.5 µmol) and the mesylate **17** (49.3 mg, 100.0 µmol) were dissolved in anhydrous CH_3CN (1 mL) and K_2CO_3 (12.8 mg, 90.0 µmol) was added. The mixture was stirred at 50 °C for 12 h under argon. The excess potassium salts were removed by filtration and the filtrate was concentrated to dryness. The residue was dissolved in CH_2Cl_2/water (40 mL) and the aqueous layer was extracted with CH_2Cl_2 (3 × 20 mL). The combined organic layers were dried over Na_2SO_4, filtered and concentrated to dryness to yield an orange oil (36.8 mg, quant.). The compound was used directly in the next step without further purification; **^{1}H NMR** (298 K, 400 MHz, $CDCl_3$) δ_H 8.03 (3H,

dd, J = 6.0 Hz, J = 2.0 Hz), 7.70 (3H, t, J = 2.0 Hz), 7.20 (3H, dd, J = 8.0 Hz, J = 2.0 Hz), 7.30 (3H, d, J = 2.0 Hz), 6.84 (3H, d, J = 8.0 Hz), 4.13 – 4.02 (3H, m), 3.98 – 3.81 (18H, m), 3.17 (12H, q, J = 7.0 Hz), 2.95 (12H, s), 1.78 (9H, d, J = 15.0 Hz), 1.26 (9H, t, J = 7.0 Hz), 1.04 (18H, t, J = 7.0 Hz); **^{13}C NMR** (298 K, 100 MHz, $CDCl_3$) δ_C 161.6 (d, J = 20 Hz), 155.0, 153.7 (d, J = 150.0 Hz), 139.4, 133.1, 132.6 (d, J = 12.0 Hz), 127.7 (d, J = 24 Hz), 127.0, 126.5, 124.8, 113.6, 111.4, 96.3, 85.1, 63.8, 61.0 (d, J = 7.0 Hz), 55.7, 55.6, 45.9, 16.5 (d, J = 7.0 Hz), 13.3 (d, J = 115 Hz), 12.0; **^{31}P{^{1}H} NMR** (298 K, 162 MHz, $CDCl_3$) δ_P 40.05; **LC/MS** (CH_3CN/H_2O, 0.1% FA) t_R = 1.38 min; **(HRMS+)** *m/z* 1324.662 $[M+H]^+$ ($C_{72}H_{97}N_9O_9P_3$ requires 1324.662).

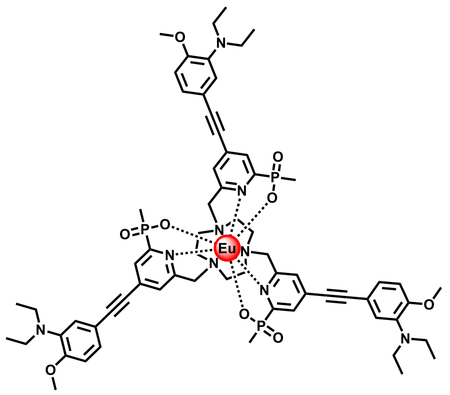

Europium complex of (((1,4,7-triazacyclononane-1,4,7-triyl)tris(methylene))tris(4-((3-(diethylamino)-4-methoxy phenyl)ethynyl)pyridine-6,2-diyl))tris(methylphosphinate), [EuL^4]. The ligand **23** (8 mg, 6 μmol) was dissolved in a mixture of CH_3OH/H_2O (1:1, 2 mL total) and the pH was adjusted to 12 using aqueous NaOH solution (1 M). The solution was stirred at 60 °C for 4 h. After cooling and adjustment of the pH to 7 using aqueous HCl solution (1 M), $EuCl_3.6H_2O$ (3 mg, 8 μmol) was added and the reaction mixture was stirred at 60 °C for 19 h. The reaction mixture was purified by reverse phase HPLC (CH_3CN/water) to yield **[Eu*L^4*]** (4 mg, 48%) as a white solid; **(HRMS+)** *m/z* 1390.467 $[M+H]^+$ ($C_{66}H_{82}N_9O_9{}^{151}EuP_3$ requires 1390.467); **UPLC** (CH_3CN/H_2O, 0.1% FA) t_R = 1.15 min; $\boldsymbol{\tau_{H_2O}}$ (ms) = 0.24 (pH 9), 0.25 (pH 8), 0.32 (pH 7), 0.59 (pH 6), 0.83 (pH 5), 0.84 (pH 4); $\boldsymbol{\tau_{MeOH}}$ = 0.89 ms; $\boldsymbol{q}$ = 0; $\boldsymbol{\lambda_{exc}}$ = 331 nm; $\boldsymbol{\varepsilon_{331\ nm}}$ = 46000 (pH 8) – 60000 (pH 4) $M^{-1}.cm^{-1}$; $\boldsymbol{\phi_{H_2O}^{pH\ 8}}$ = 0.1%, $\boldsymbol{\phi_{H_2O}^{pH\ 4}}$ = 17%.

Photophysical Spectral Data

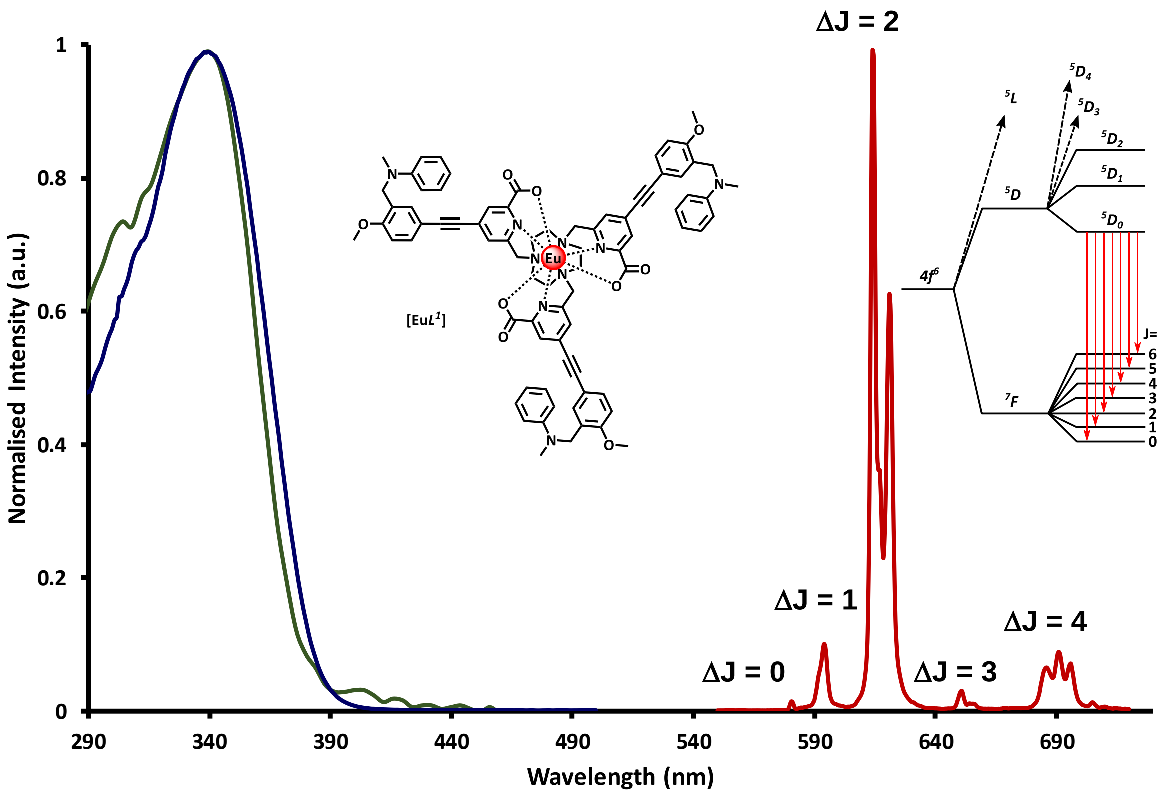

Figure S1. Absorption, excitation and metal-based emission spectra for [Eu***L***[1]], (c = 15 μM, λ_{exc} 331 nm, 295 K, 0.1 M NaCl), showing the highest energy transitions from the $^5D_0$ excited state.

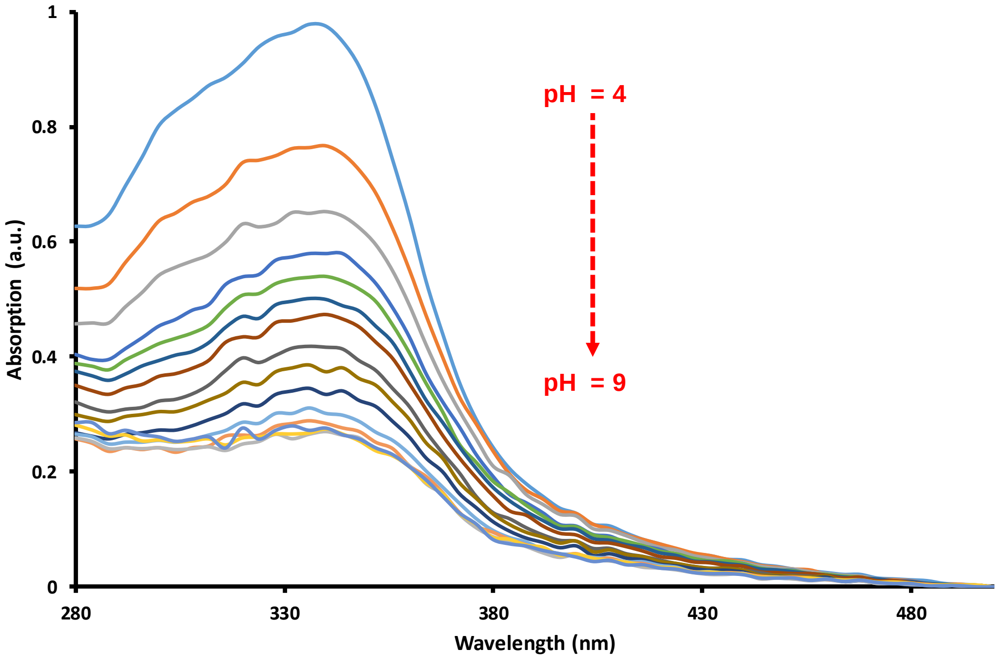

Figure S2. Variation of the absorption spectrum of [Eu***L***[1]] with pH revealing the absence of isosbestic points (c = 15 μM, 295 K, 0.1 M NaCl).

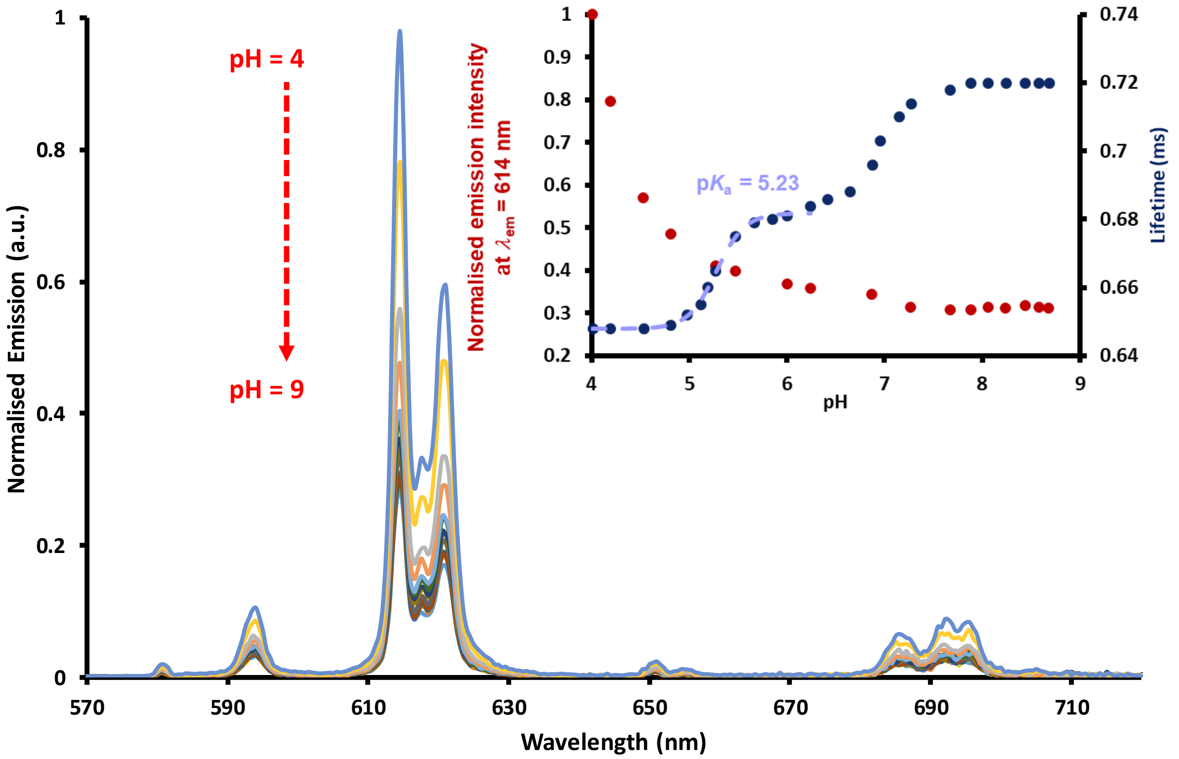

Figure S3. Variation of the europium emission spectrum and lifetime with pH for [Eu*L*¹] (c = 15 μM, λ_{exc} 340 nm, 295 K, 0.1 M NaCl) showing the fit (line) to experimental data. Similar plots were obtained for excitation at 355 nm.

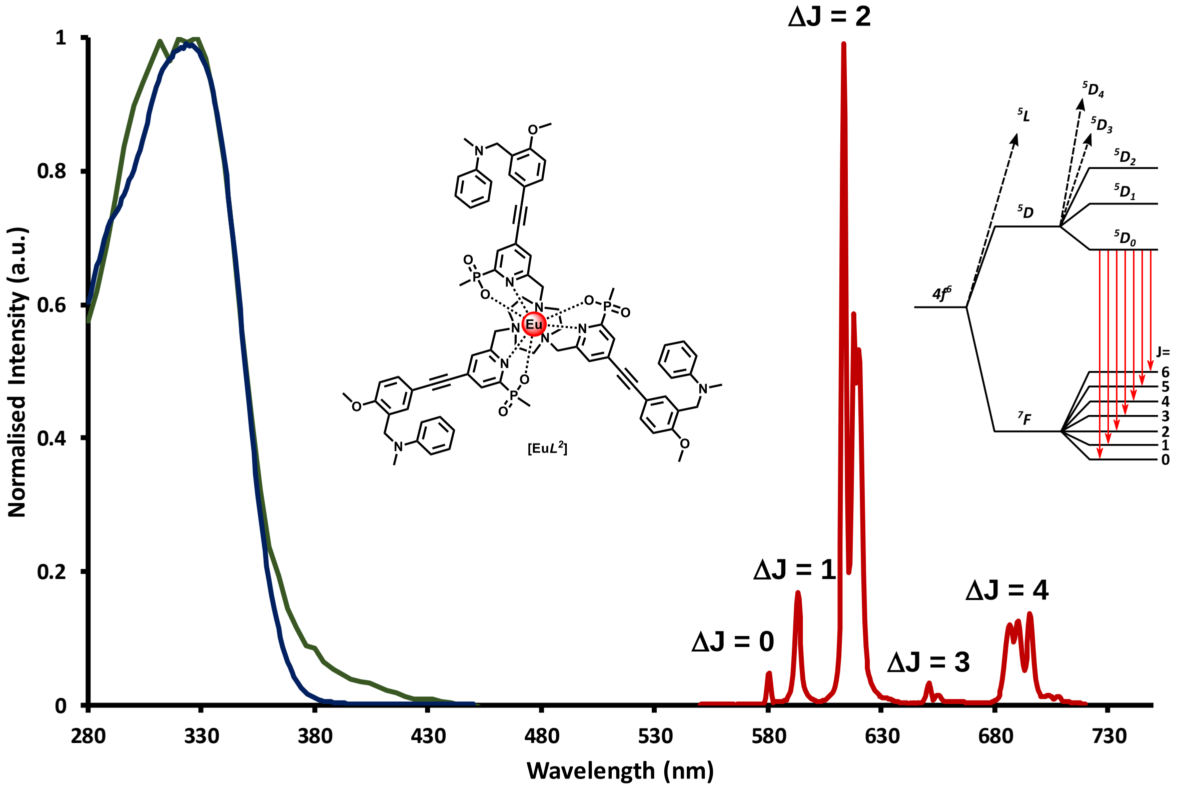

Figure S4. Absorption, excitation and metal-based emission spectra for [Eu*L*²] (c = 15 μM, λ_{exc} 331 nm, 295 K, 0.1 M NaCl), showing the observed transitions from the $^5D_0$ excited state.

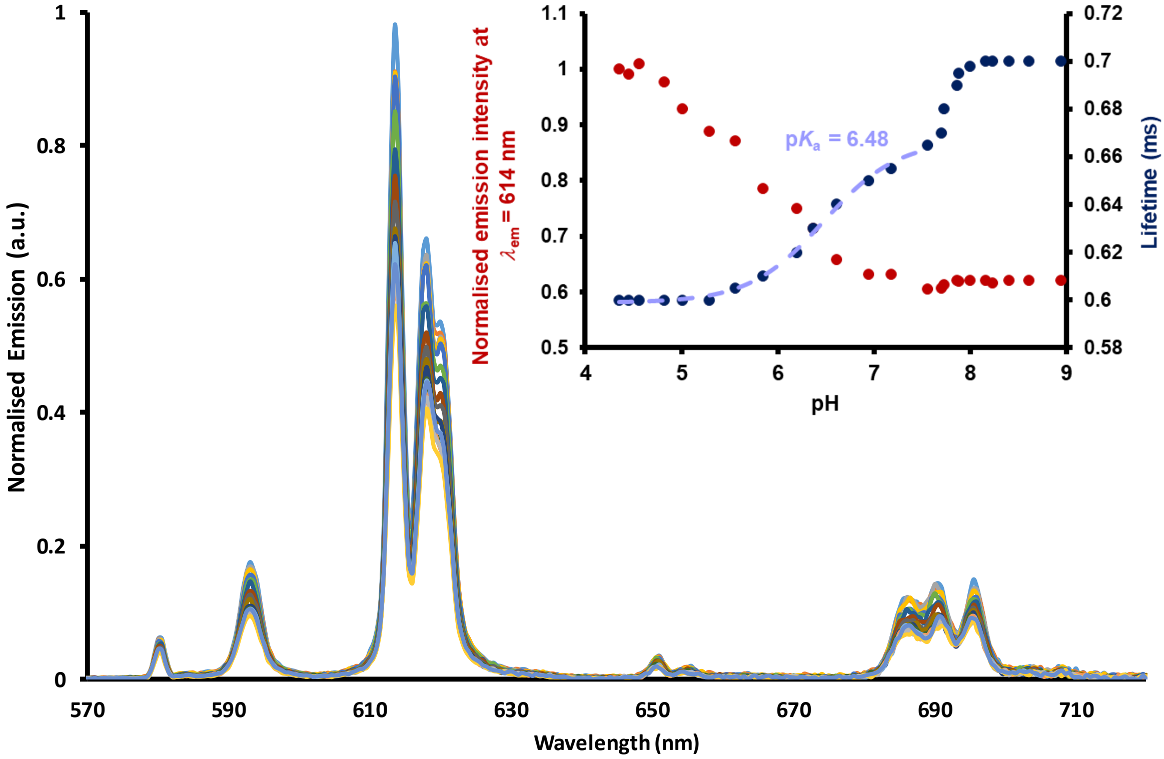

Figure S5. Variation of the europium emission spectrum and lifetime with pH for [EuL^2] (c = 15 µM, λ_{exc} 340 nm, 295 K, 0.1 M NaCl) showing the fit (line) to experimental data.

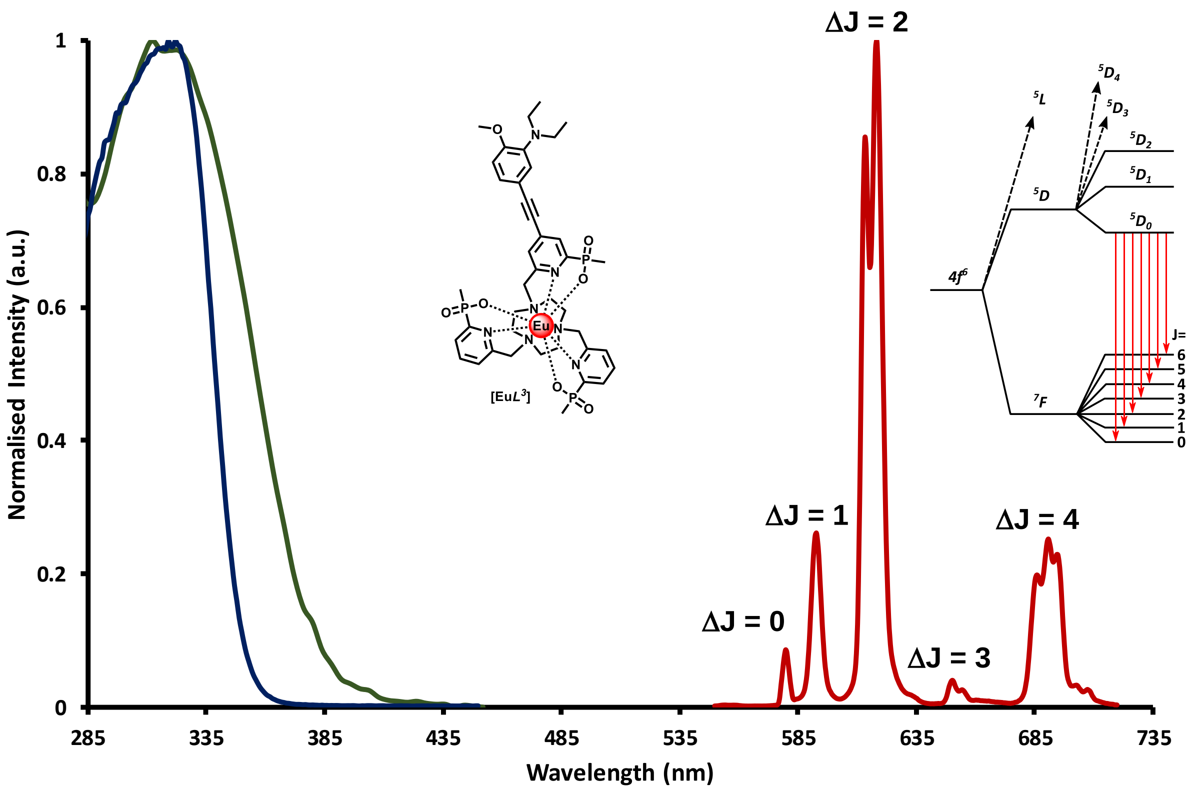

Figure S6. Absorption, excitation and metal-based emission spectra for [EuL^3], (c = 15 µM, λ_{exc} 331 nm, 295 K, 0.1 M NaCl), showing the observed transitions from the $^5D_0$ excited state.

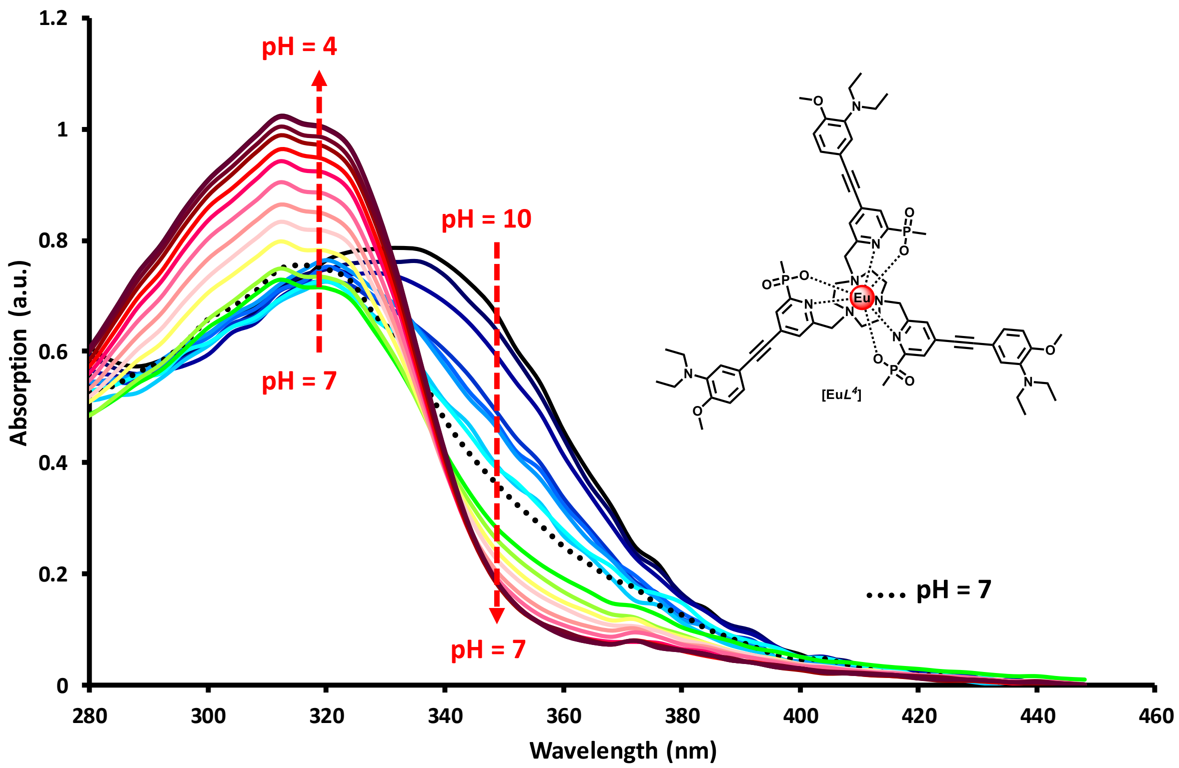

Figure S7. Variation of the absorption spectrum of [Eu***L***[4]] with pH revealing the absence of isosbestic points, as successive protonation shifts the energies of the singlet excited states of each independent chromophore (c = 15 µM, 295 K, 0.1 M NaCl).

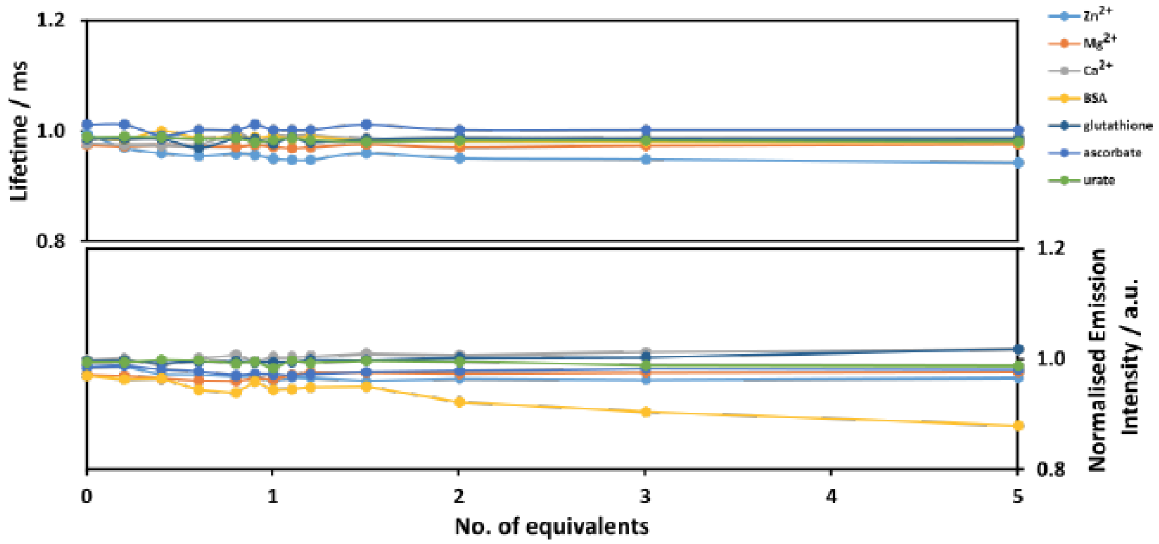

Figure S8: Variation in emission lifetime (*top*, λ_{exc} 332 nm, λ_{em} 613 nm) and normalised emission intensity (*bottom*, λ_{exc} 332 nm) for [Eu***L***[4]] with the number of equivalents of the stated analytes ($c_{complex}$ = 30 µM, 0.1 M NaCl, 0.1 M NH_4HCO_3 buffer, pH 5).

Cell Imaging

The europium complex [Eu*L*[4]] (3 & 30 μM) was incubated with NIH-3T3 or MCF-7 cells for up to 24 h to allow complex uptake within the lysosomes; this localisation profile was verified by co-incubation with LysoTracker Green, using the same methods reported in the literature, (see main text).

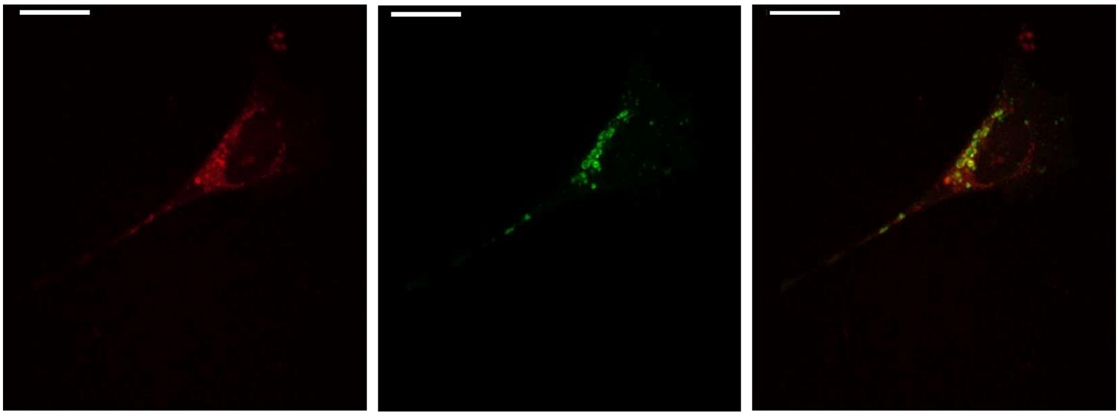

Figure S9. Nigericin experiment (Scale bar = 20 μm; 16 h post incubation in NIH-3T3 cells): (Left) [Eu*L*[4]]; (Centre) Lysotracker Green; (Right) Overlay showing correspondence.

The complex was found to be brightest in the NIH-3T3 cells at around 16 h. At 16 h, nigericin was added (K^+/H^+ ionophore) in order to induce a pH increase in the lysosomes. A minor amount of the complex goes to mitochondria and the lysosomes swelled, increasing in size. The measured brightness dropped by approximately 30% with this induced pH change, accounted for the fact that some complex has migrated to the mitochondria, whilst the size of the lysosomes also has increased, i.e. there was a lower amount of the complex in a larger volume.

Cell toxicity was assessed using a standard MTT assay,[4] and showed no change in the percentage of viable cells over the concentration range shown (Figure S10).

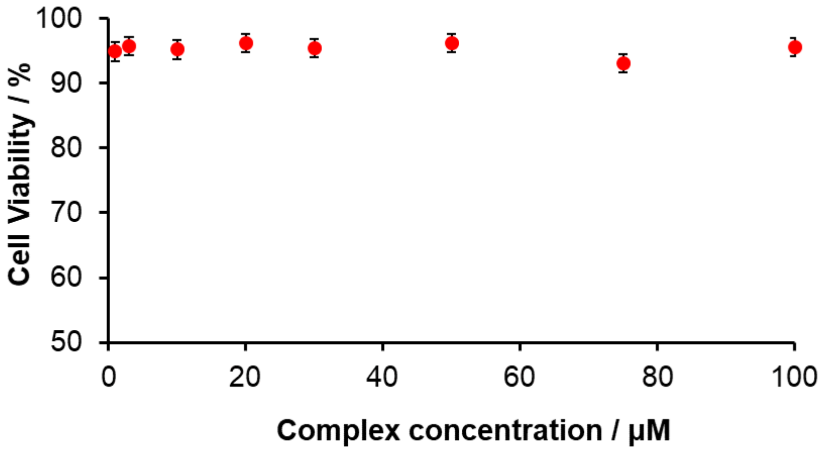

Figure S10. MTT assay showing the percentage of living viable cells as a function of [Eu*L*[4]] concentration for a 24 h incubation.

Aliquots were taken during the 30 µM cell study at 2, 4, 8, 12, 16 and 24 hours and subjected to ICP-MS analysis to quantify the amount of [Eu$\boldsymbol{L^4}$] present.

These samples were prepared for analysis by sample digestion in nitric acid for 24 hours at room temperature. Eu-ICP Mass Spectrometry analyses were performed by Durham University, Department of Earth Sciences. These ICPMS results (below) supported the observed probe brightness profile with the steady uptake of [Eu$\boldsymbol{L^4}$] until maximum brightness is observed at 16 hours.

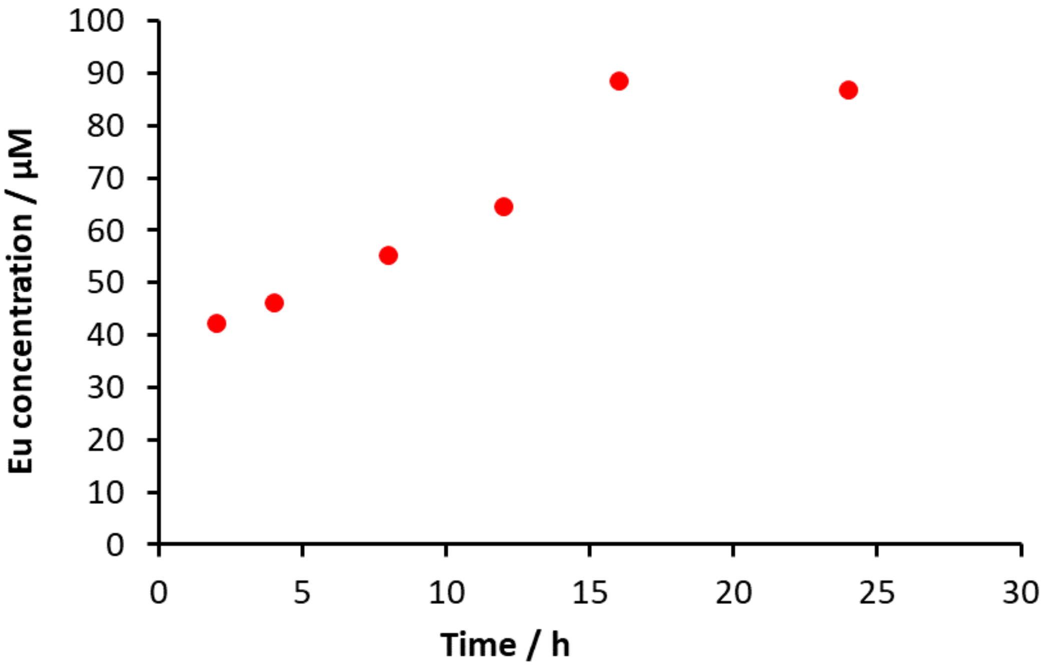

Figure S8. Eu concentrations determined by ICP-MS at the stated time points, for [Eu$\boldsymbol{L^4}$] in NIH-3T3 cells showing the rough doubling of complex uptake over the time period of 2 to 16 h.

NMR Spectra of Intermediates and Ligands

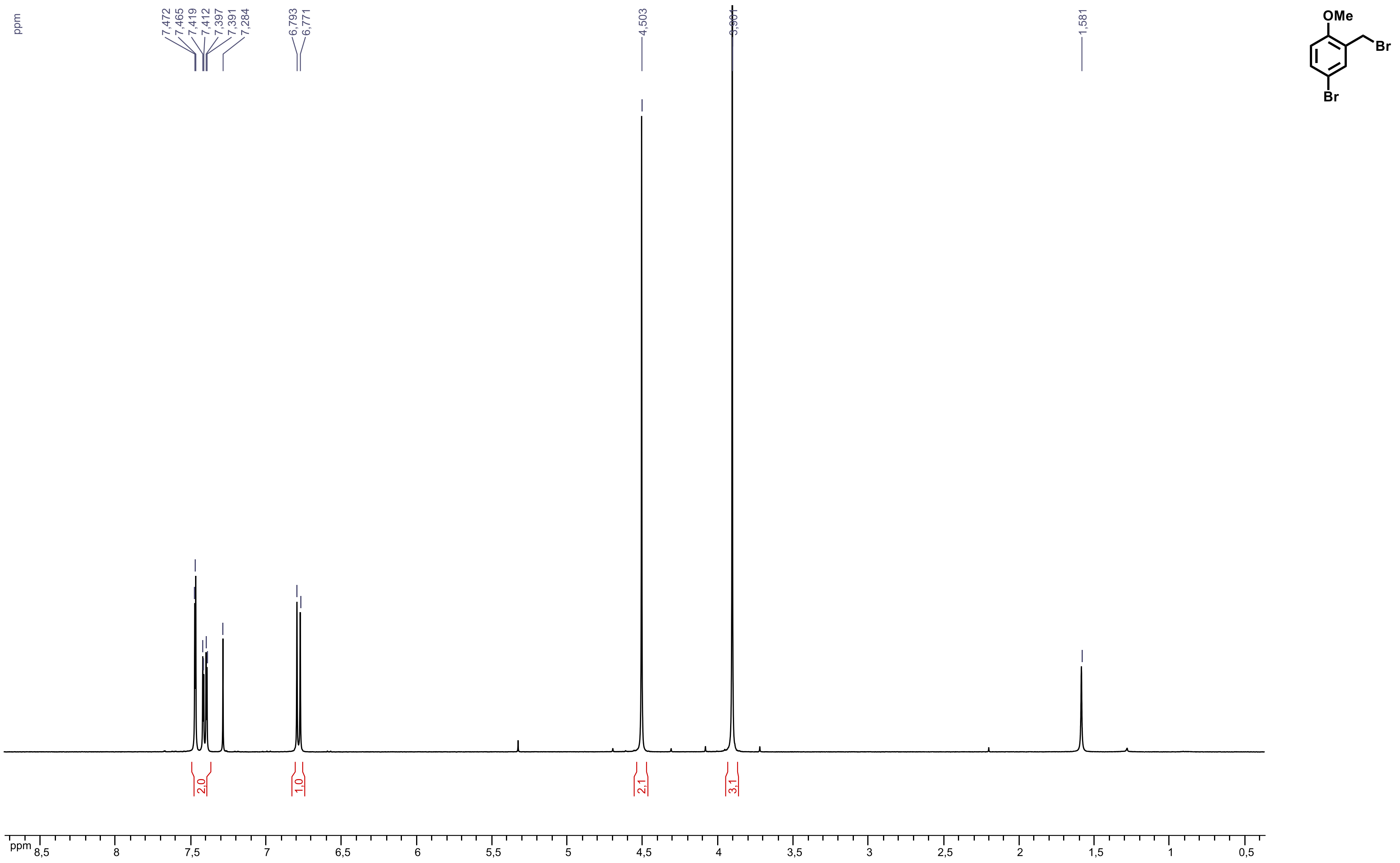

Figure S9. ^{1}H NMR ($CDCl_3$, 400 MHz) spectrum of compound **1**

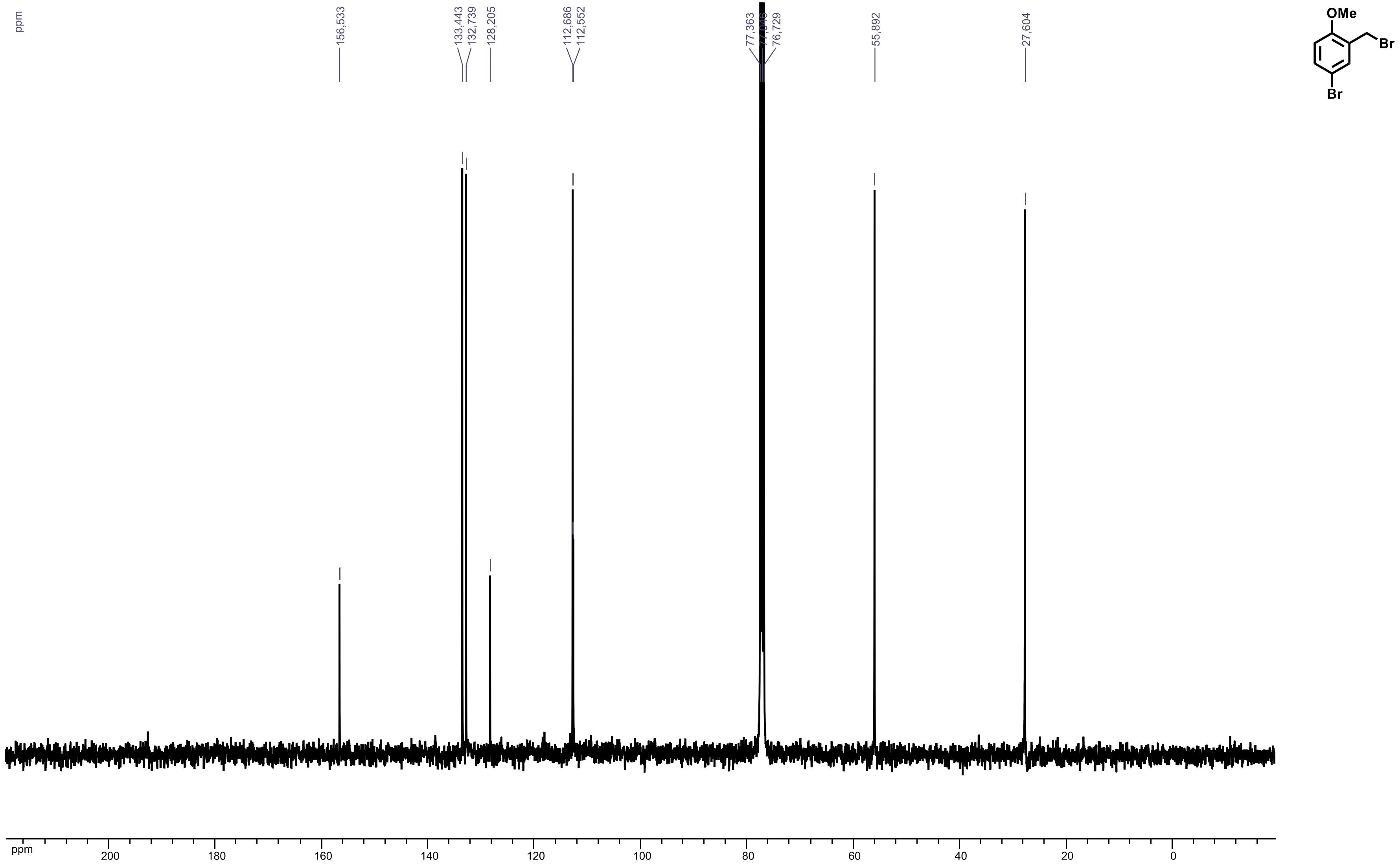

Figure S10. ^{13}C NMR ($CDCl_3$, 100 MHz) spectrum of compound **1**

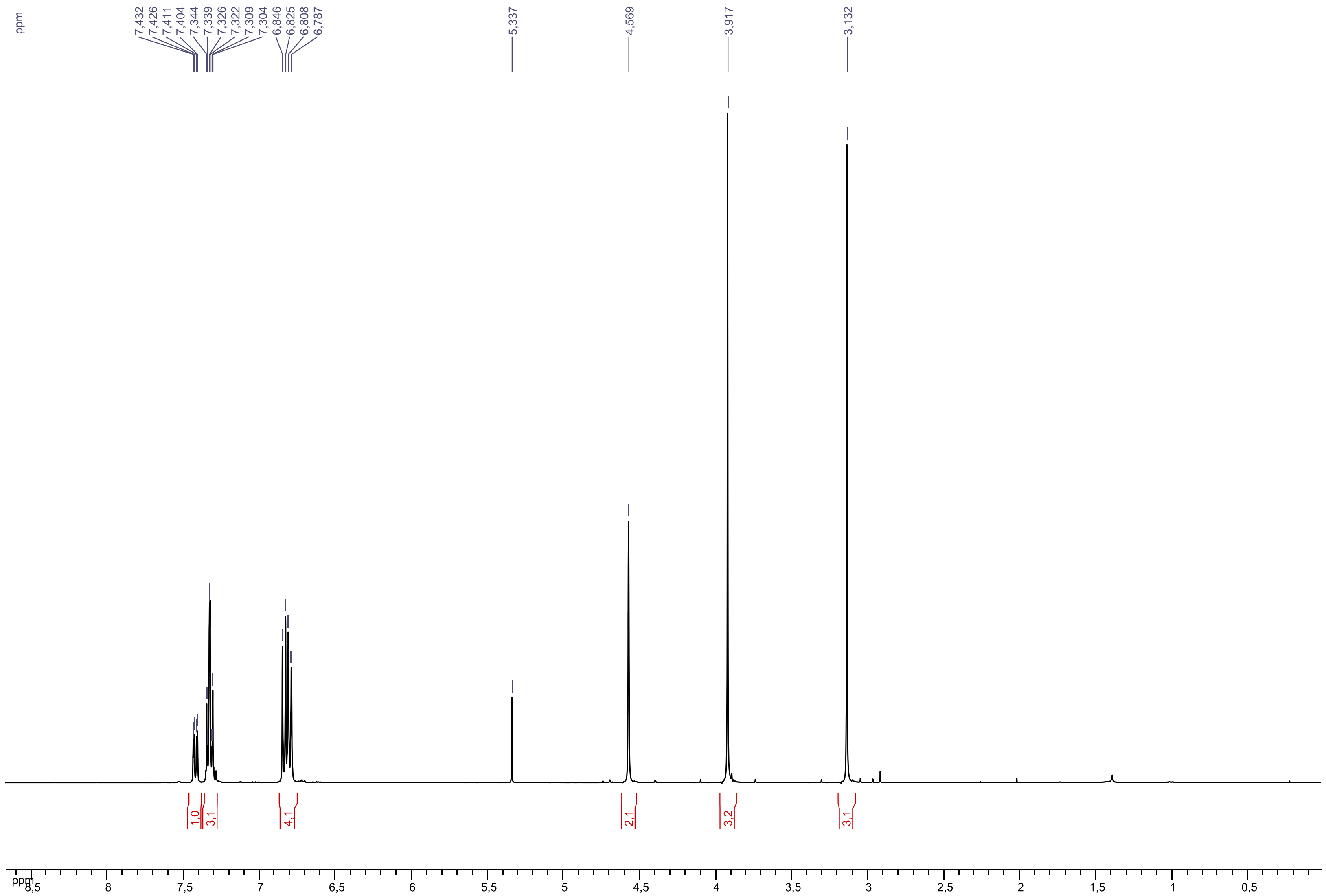

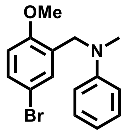

Figure S11. ^{1}H NMR ($CDCl_3$, 400 MHz) spectrum of compound **2**

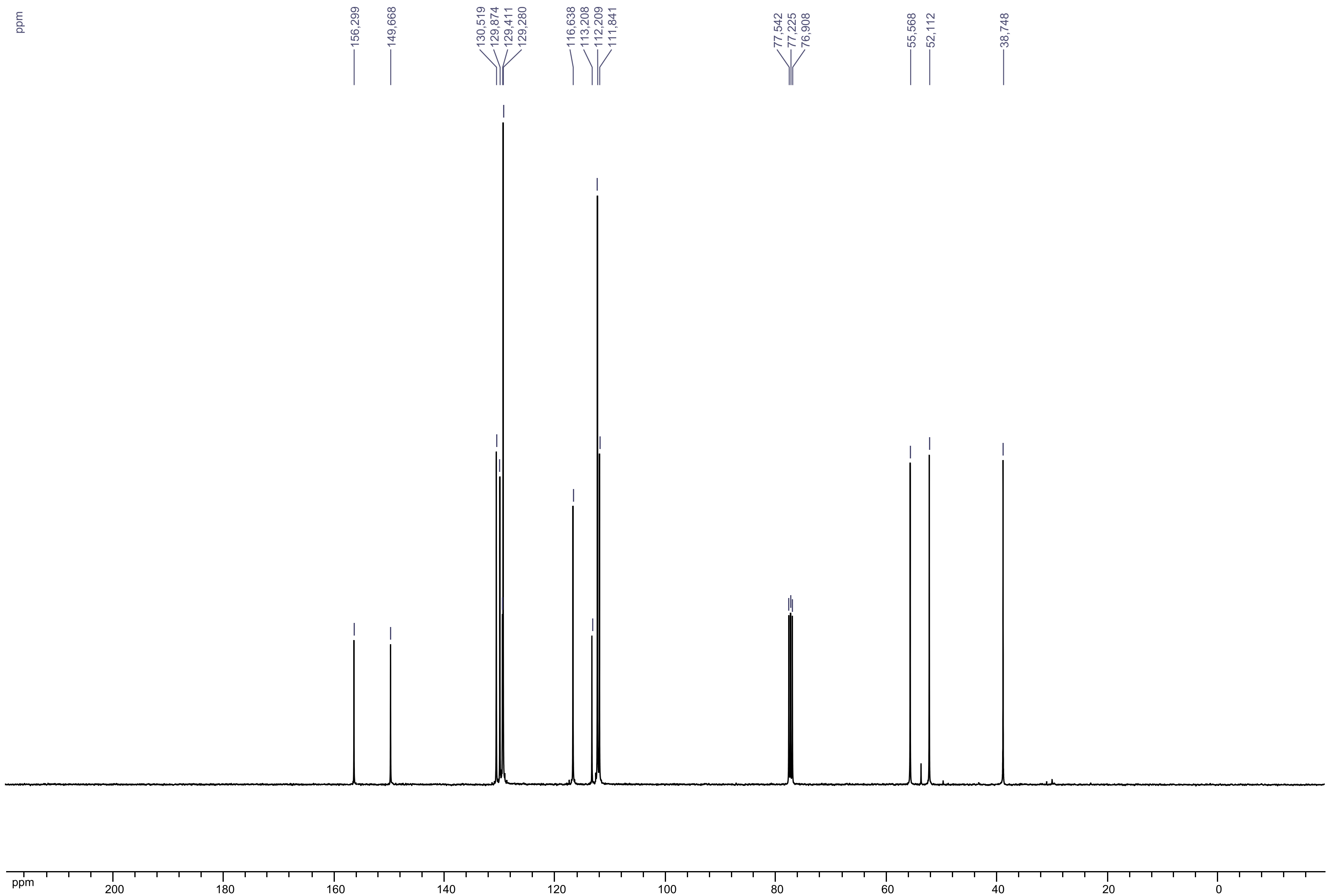

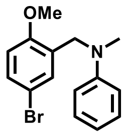

Figure S12. ^{13}C NMR ($CDCl_3$, 100 MHz) spectrum of compound **2**

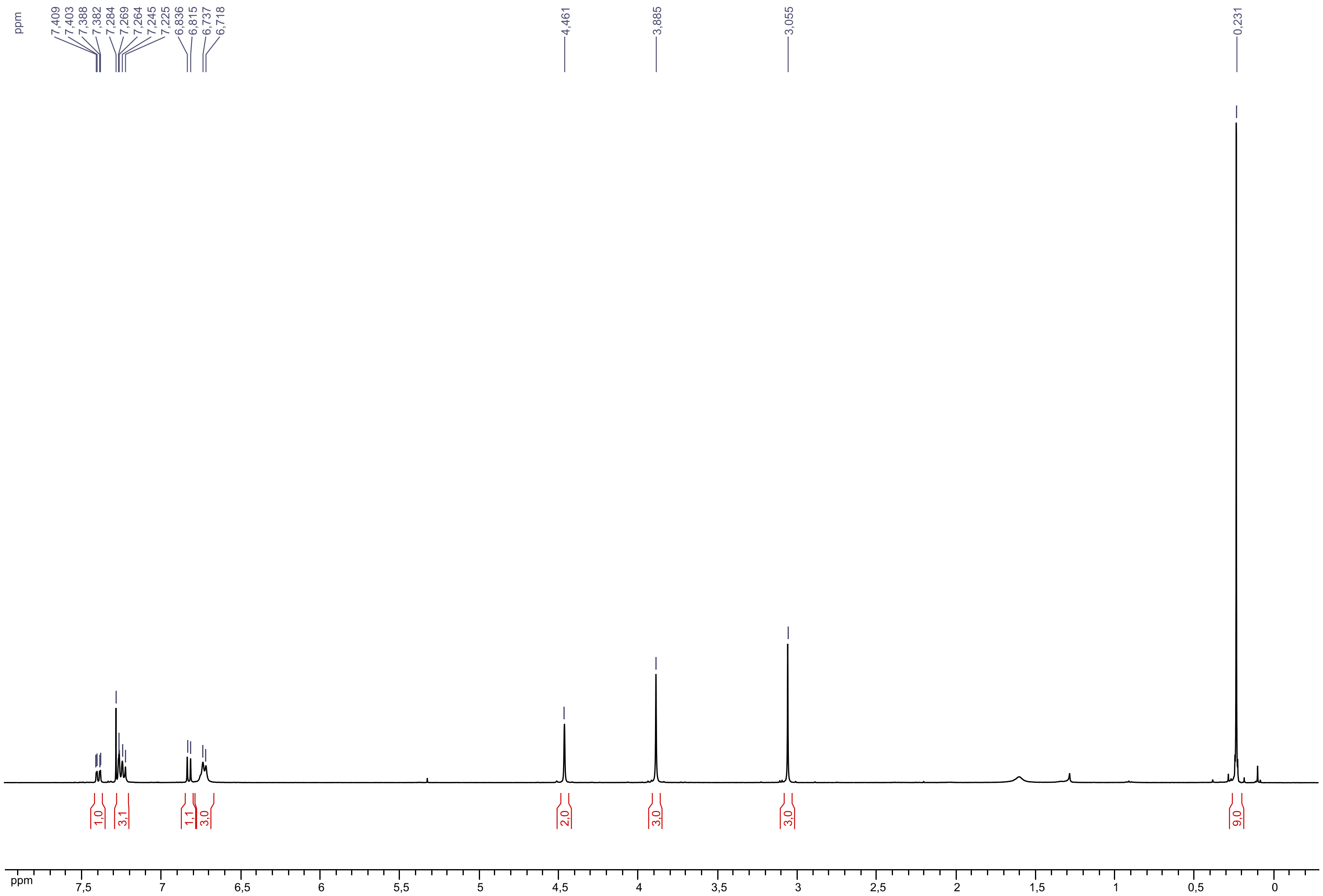

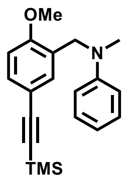

Figure S13. ^{1}H NMR ($CDCl_3$, 400 MHz) spectrum of compound **3**

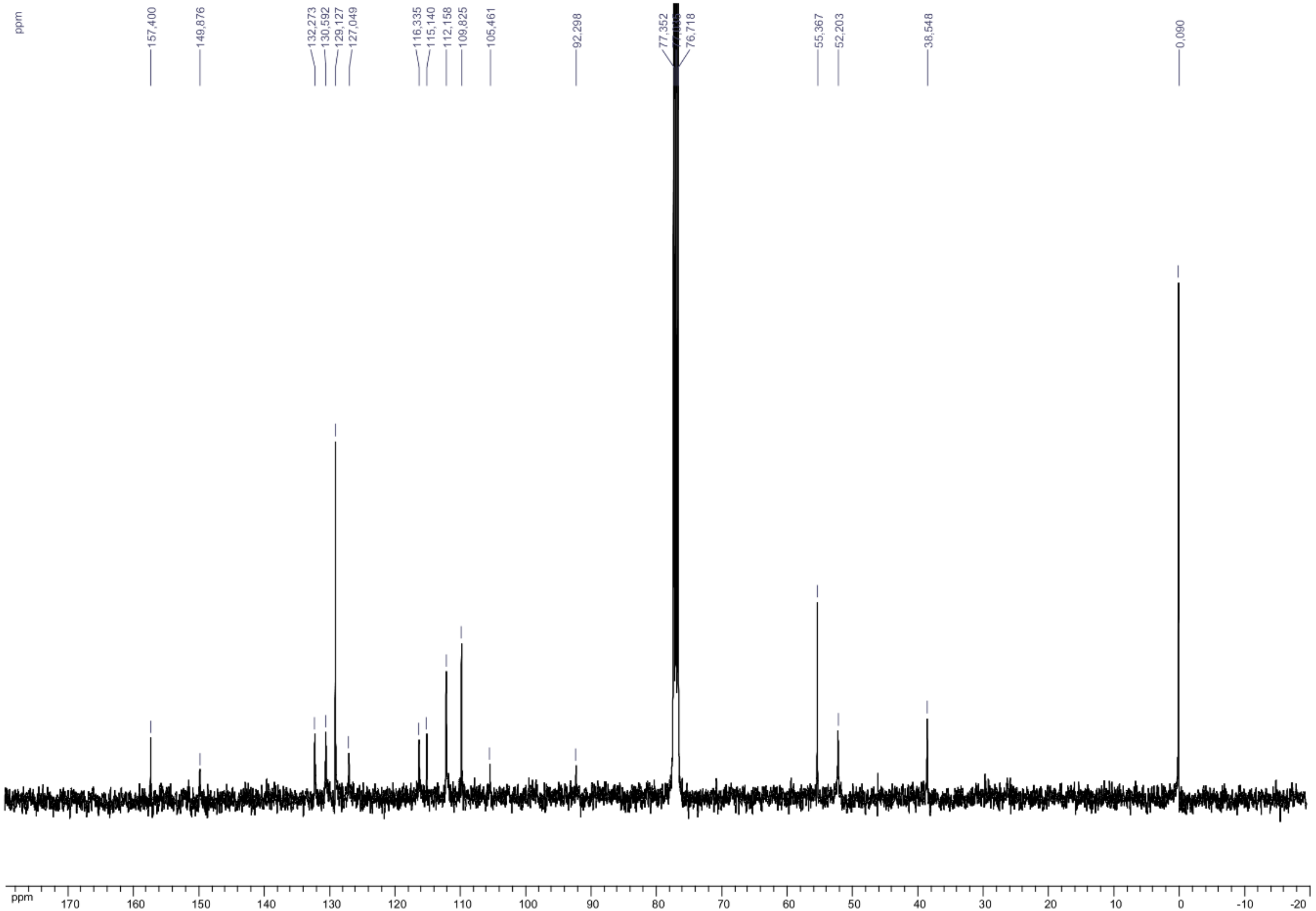

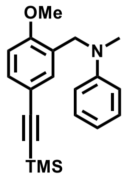

Figure S14. ^{13}C NMR ($CDCl_3$, 100 MHz) spectrum of compound **3**

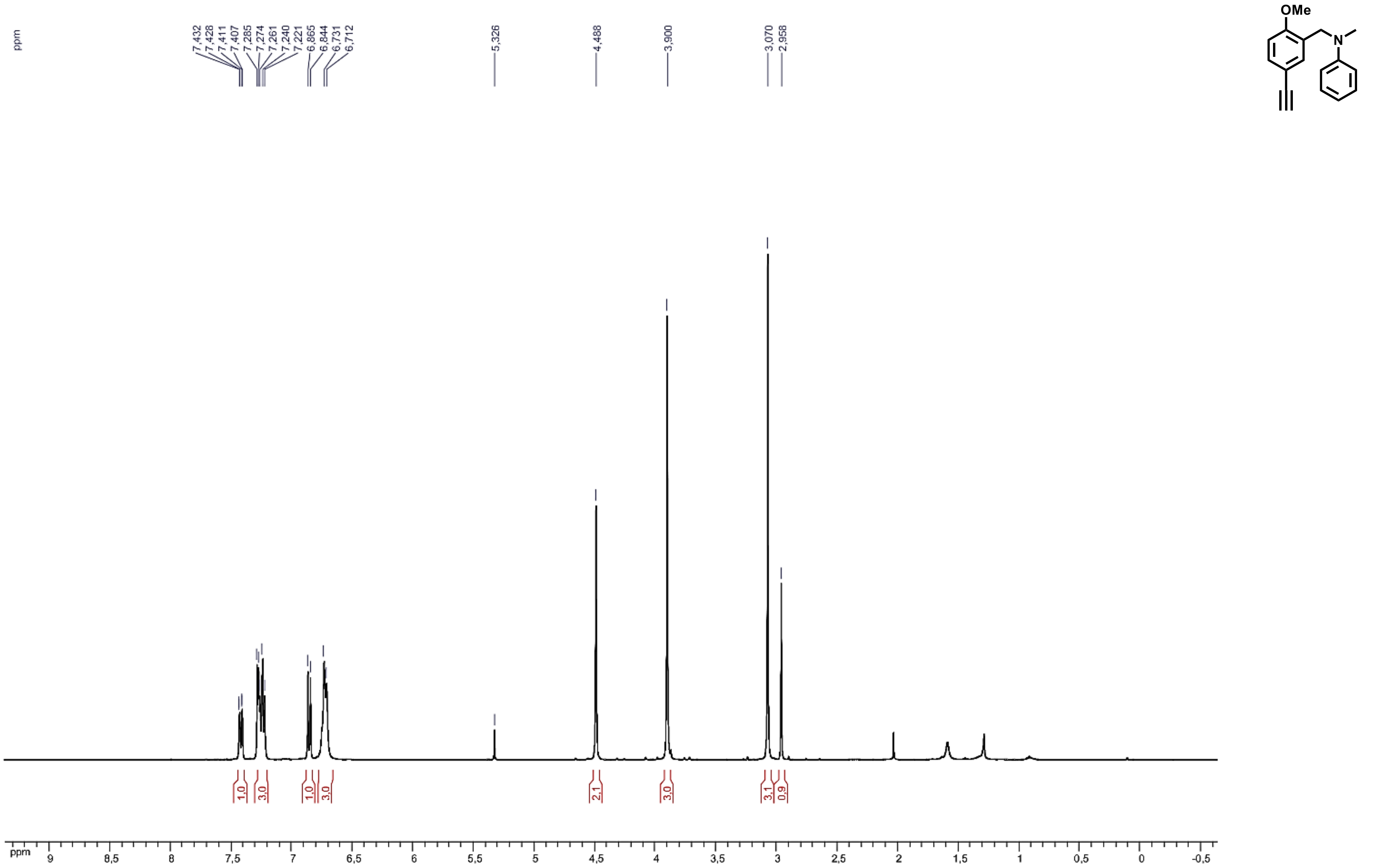

Figure S15. ^1H NMR ($CDCl_3$, 400 MHz) spectrum of compound **4**

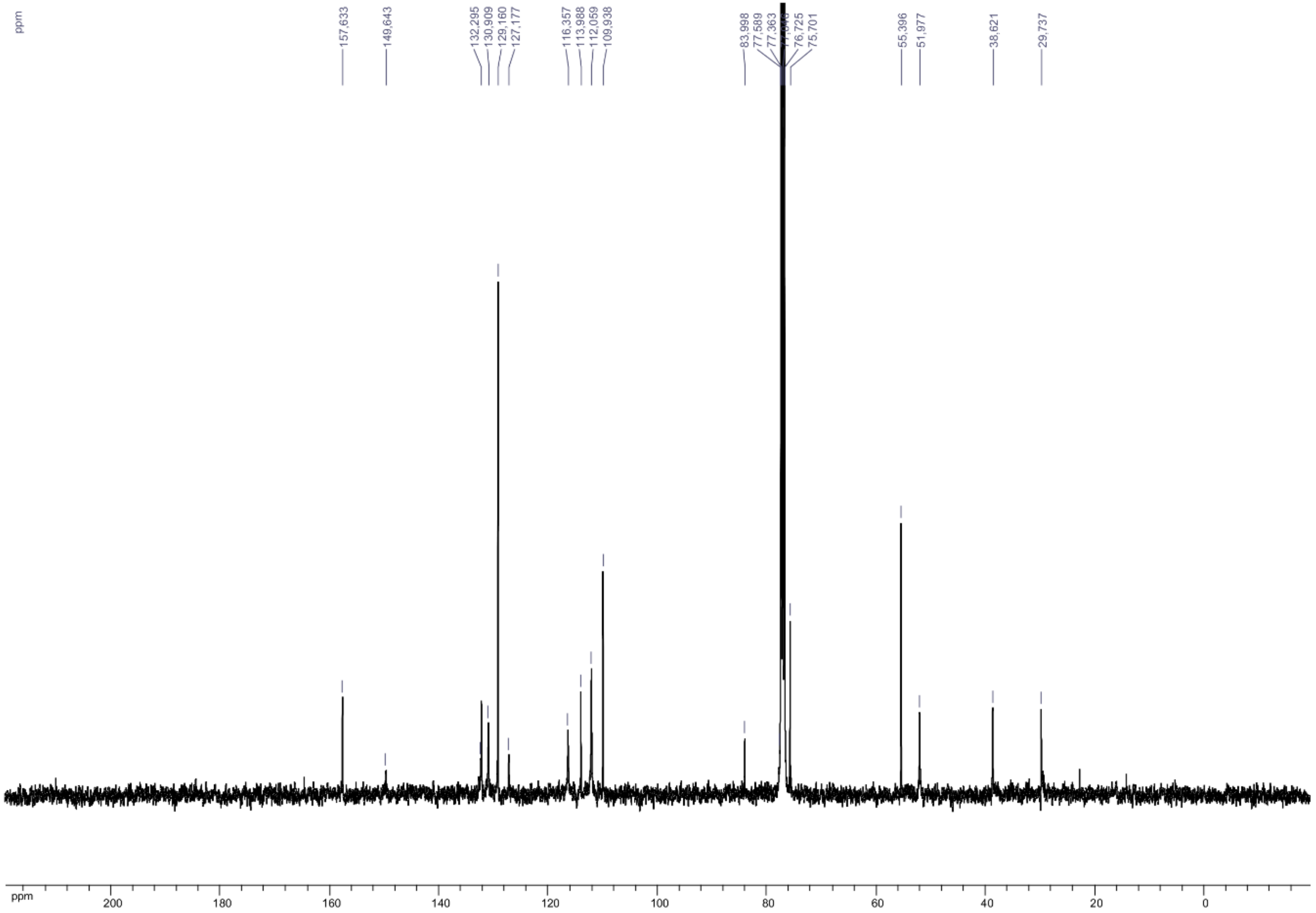

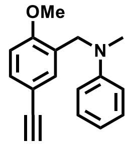

Figure S16. ^{13}C NMR ($CDCl_3$, 100 MHz) spectrum of compound **4**

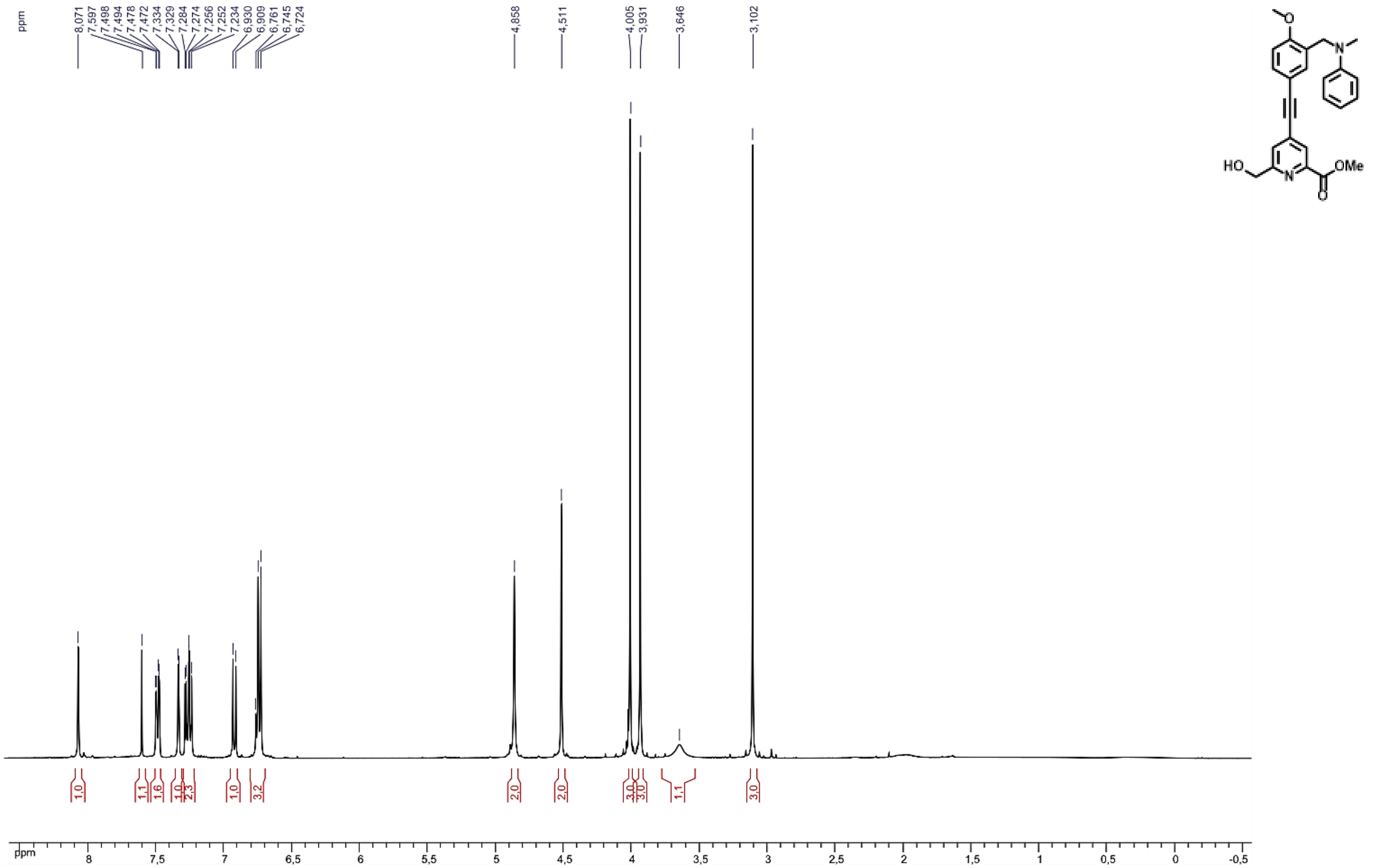

Figure S17. ^{1}H NMR ($CDCl_3$, 400 MHz) spectrum of compound **5**

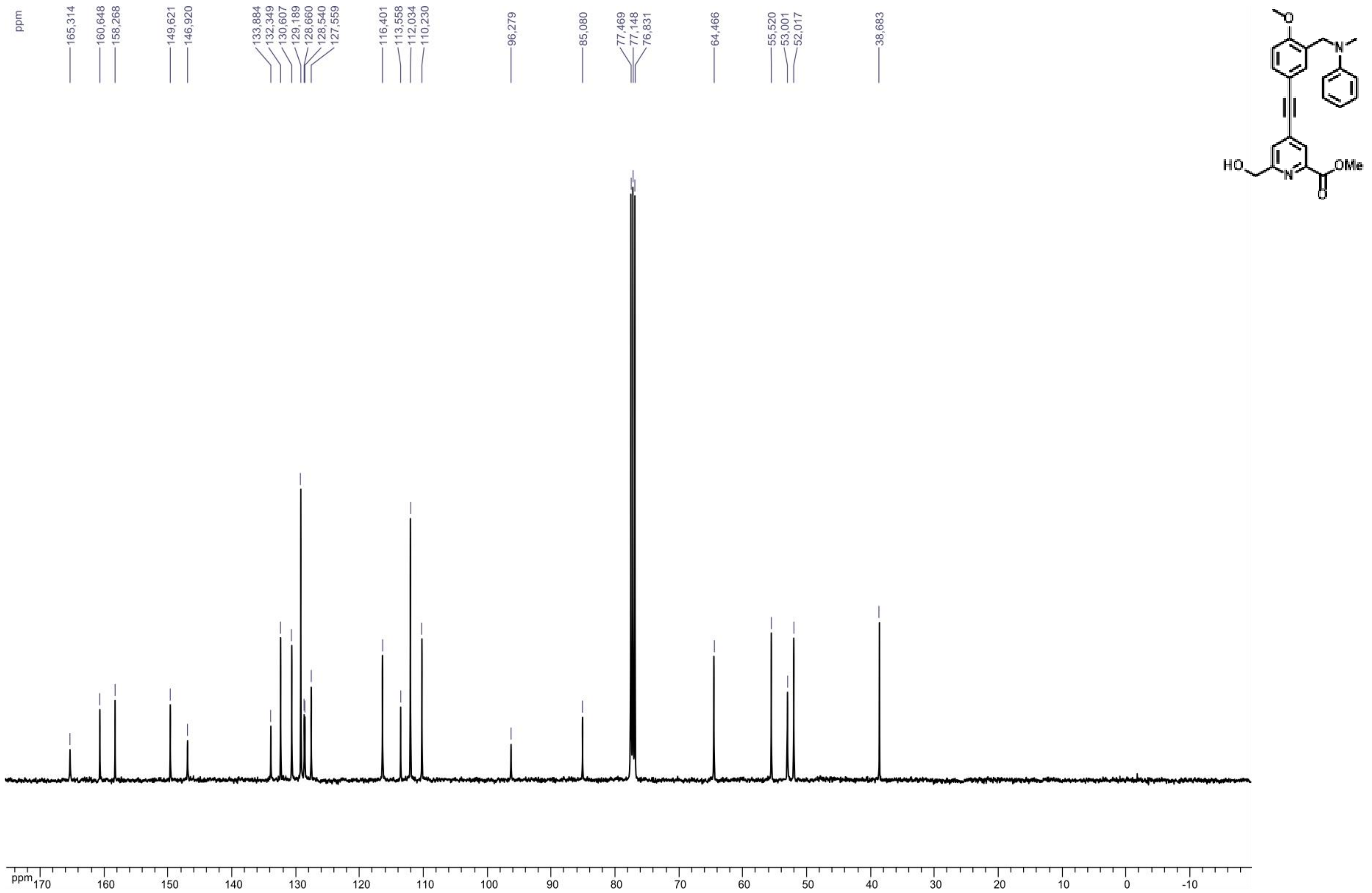

Figure S18. ^{13}C NMR ($CDCl_3$, 100 MHz) spectrum of compound **5**

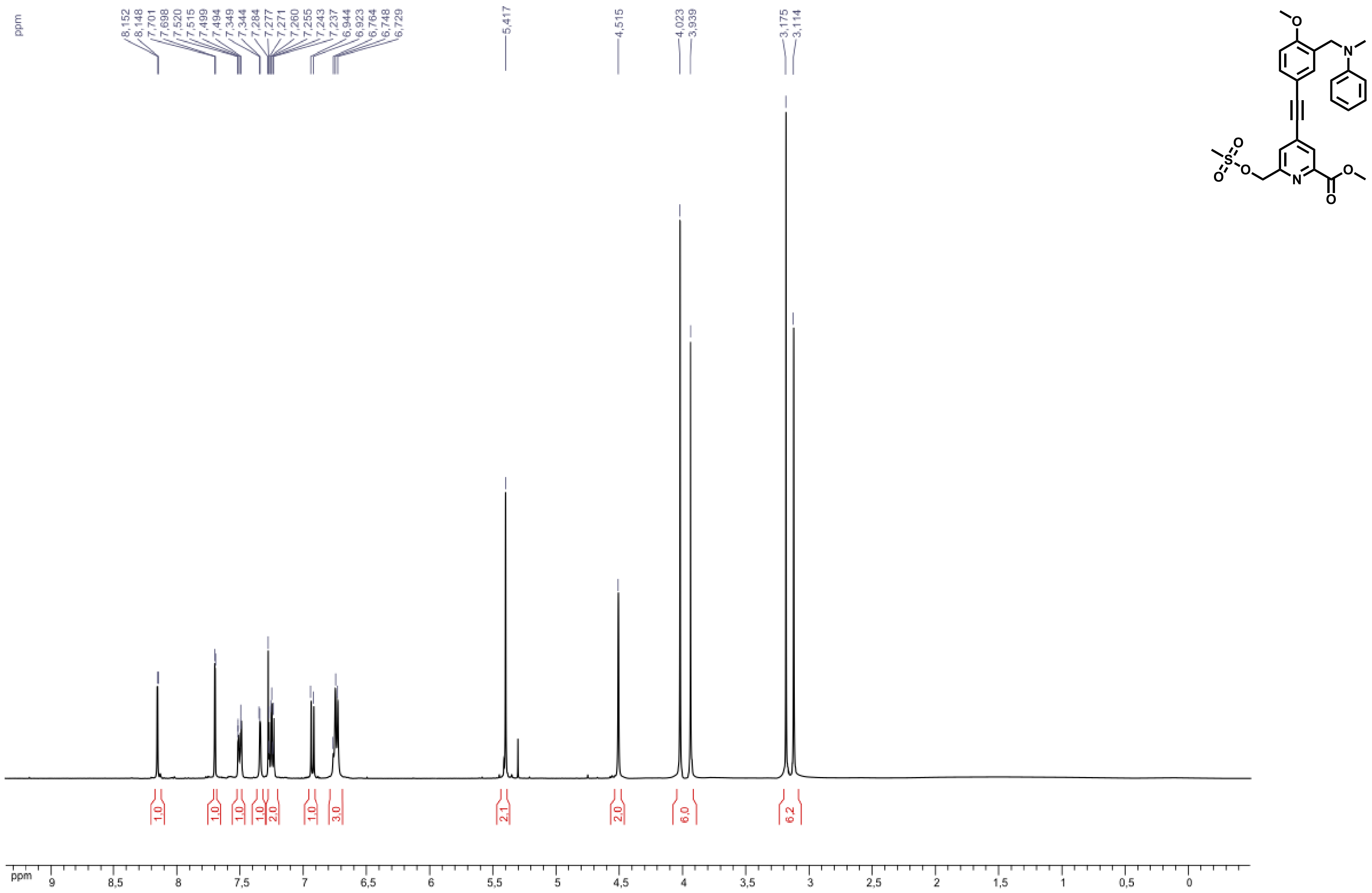

Figure S19. ^{1}H NMR ($CDCl_3$, 400 MHz) spectrum of compound **6**

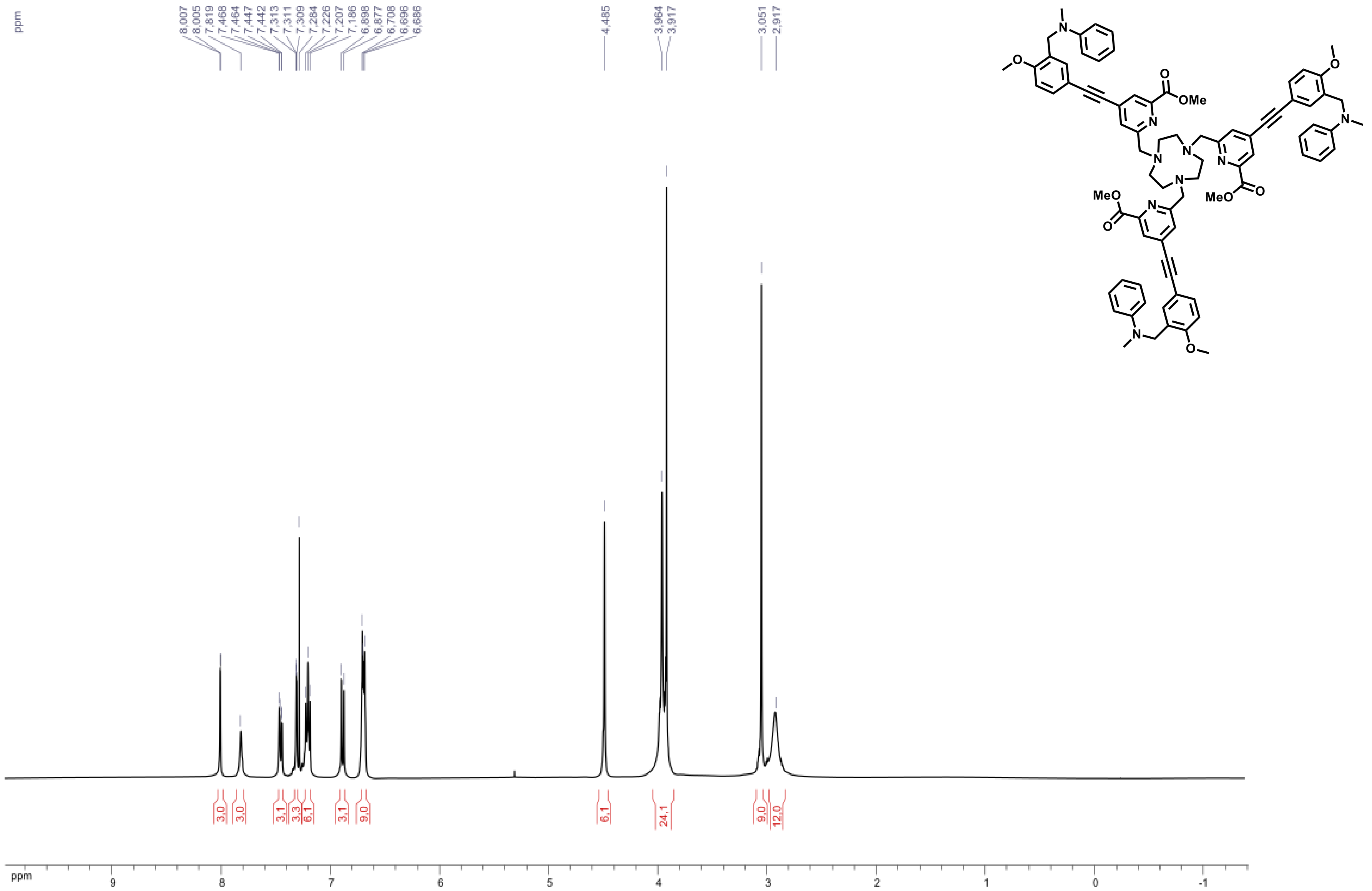

Figure S20. ^{1}H NMR ($CDCl_3$, 400 MHz) spectrum of compound **7**

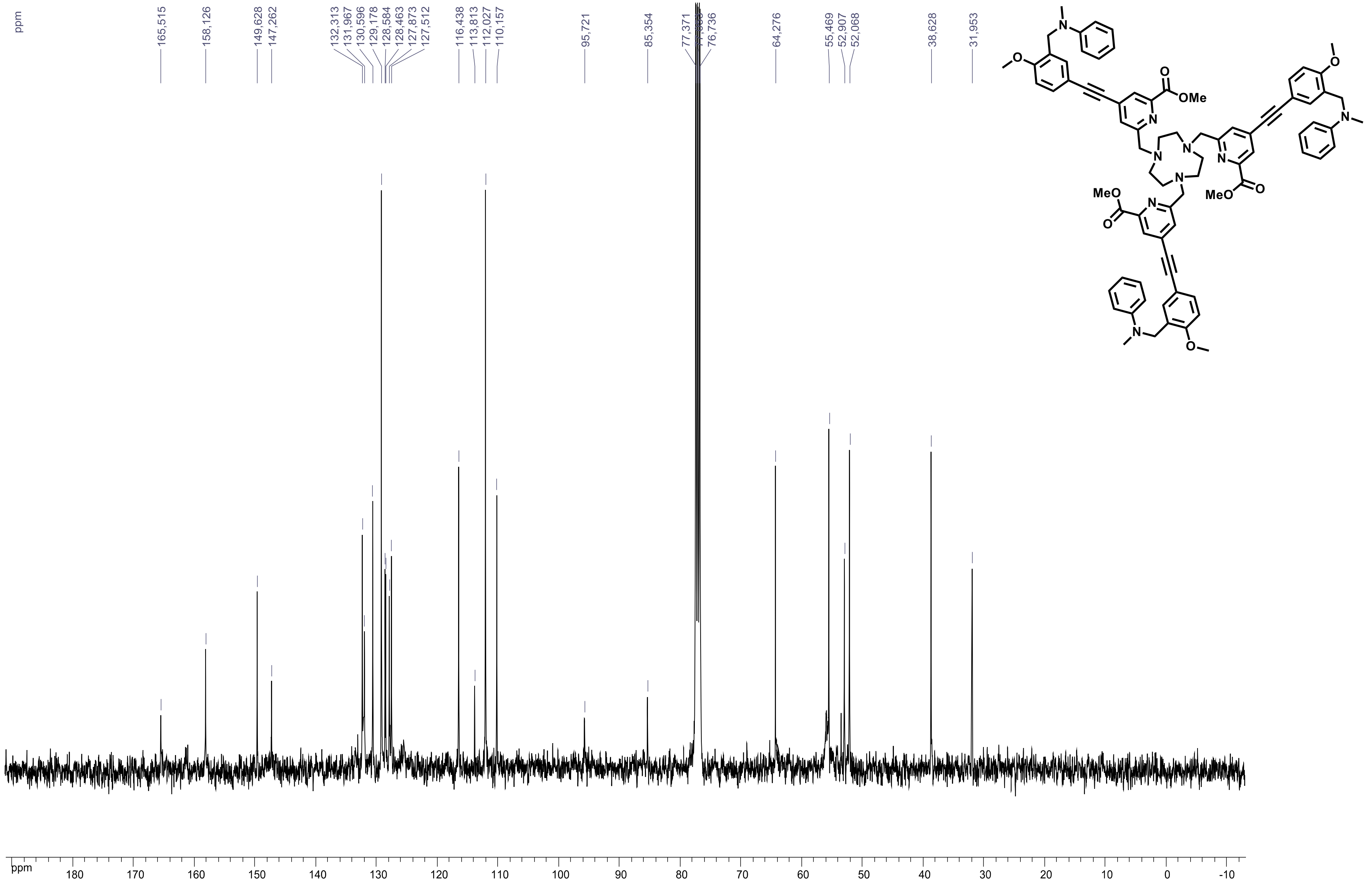

Figure S21. ^{13}C NMR ($CDCl_3$, 100 MHz) spectrum of compound **7**

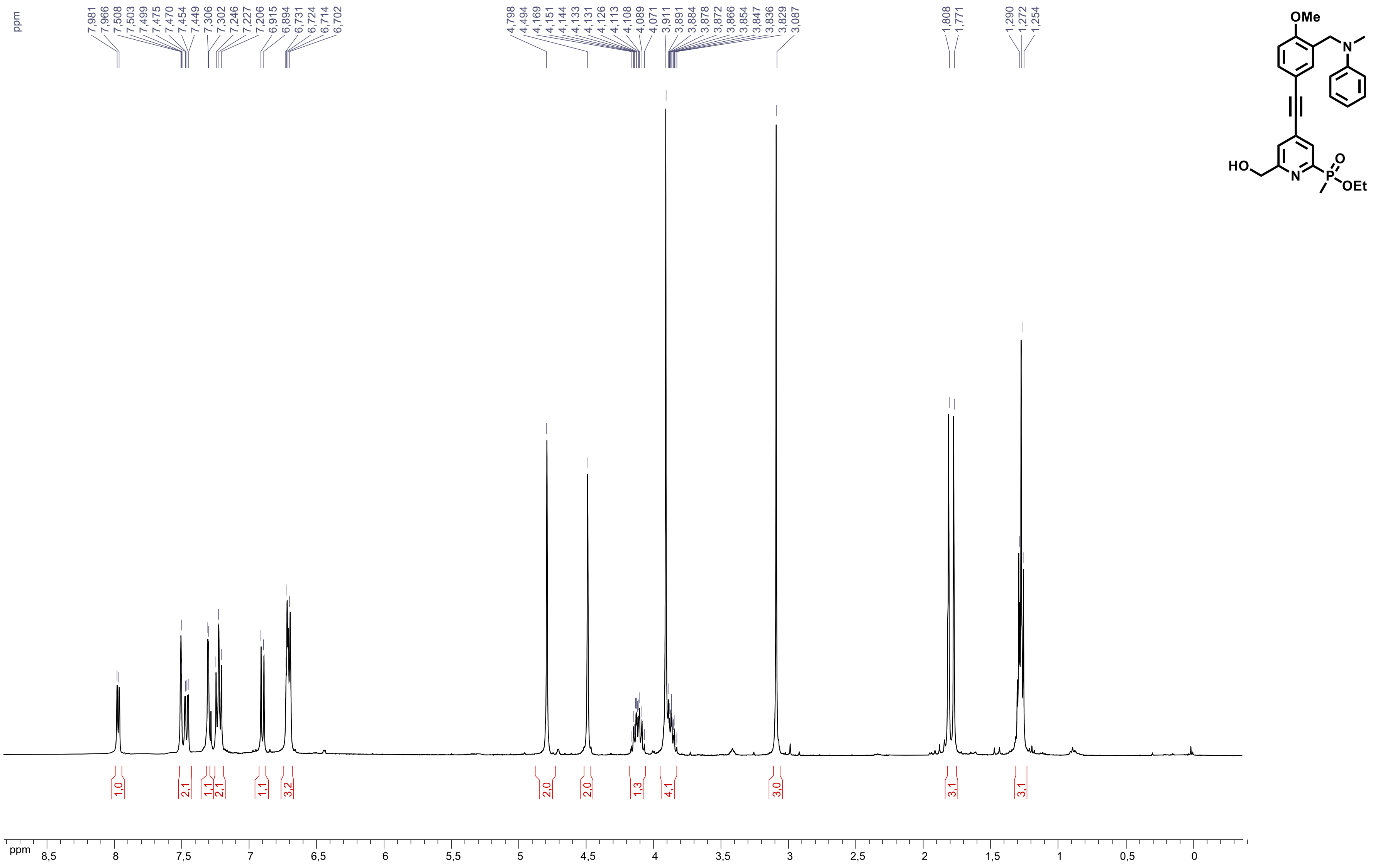

Figure S22. ^{1}H NMR ($CDCl_3$, 400 MHz) spectrum of compound **10**

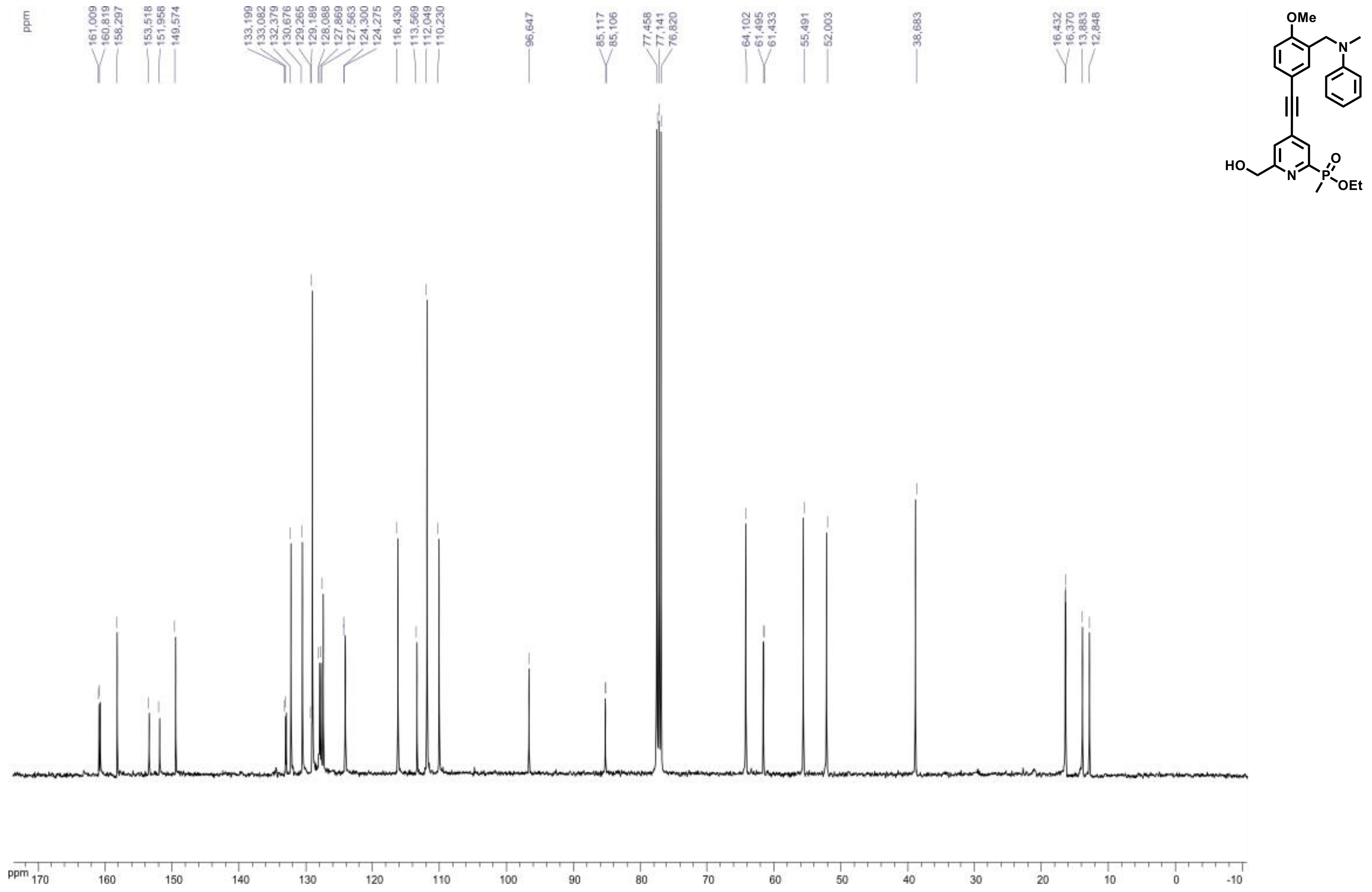

Figure S23. ^{13}C NMR ($CDCl_3$, 100 MHz) spectrum of compound **10**

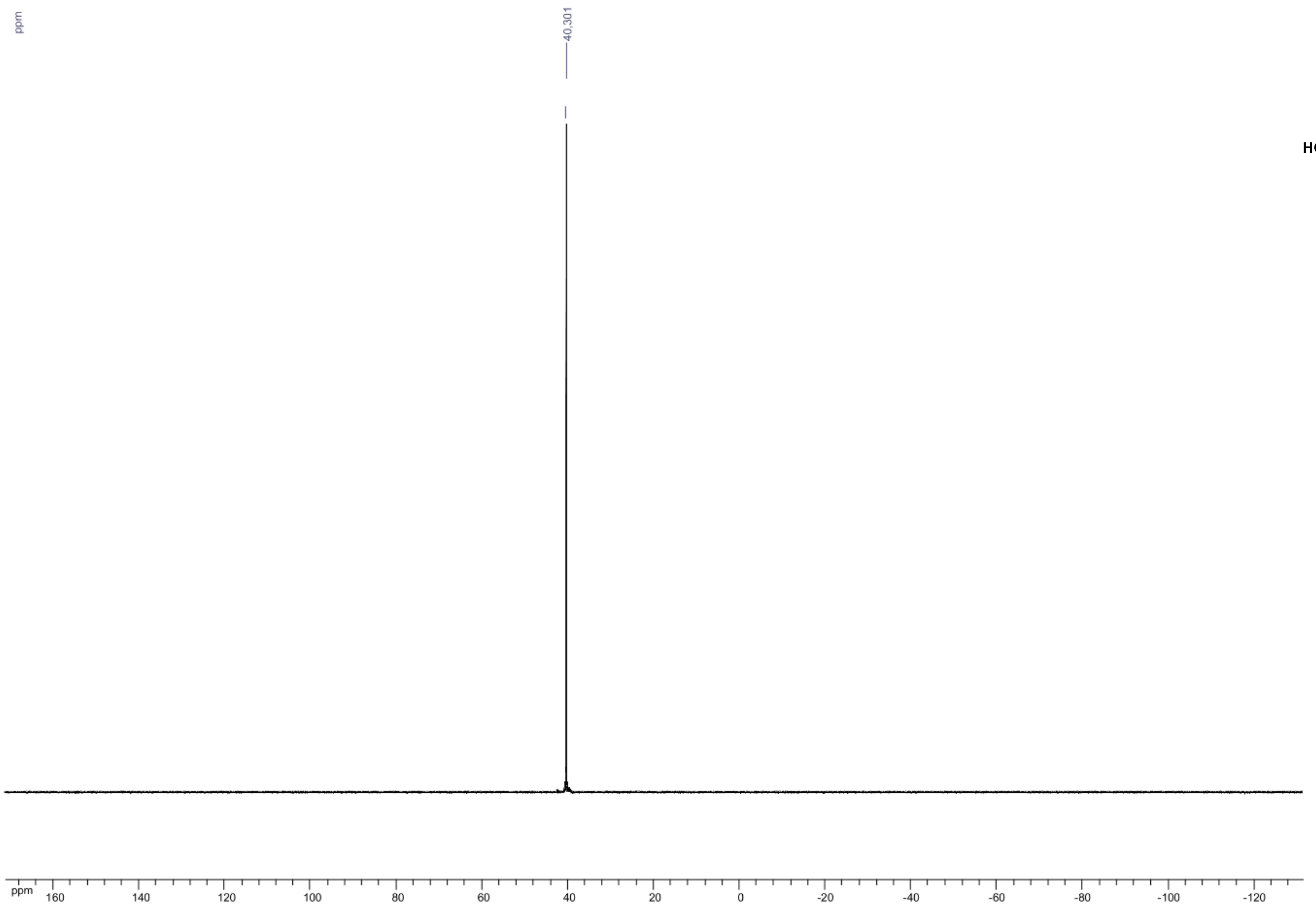

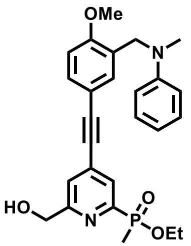

Figure S24. ^{31}P NMR ($CDCl_3$, 162 MHz) spectrum of compound **10**

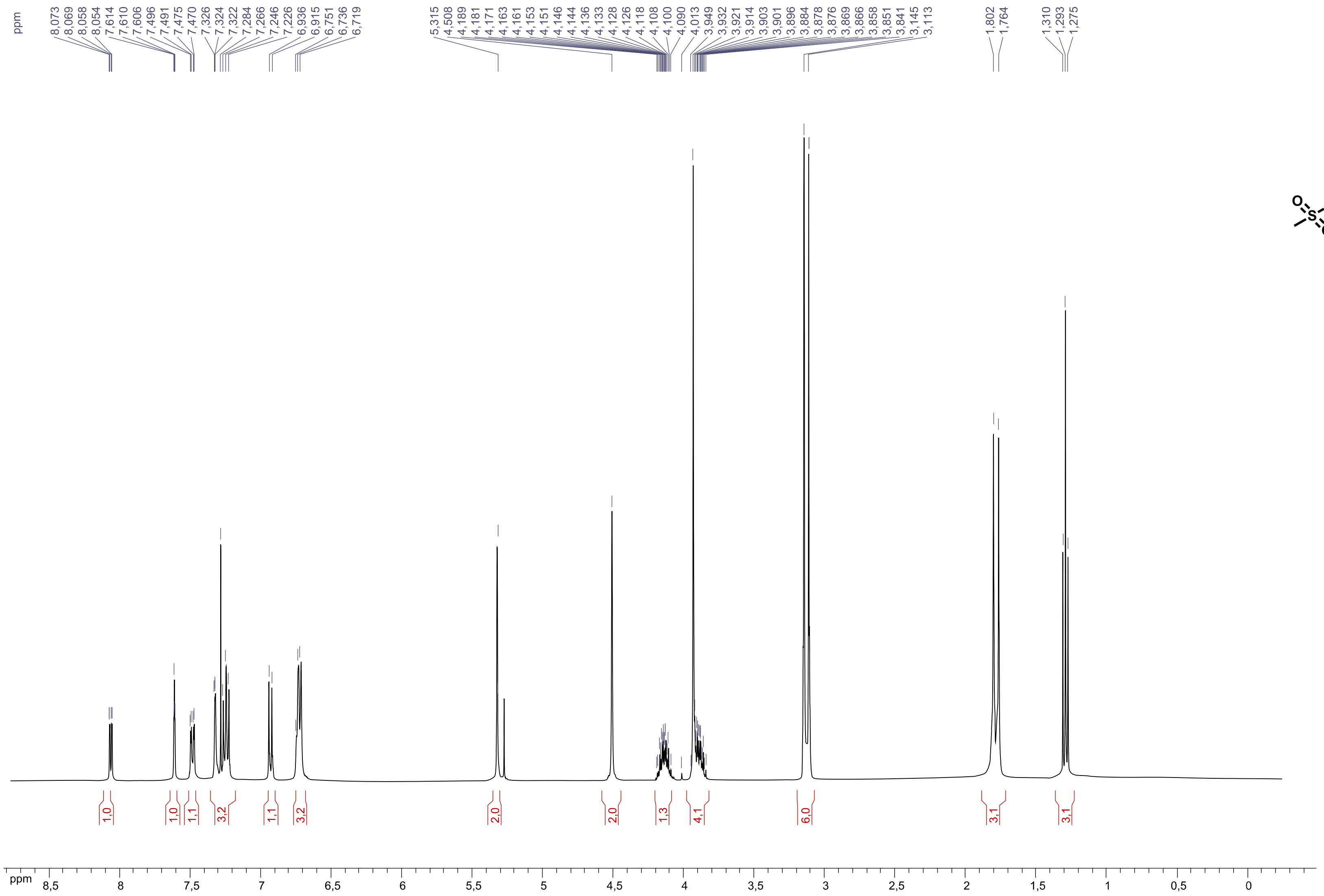

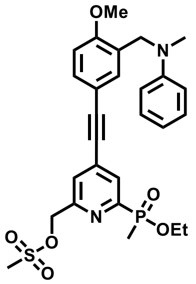

Figure S25. ^{1}H NMR ($CDCl_3$, 400 MHz) spectrum of compound **11**

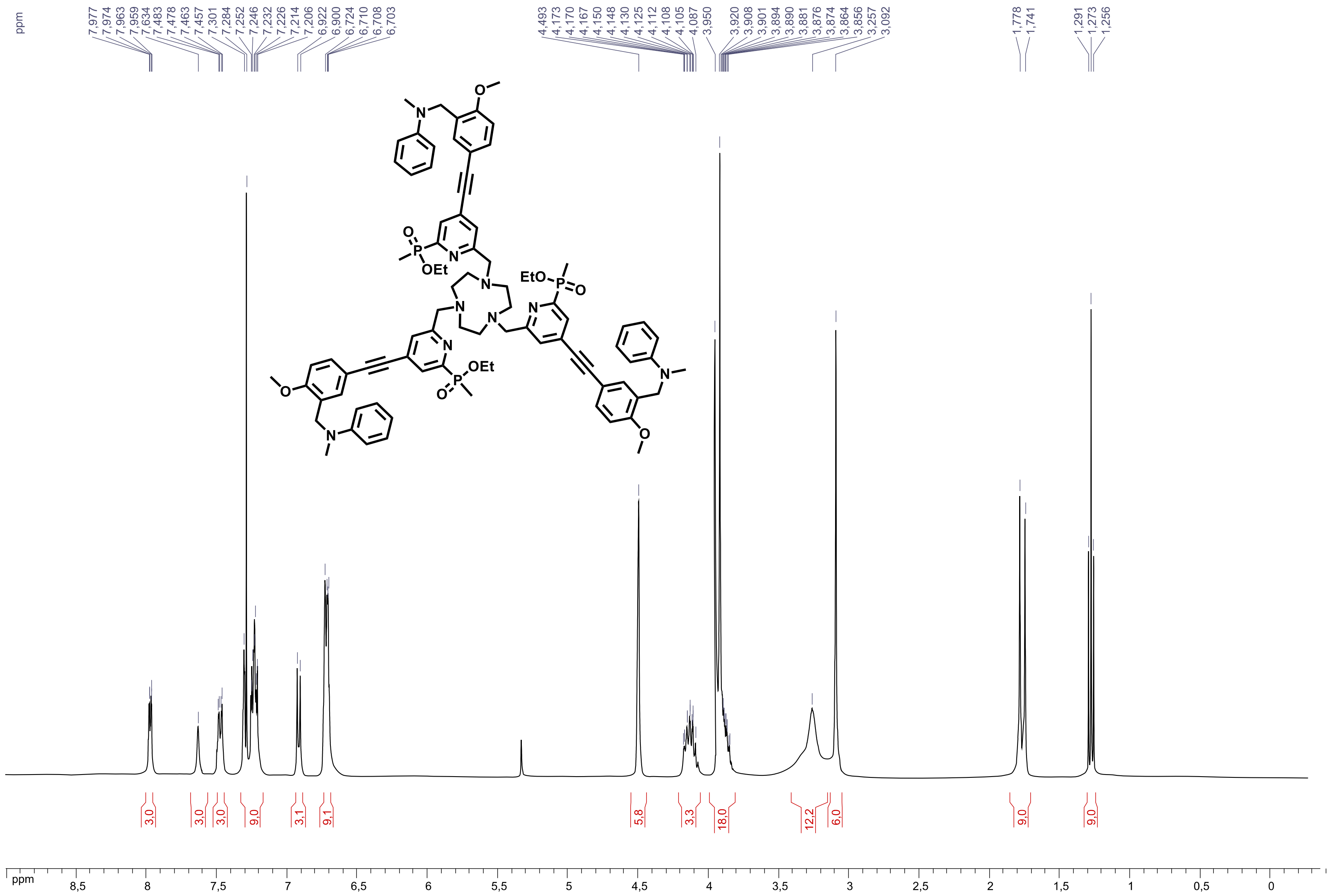

Figure S26. ^{1}H NMR ($CDCl_3$, 400 MHz) spectrum of compound **12**

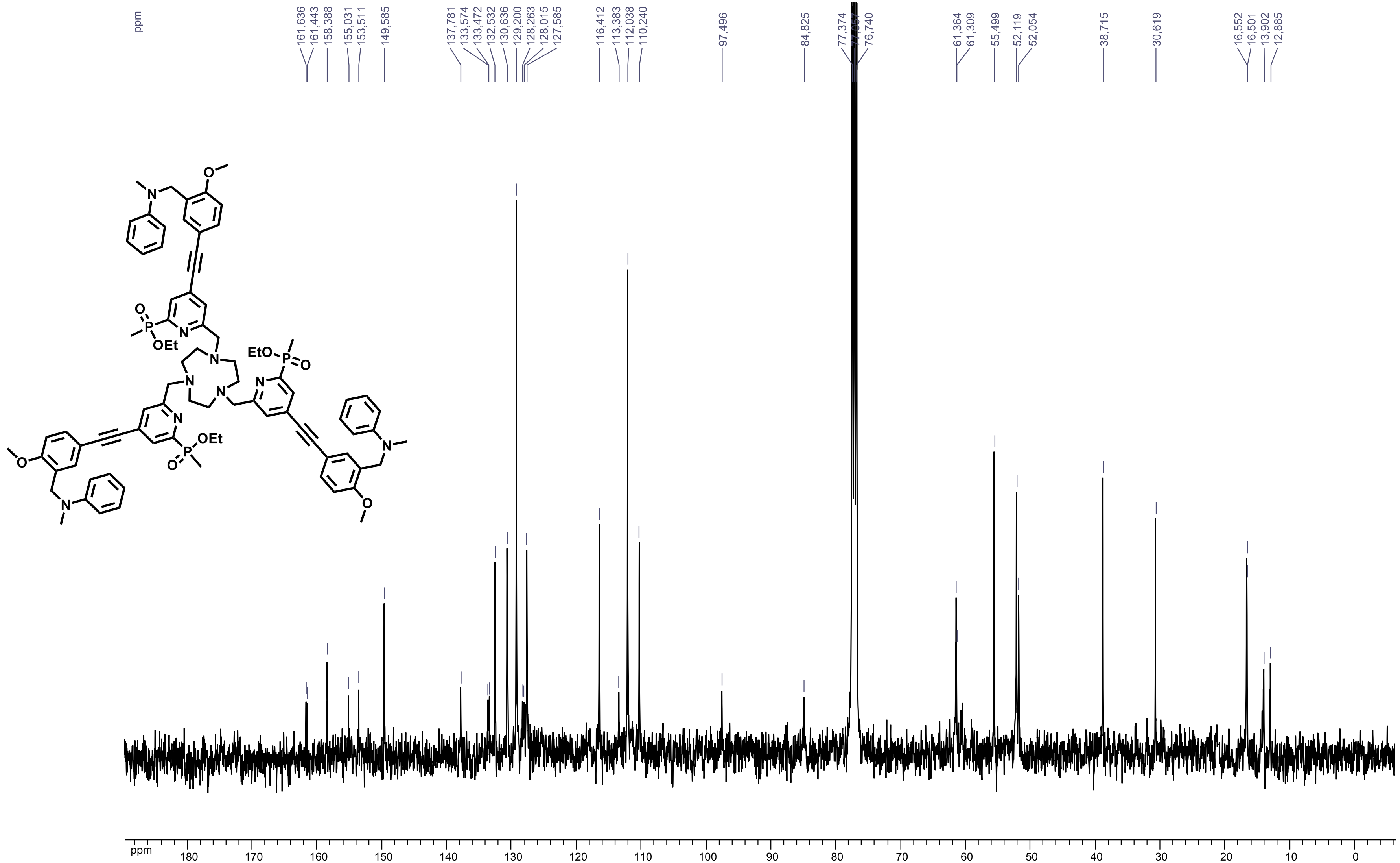

Figure S27. ^{13}C NMR ($CDCl_3$, 100 MHz) spectrum of compound **12**

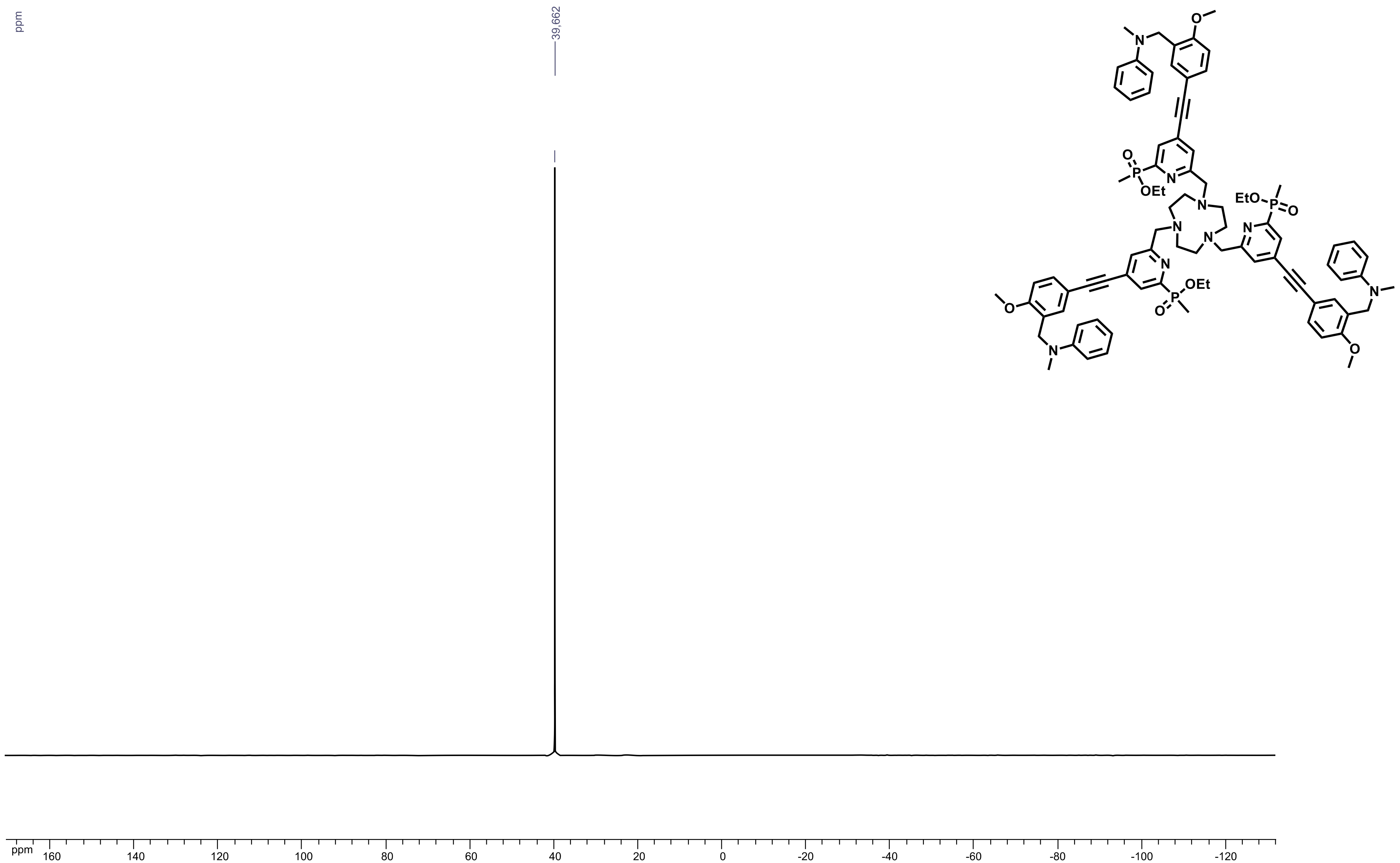

Figure S28. ^{31}P NMR ($CDCl_3$, 162 MHz) spectrum of compound **12**

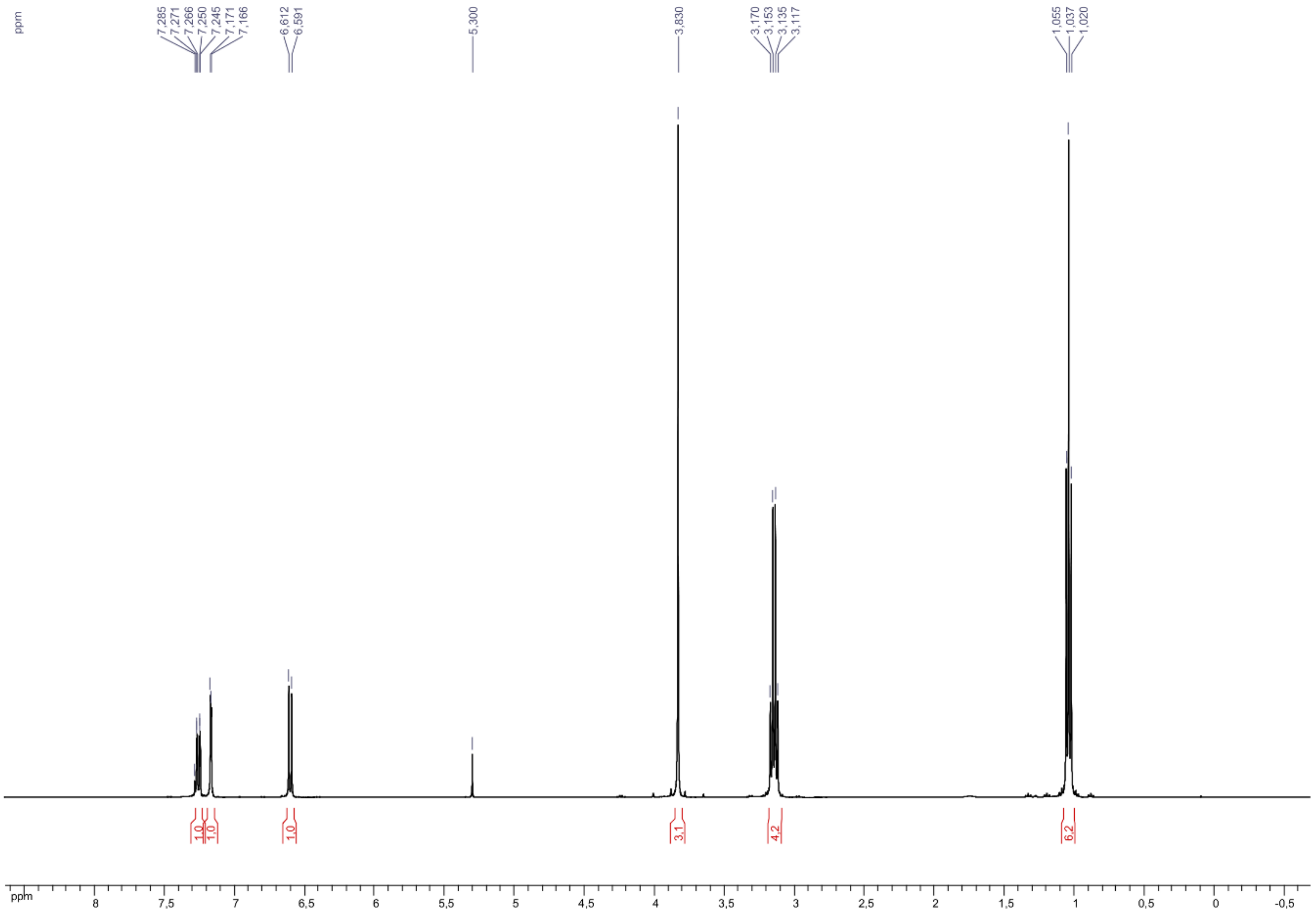

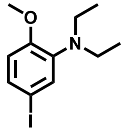

Figure S29. ^{1}H NMR ($CDCl_3$, 400 MHz) spectrum of compound **13**

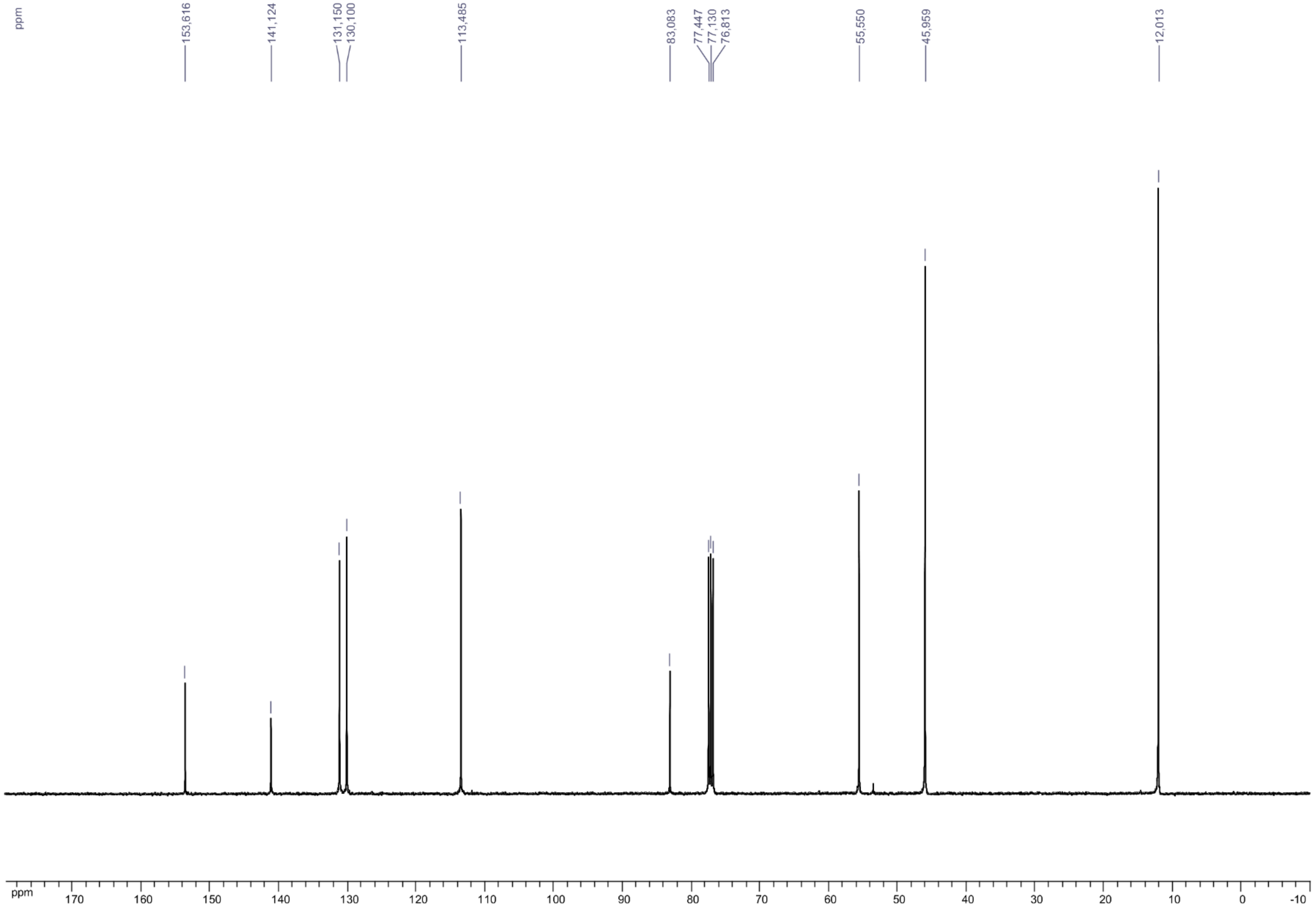

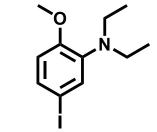

Figure S30. ^{13}C NMR ($CDCl_3$, 100 MHz) spectrum of compound **13**

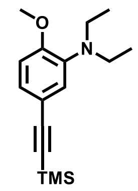

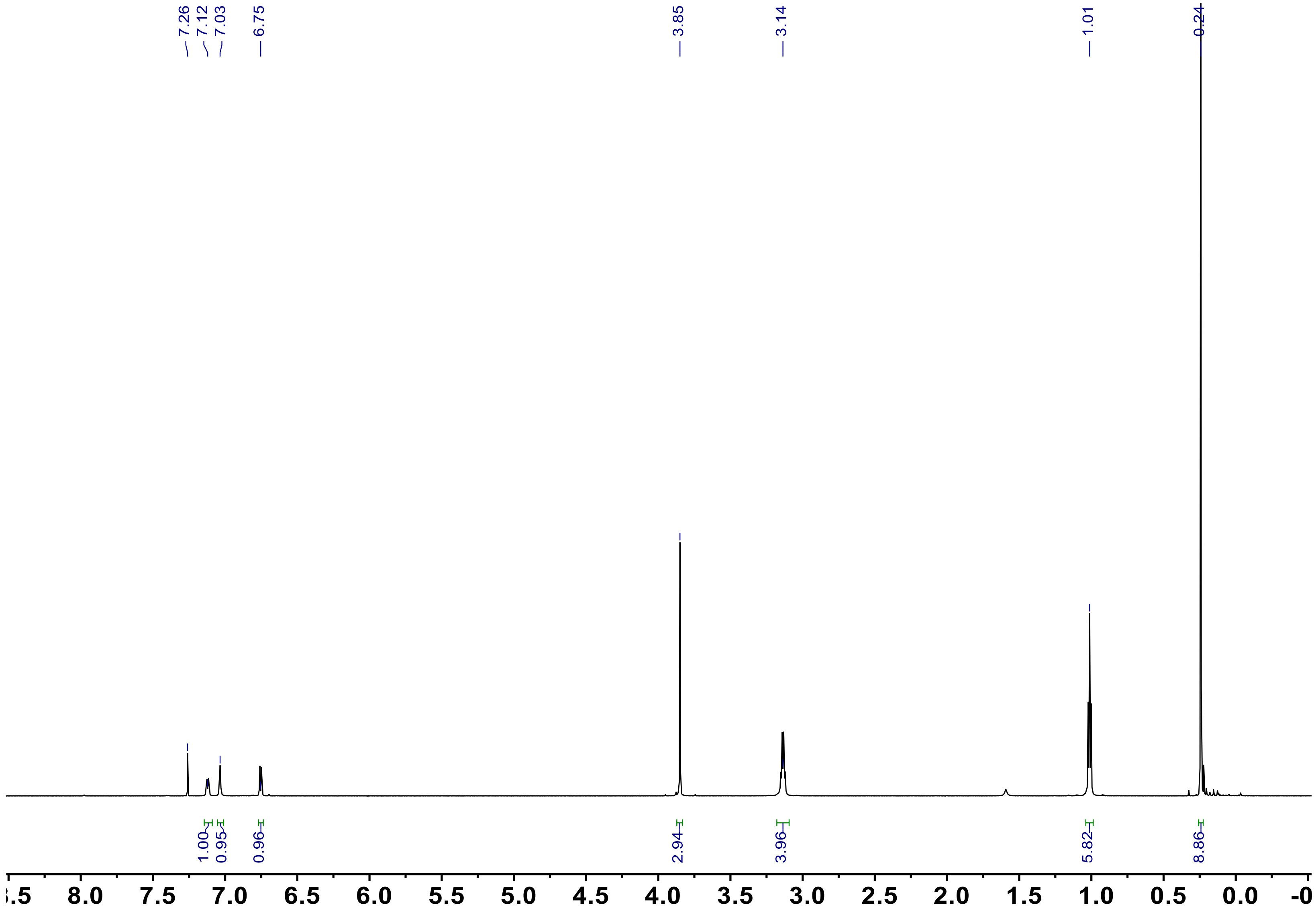

Figure S31. ^1H NMR ($CDCl_3$, 700 MHz) spectrum of compound **14**

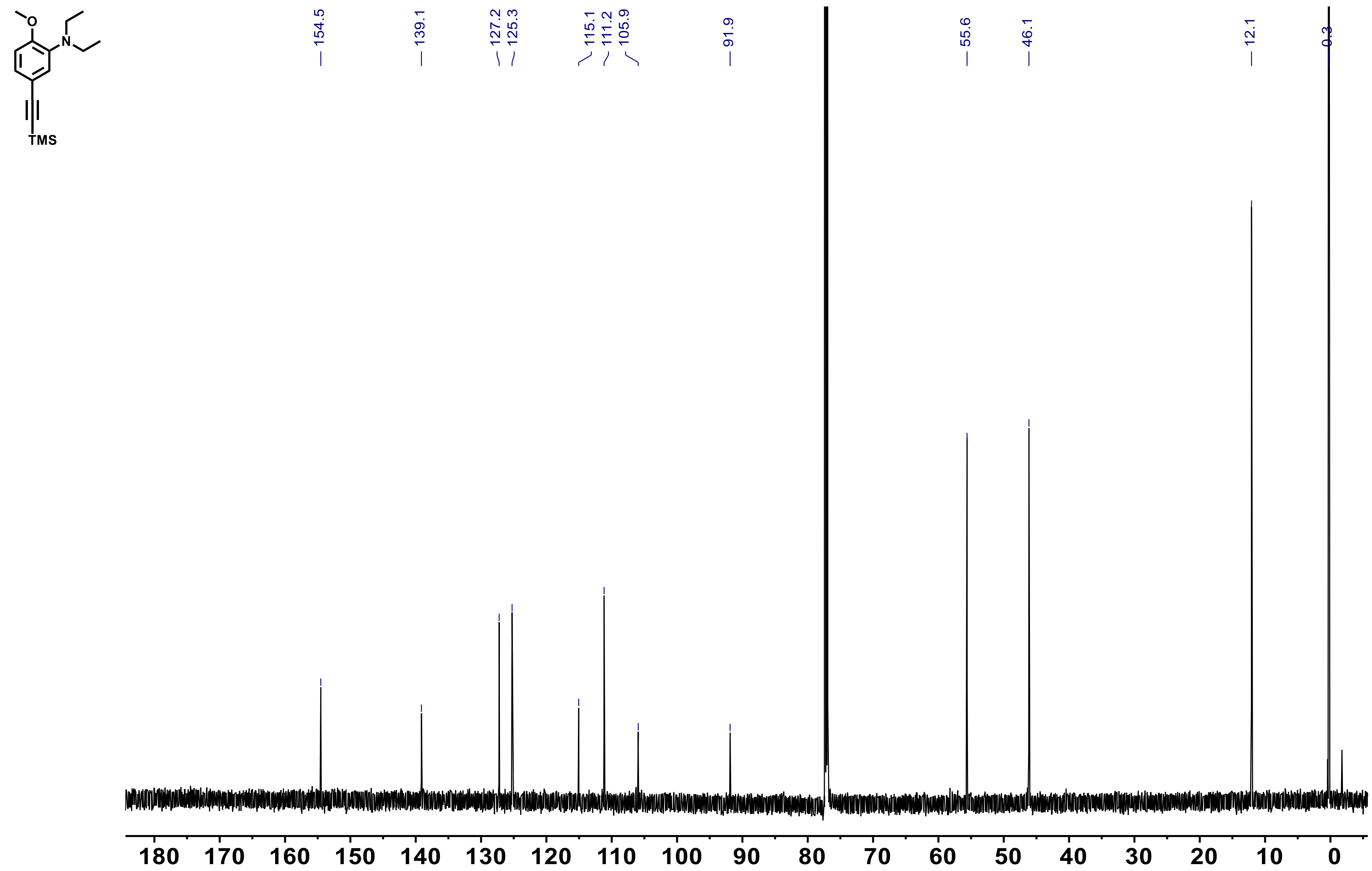

Figure S32. ^{13}C NMR ($CDCl_3$, 176 MHz) spectrum of compound **14**

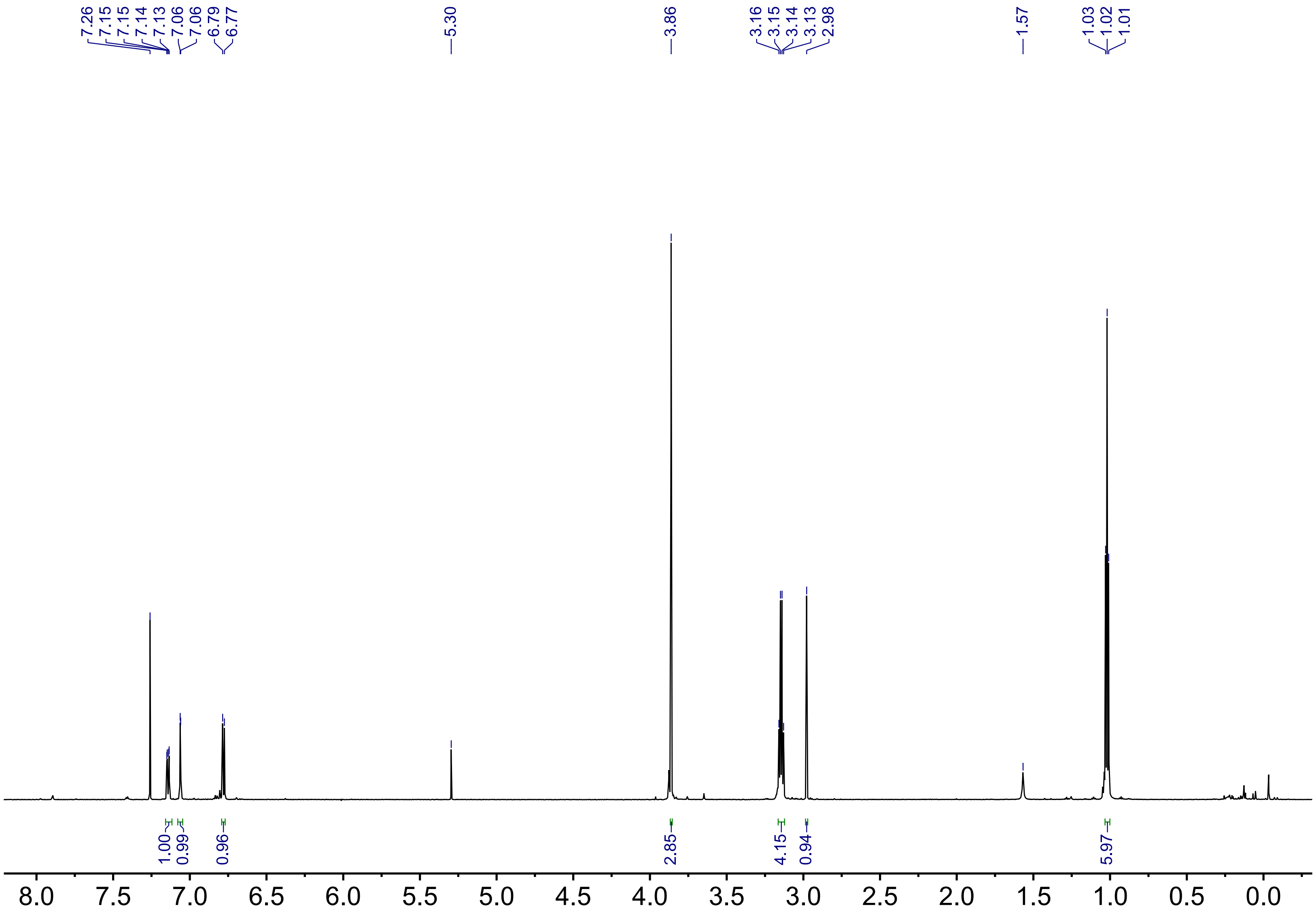

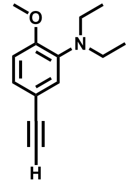

Figure S33. ^{1}H NMR ($CDCl_3$, 700 MHz) spectrum of compound **15**

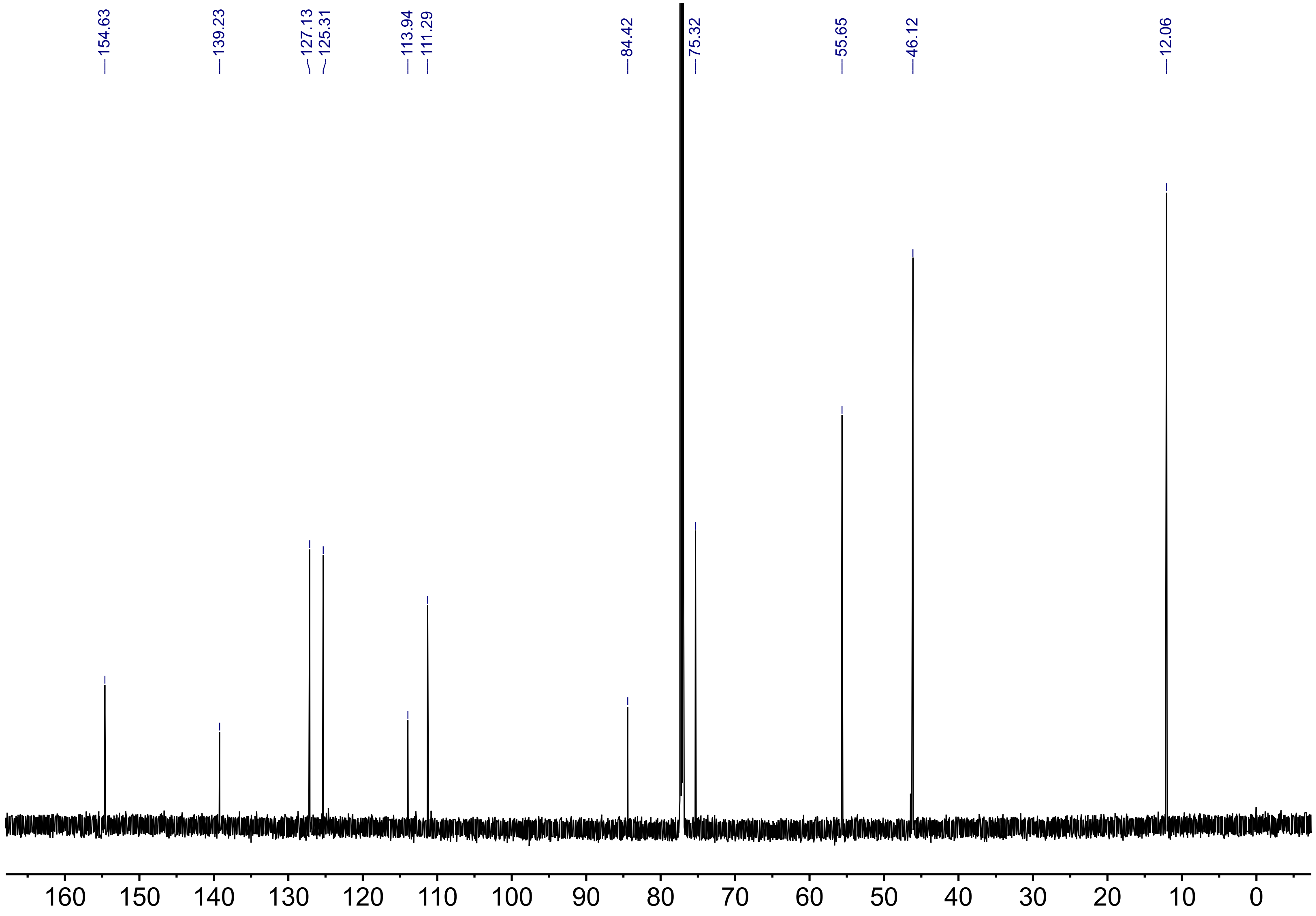

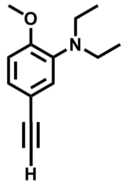

Figure S34. ^{13}C NMR ($CDCl_3$, 176 MHz) spectrum of compound **15**

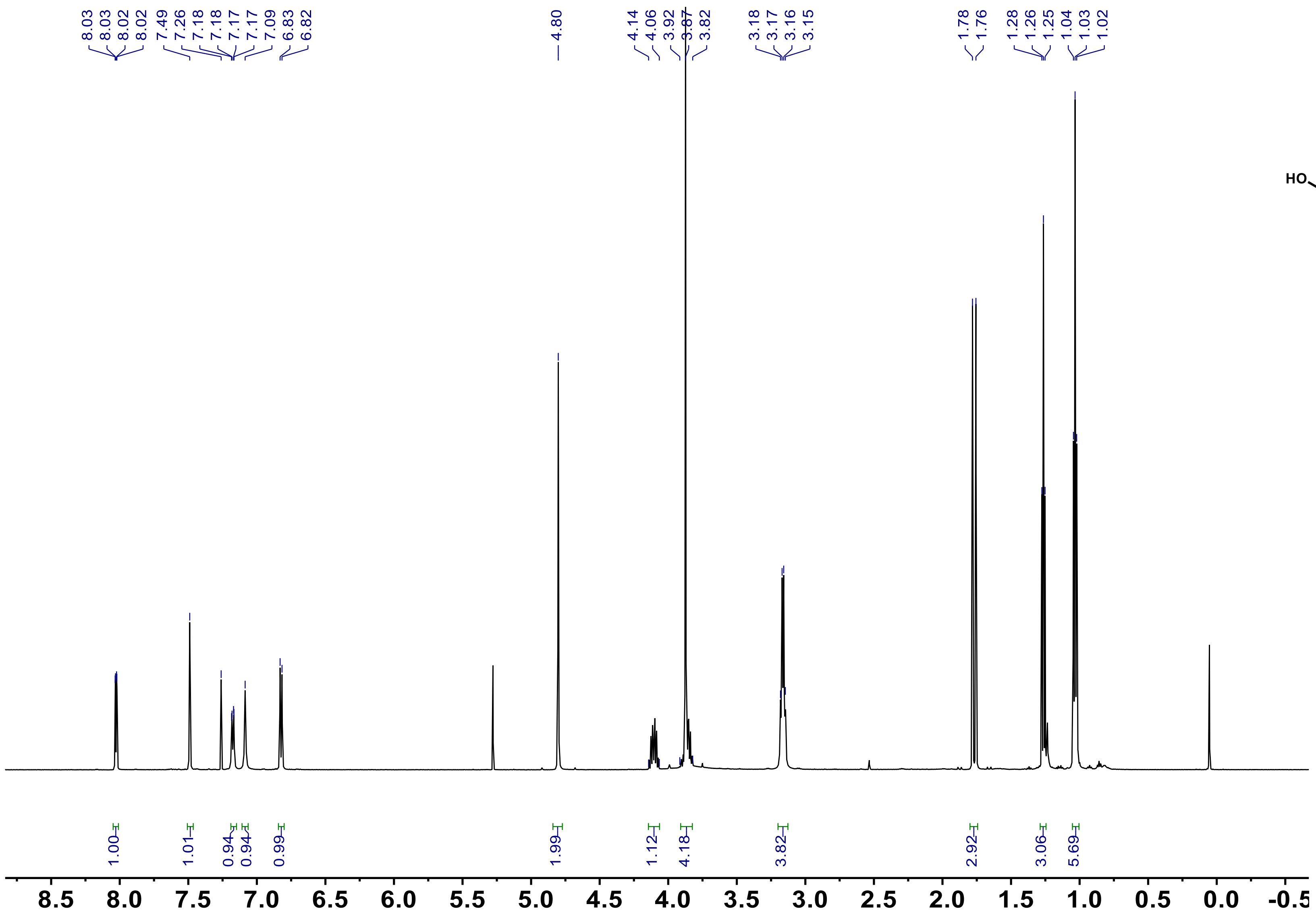

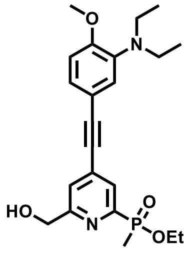

Figure S35. ^{1}H NMR ($CDCl_3$, 700 MHz) spectrum of compound **16**

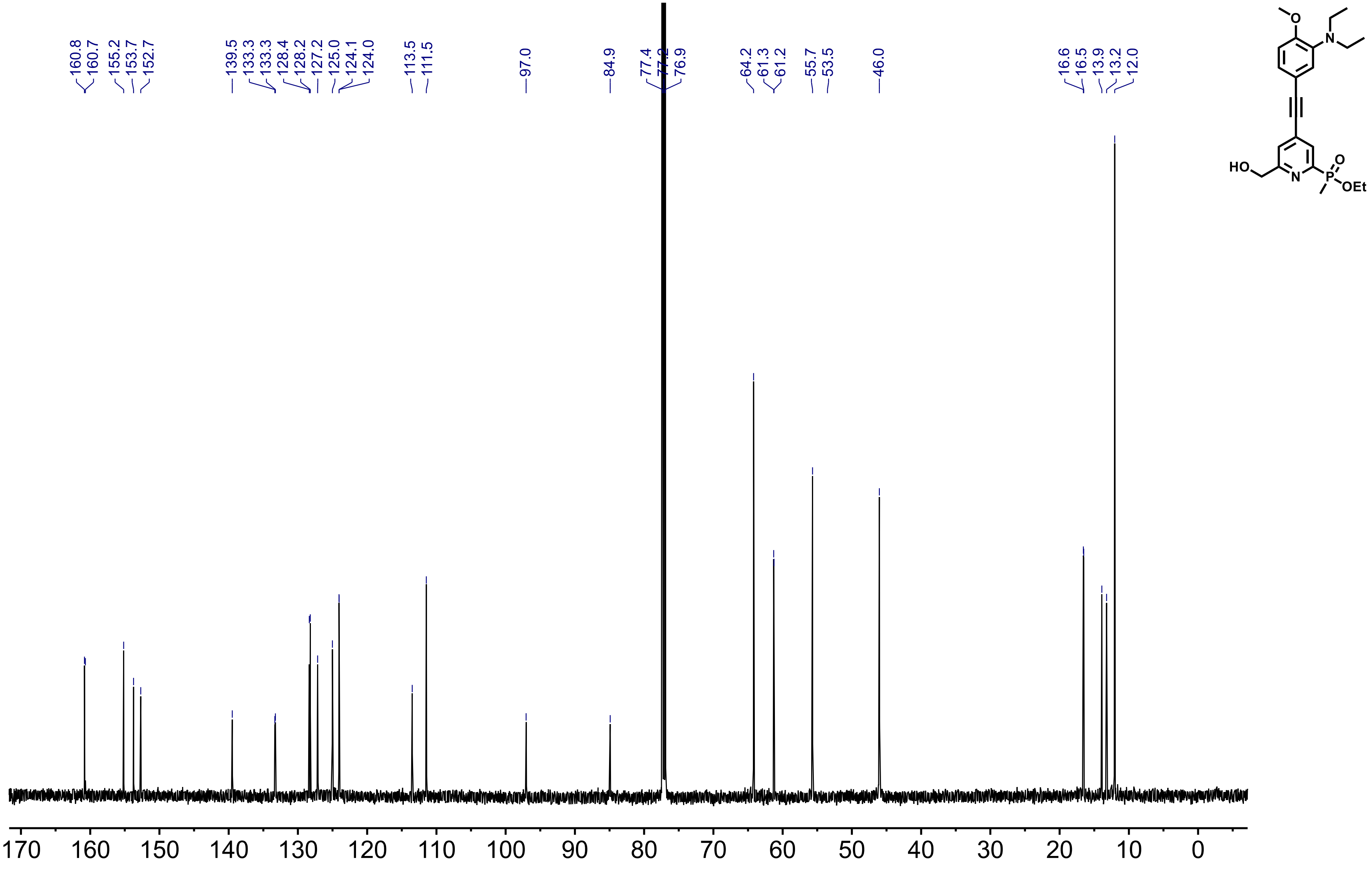

Figure S36. ^{13}C NMR ($CDCl_3$, 176 MHz) spectrum of compound **16**

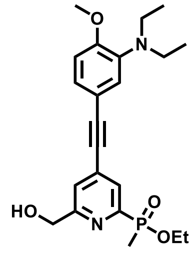

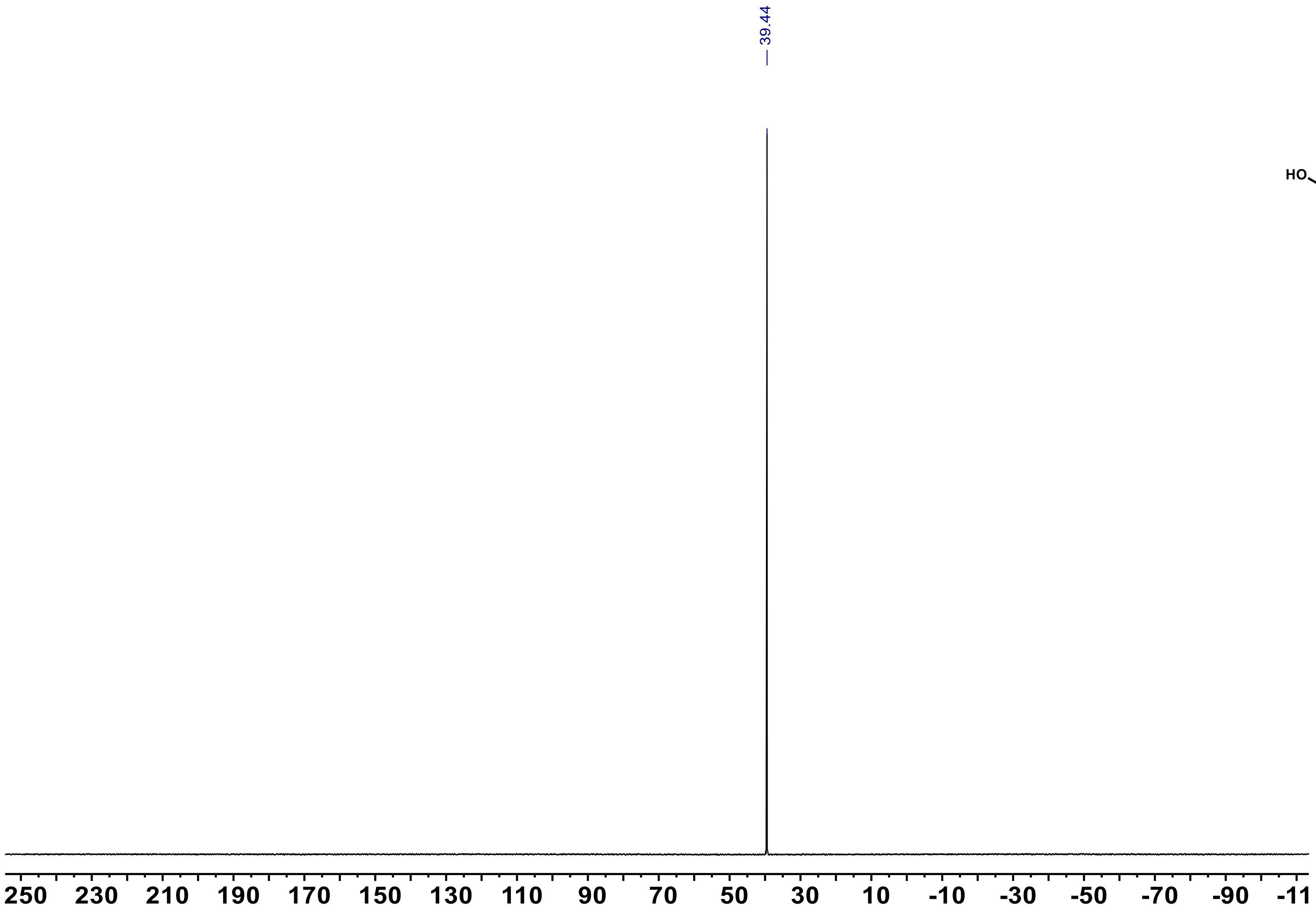

Figure S37. ^{31}P NMR ($CDCl_3$, 243 MHz) spectrum of compound **16**

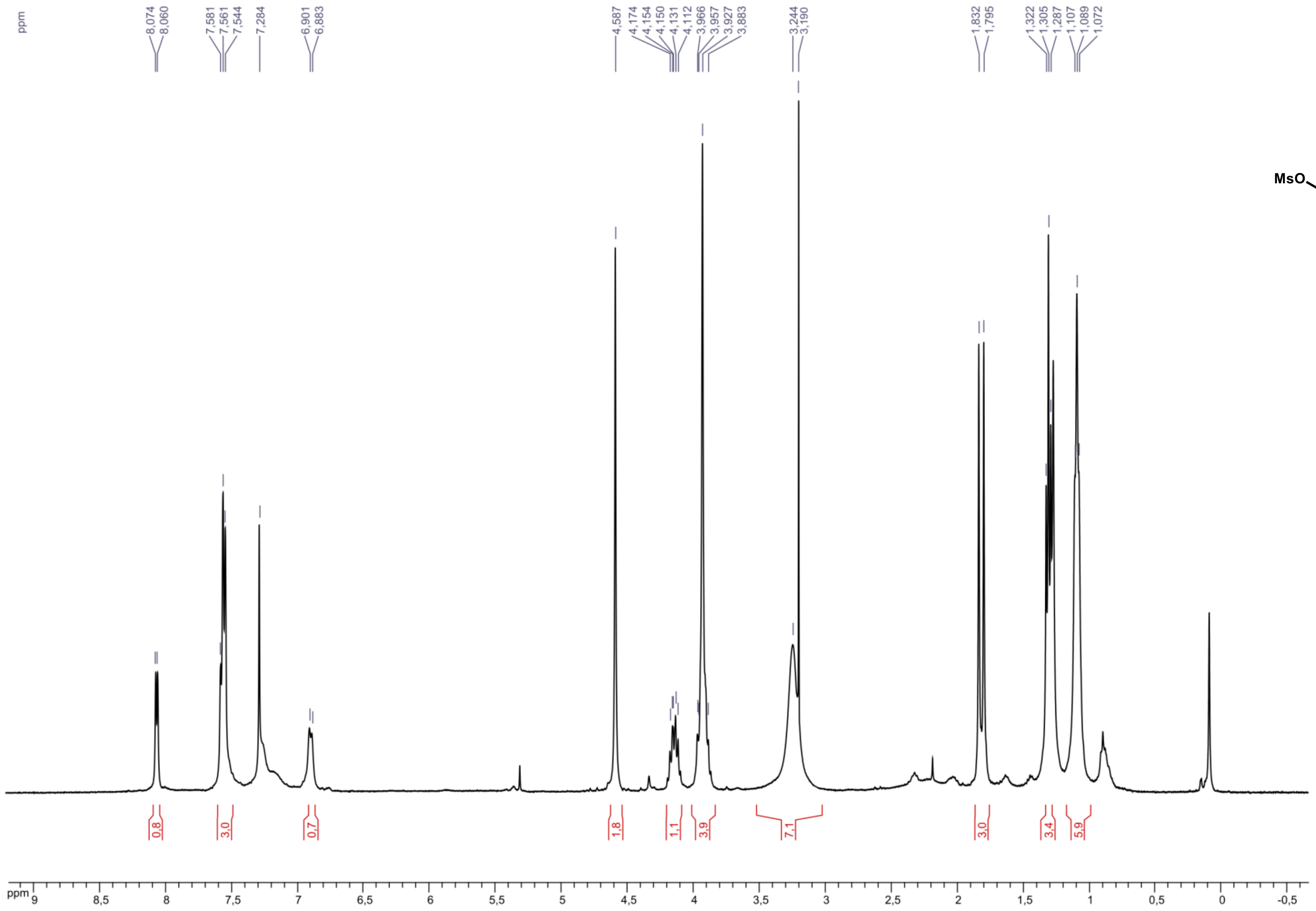

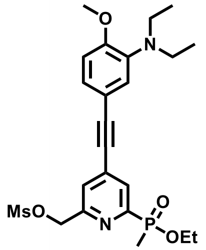

Figure S38. ^{1}H NMR ($CDCl_3$, 400 MHz) spectrum of compound **17**

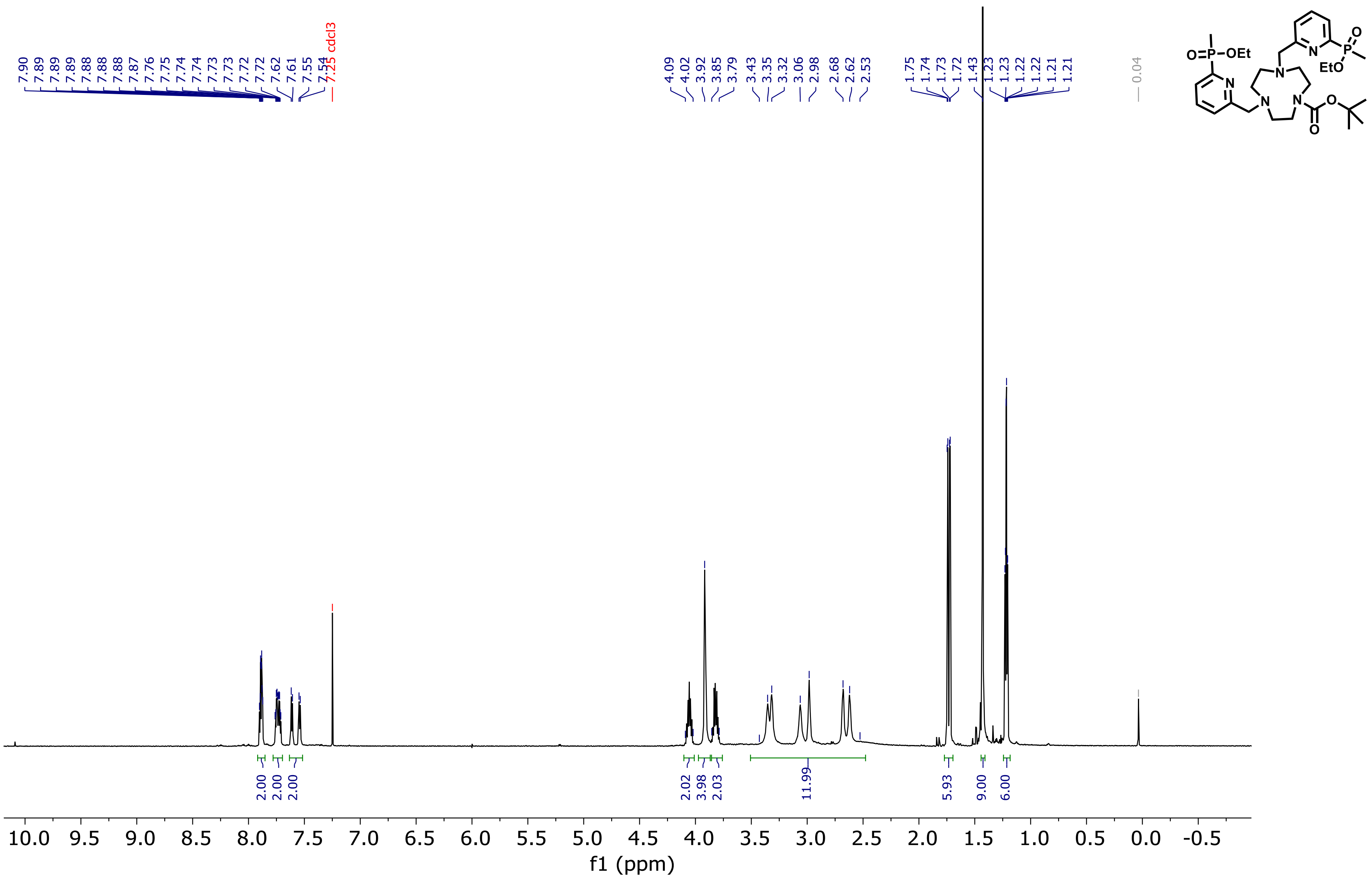

Figure S39. ^{1}H NMR ($CDCl_3$, 700 MHz) spectrum of compound **20**

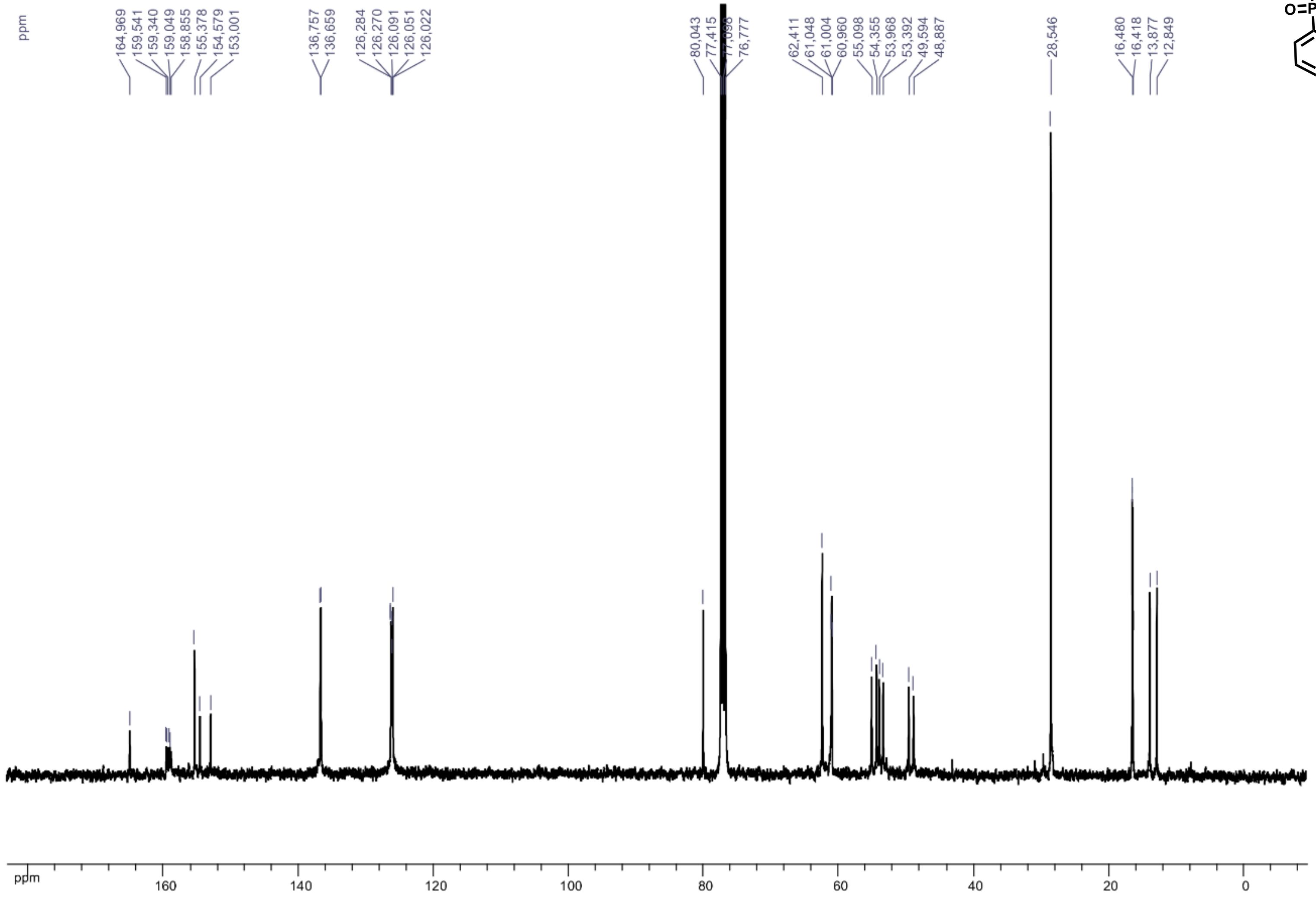

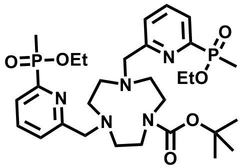

Figure S40. ^{13}C NMR ($CDCl_3$, 100 MHz) spectrum of compound **20**

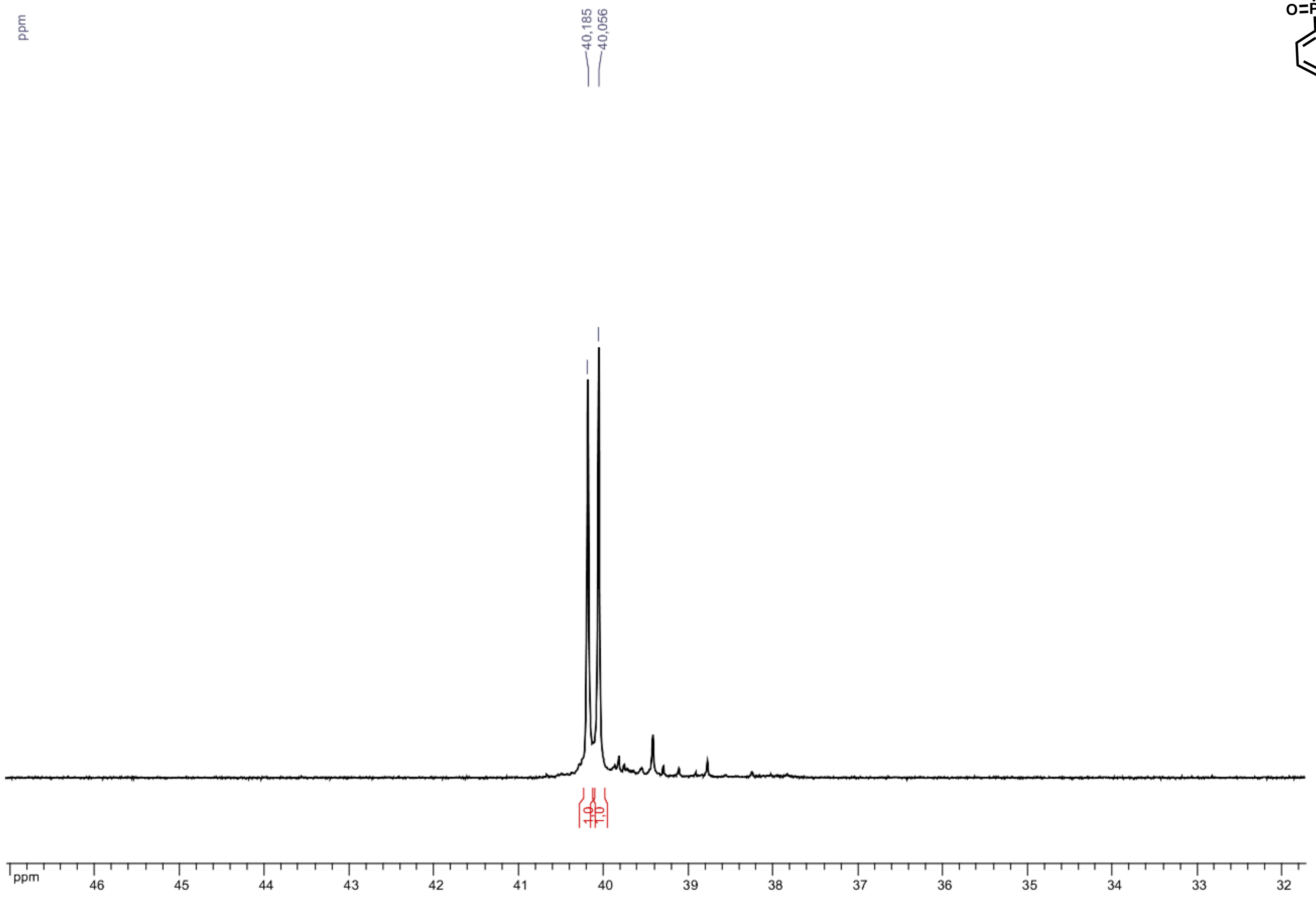

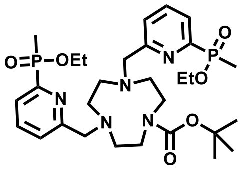

Figure S41. ^{31}P NMR ($CDCl_3$, 162 MHz) spectrum of compound **20**

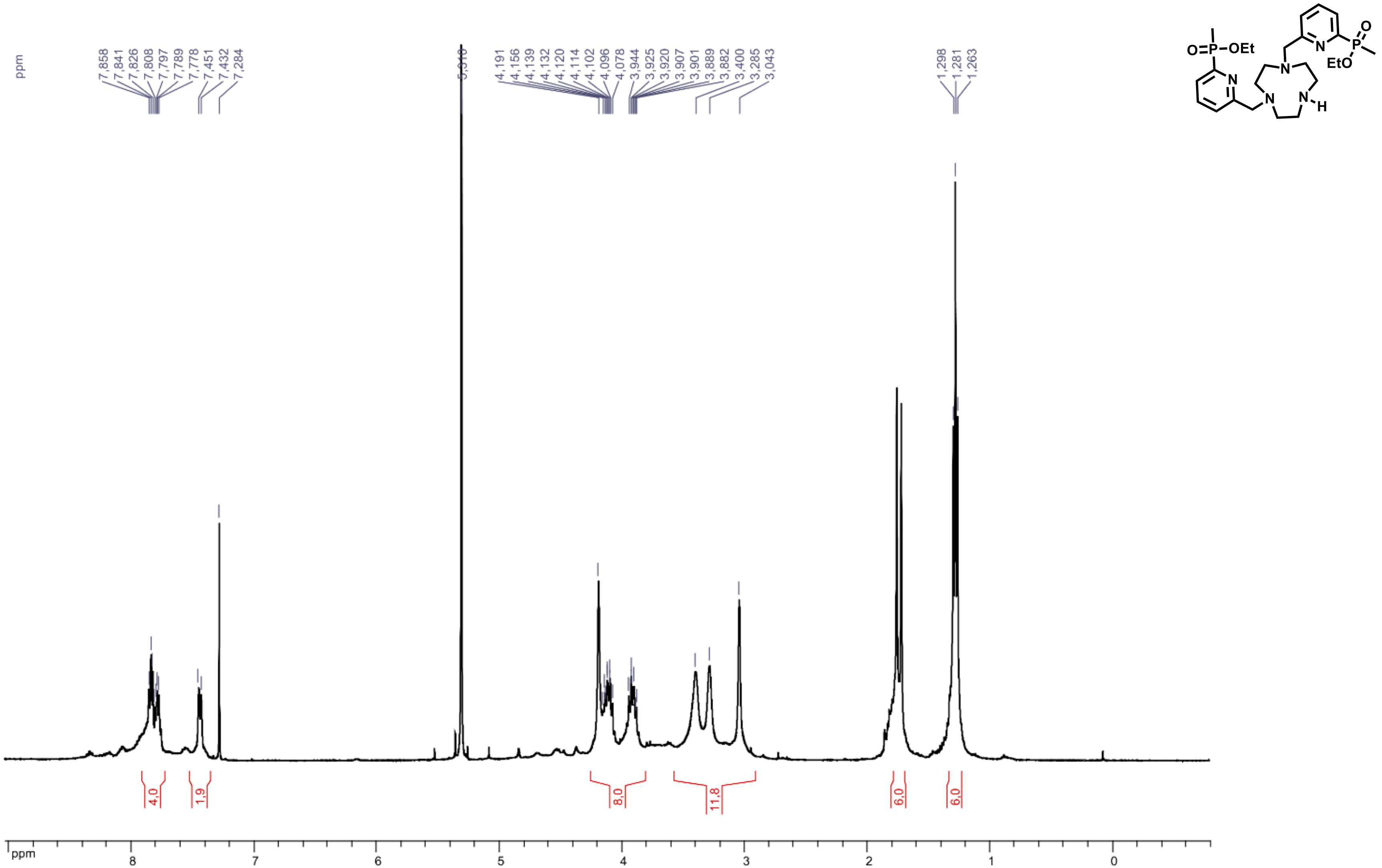

Figure S42. ^{1}H NMR ($CDCl_3$, 400 MHz) spectrum of compound **21**

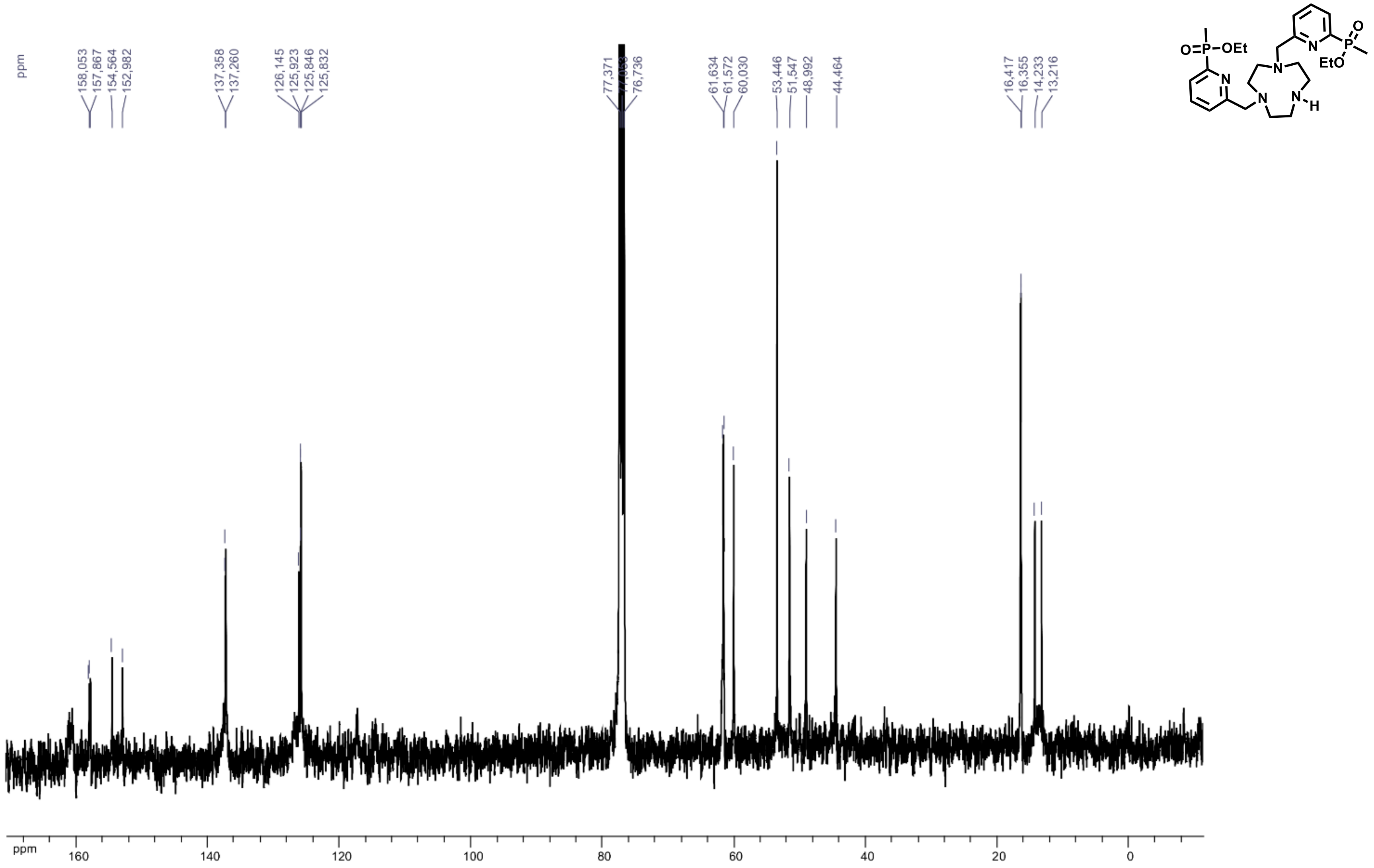

Figure S43. ^{13}C NMR ($CDCl_3$, 100 MHz) spectrum of compound **21**

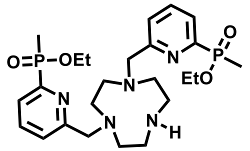

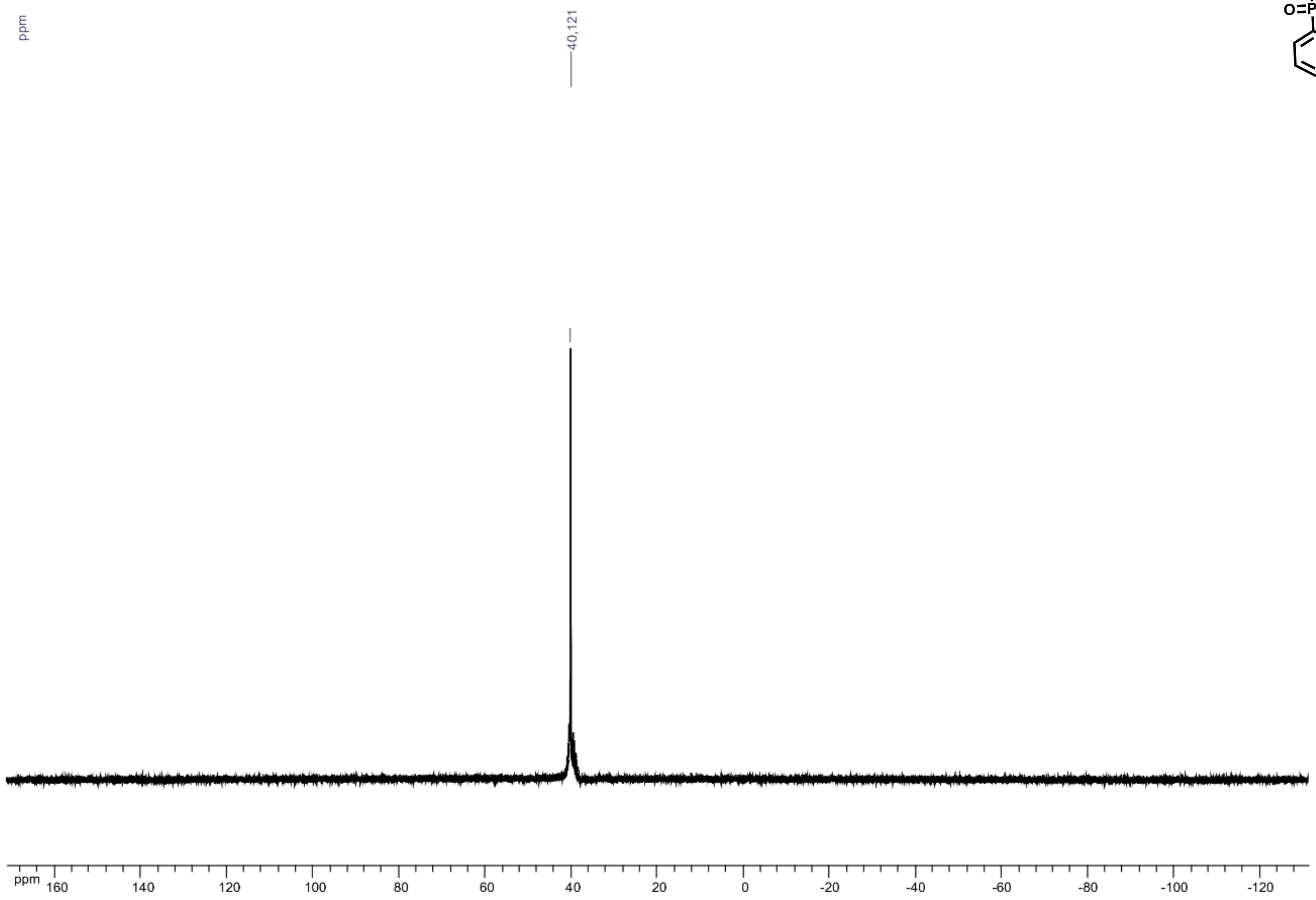

Figure S44. ^{31}P NMR ($CDCl_3$, 162 MHz) spectrum of compound **21**

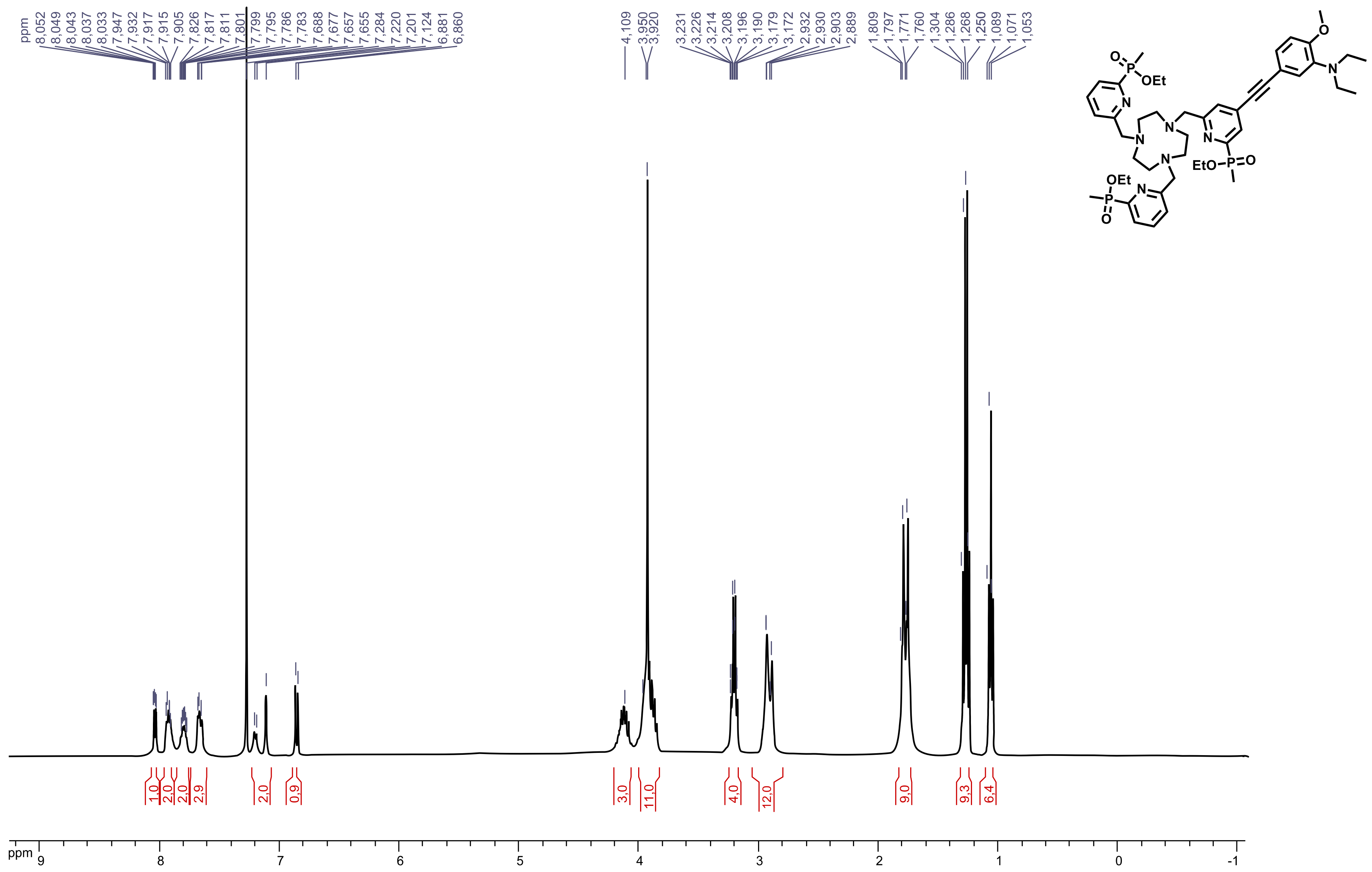

Figure S45. ^{1}H NMR ($CDCl_3$, 400 MHz) spectrum of compound **22**

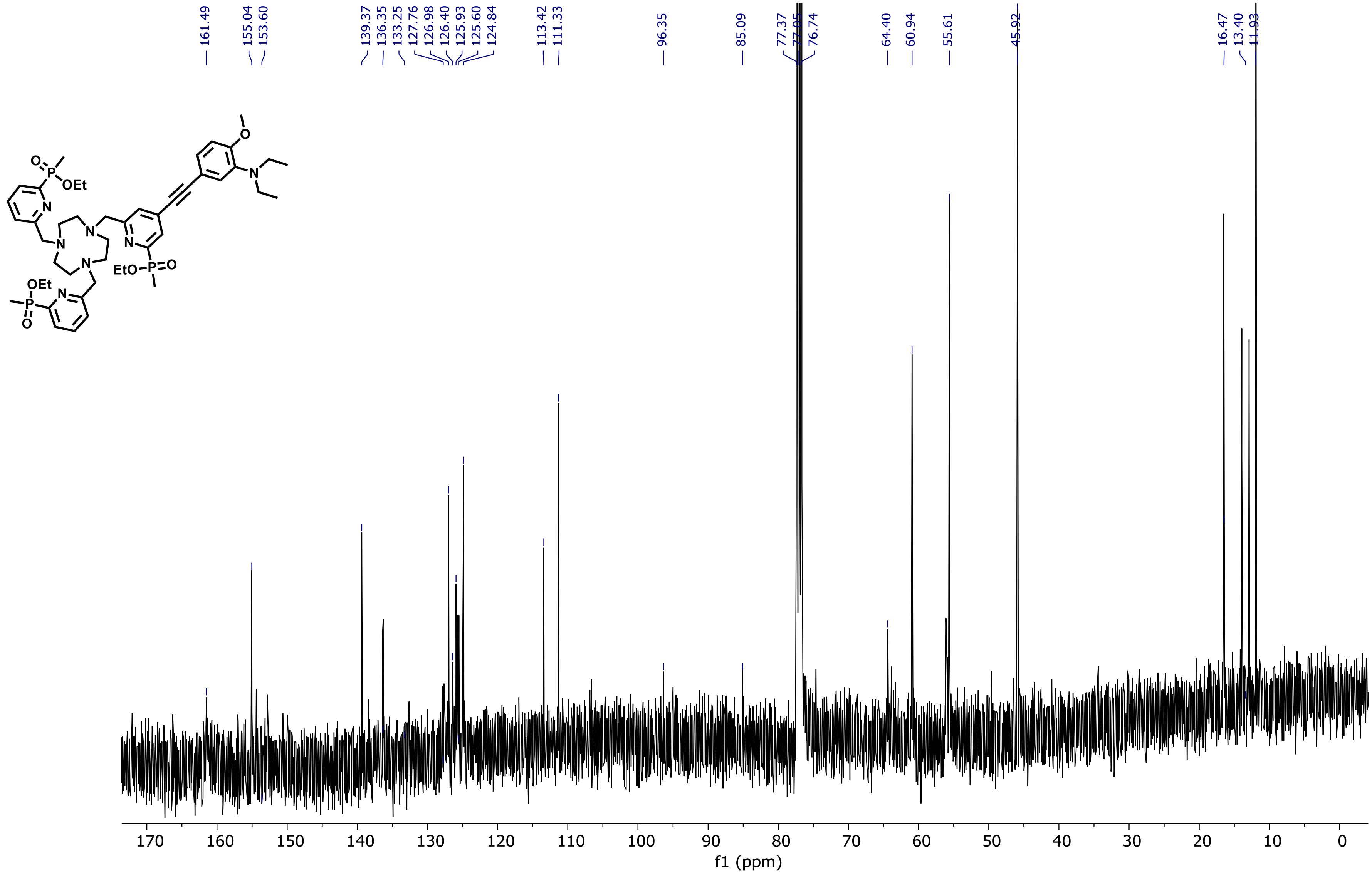

Figure S46. ^{13}C NMR ($CDCl_3$, 100 MHz) spectrum of compound **22**

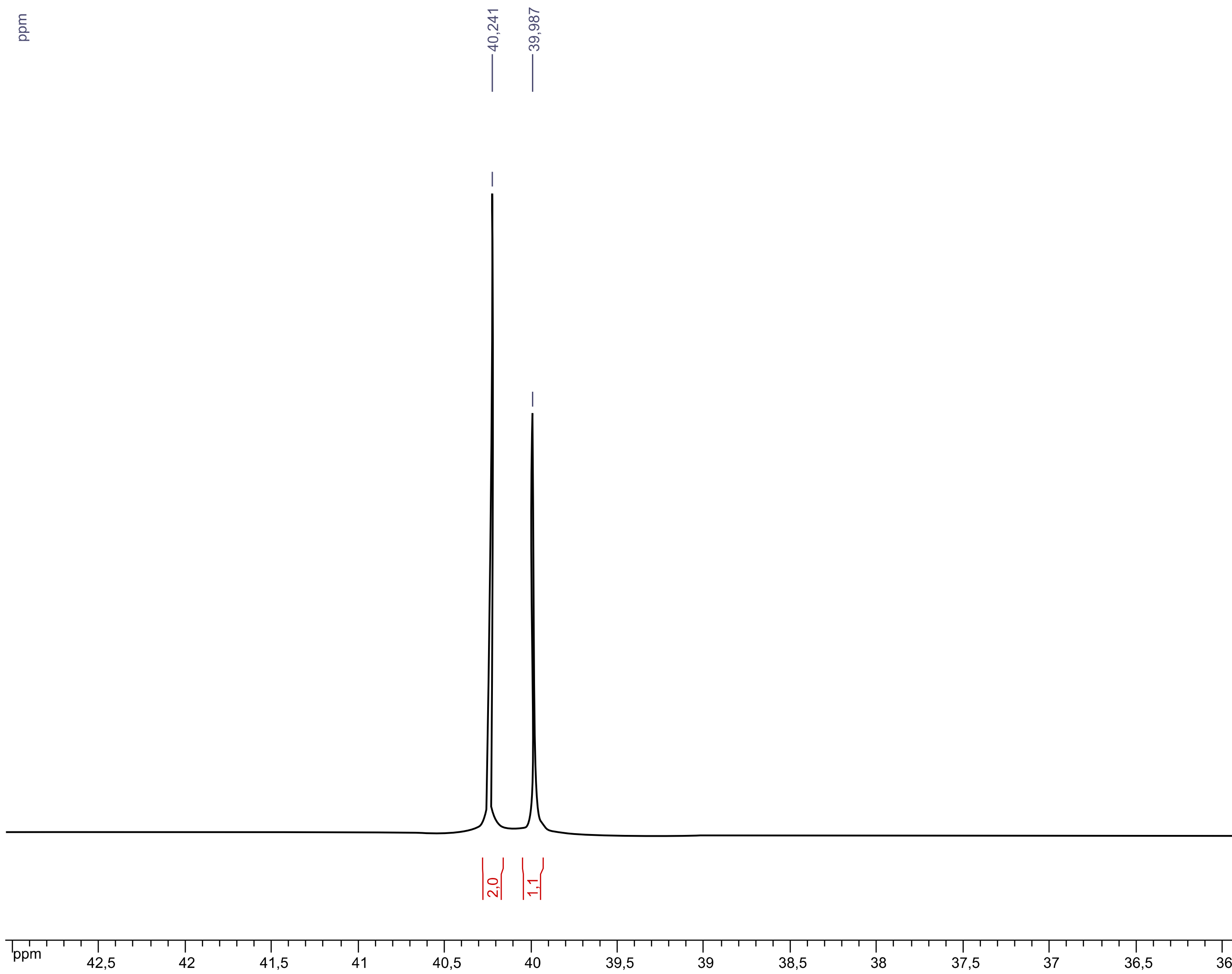

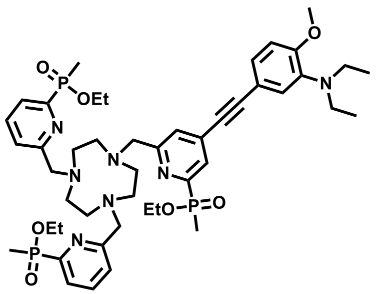

Figure S47. ^{31}P NMR ($CDCl_3$, 162 MHz) spectrum of compound **22**

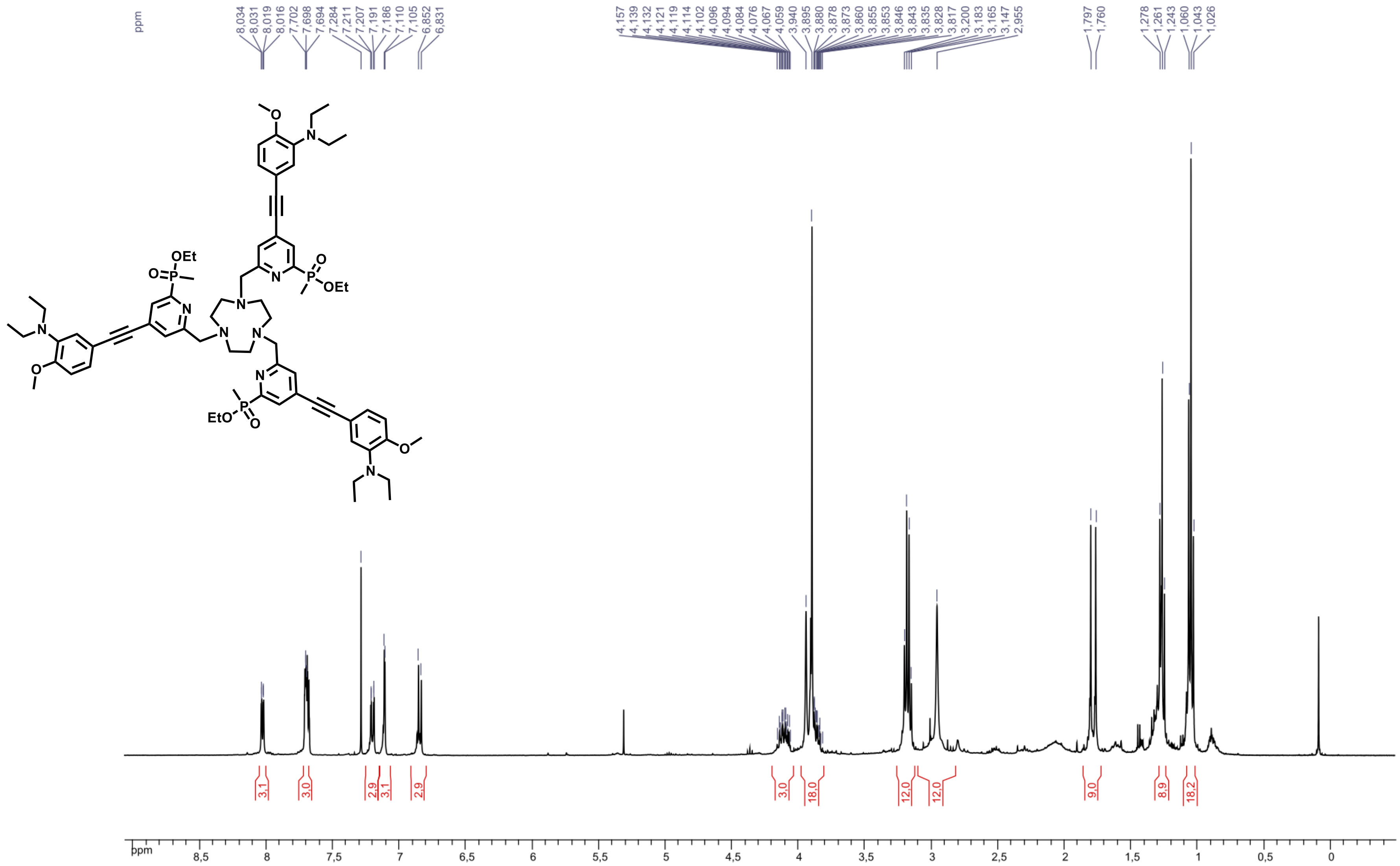

Figure S48. ^{1}H NMR ($CDCl_3$, 400 MHz) spectrum of compound **23**

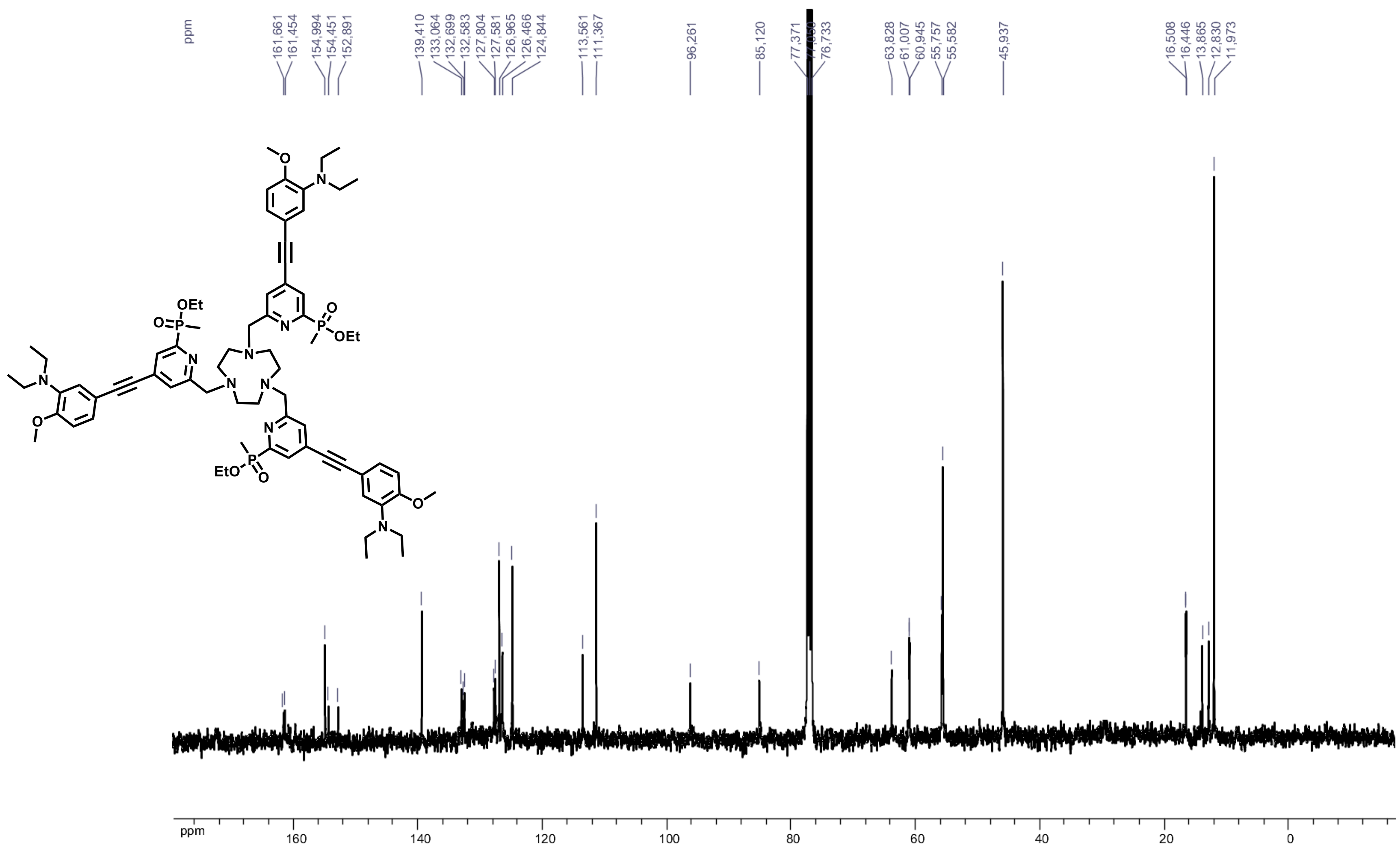

Figure S49. ^{13}C NMR ($CDCl_3$, 100 MHz) spectrum of compound **23**

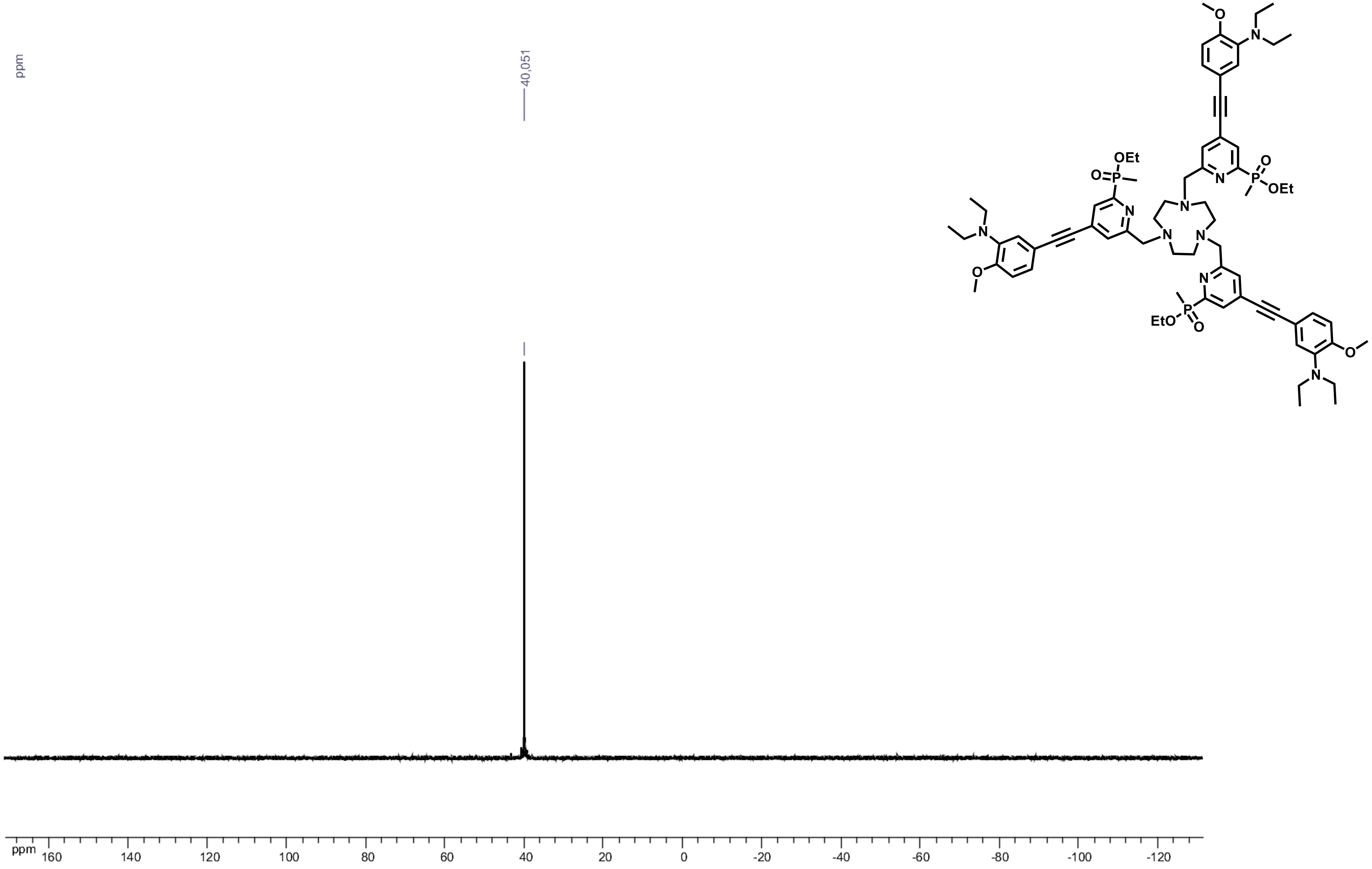

Figure S50. ^{31}P NMR ($CDCl_3$, 162 MHz) spectrum of compound **23**

GC and HPLC Traces

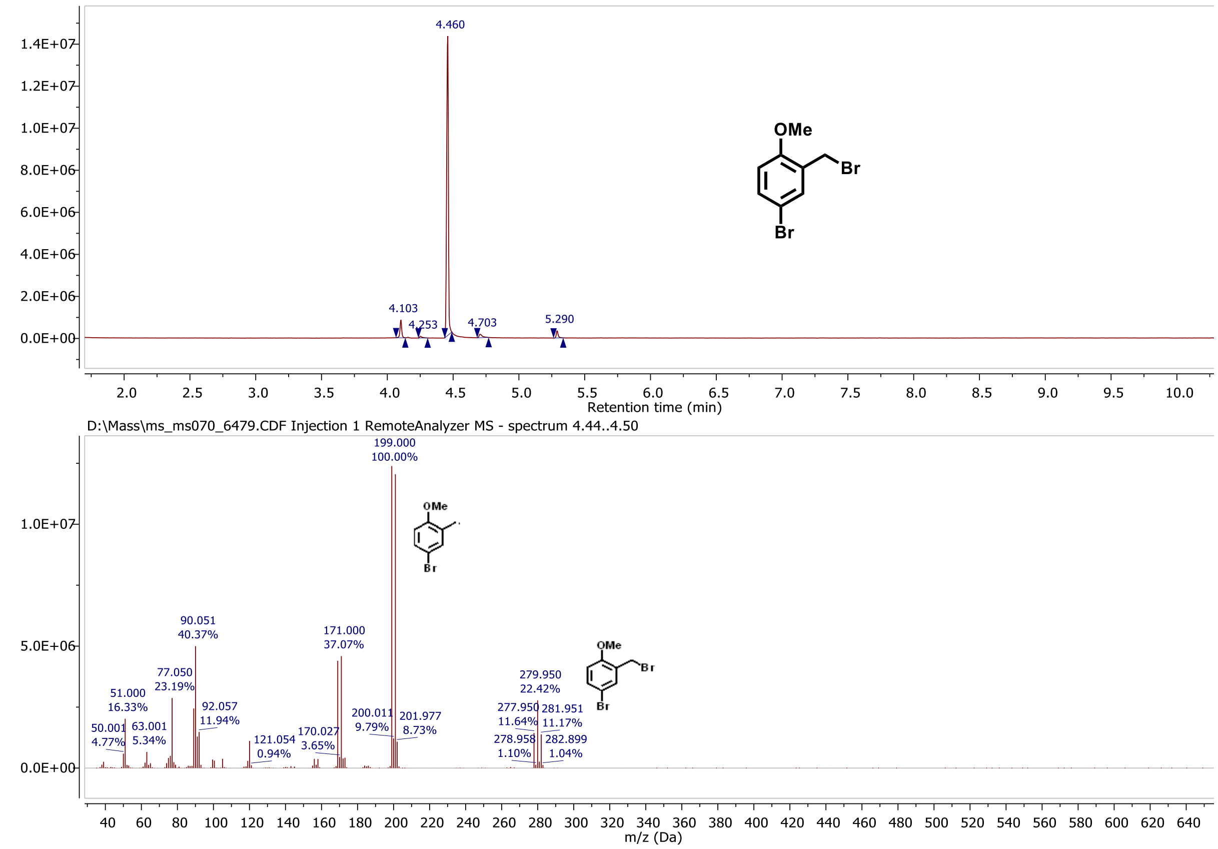

Figure S51. GC/MS traces of compound **1**

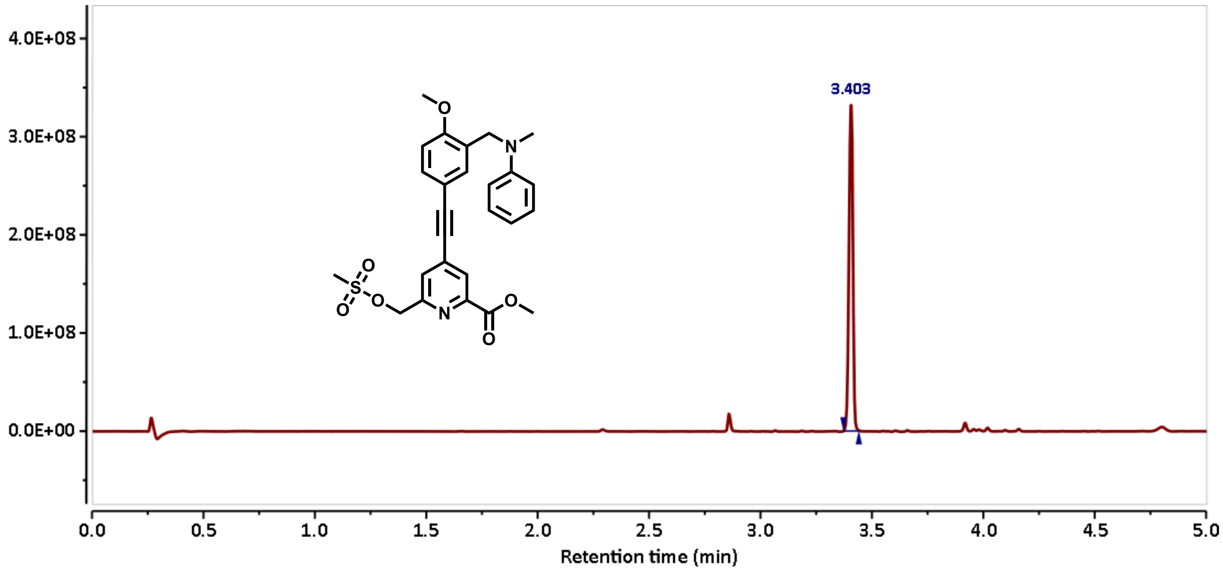

Figure S52. HPLC trace of compound **7**

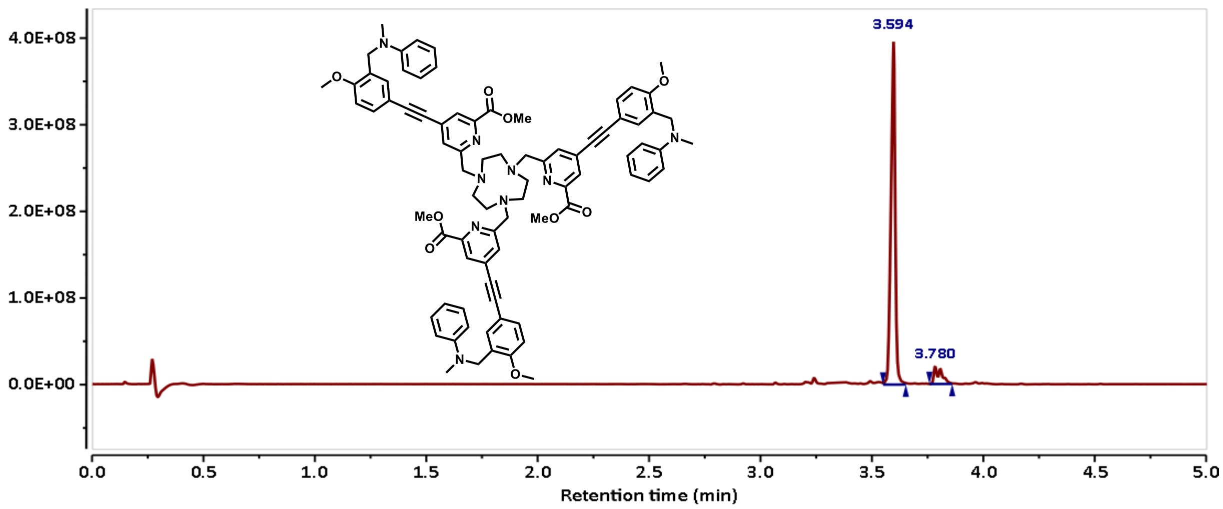

Figure S53. HPLC trace of compound **8**

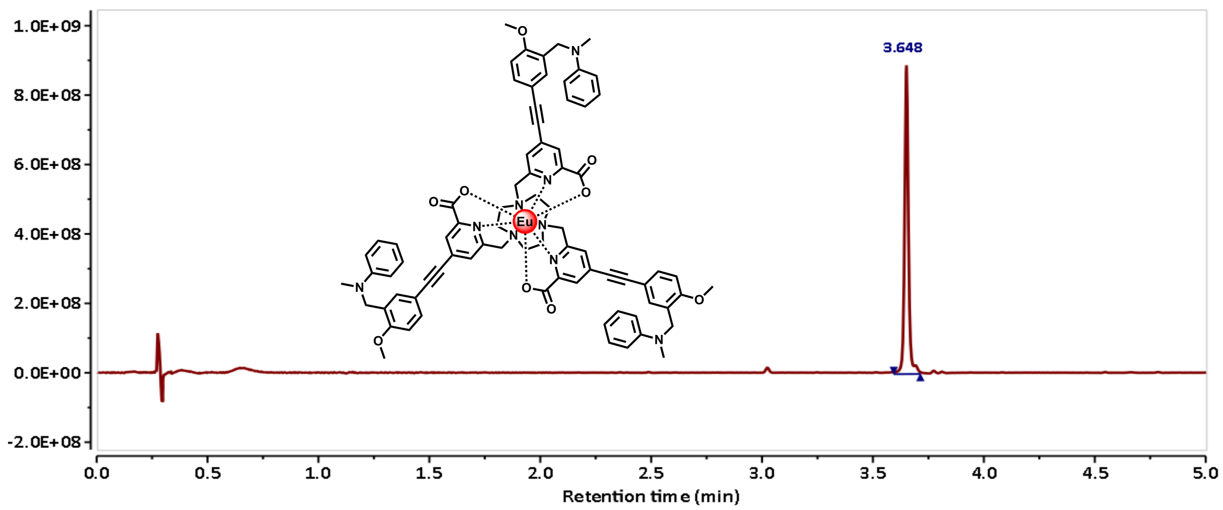

Figure S54. HPLC trace of complex **[Eu*L*[1]]**

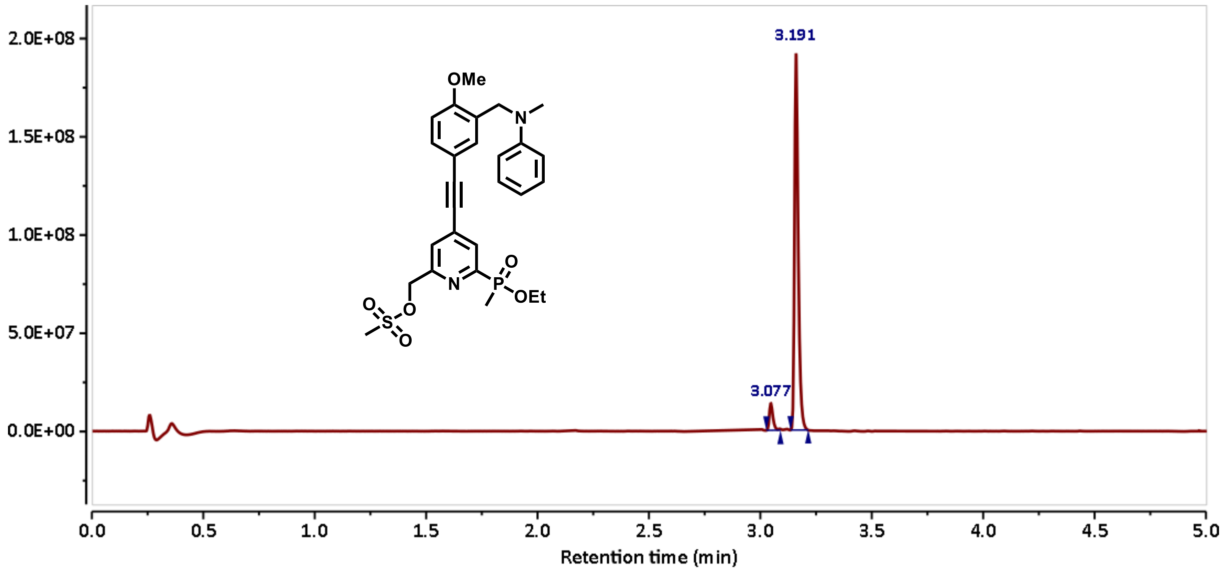

Figure S55. HPLC trace of compound **11**

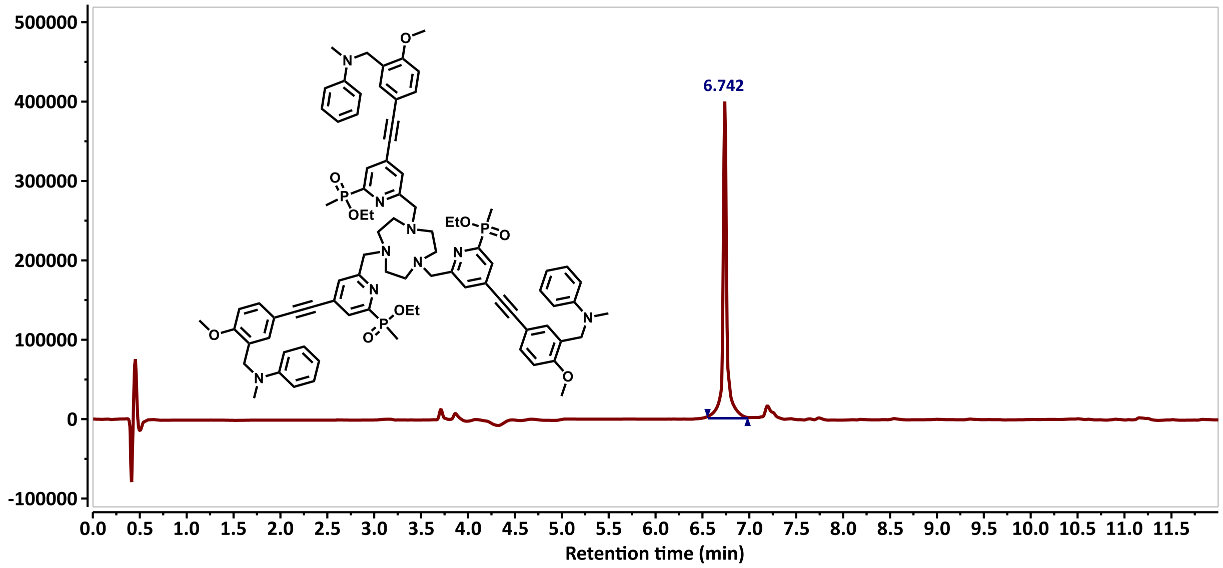

Figure S56. HPLC trace of compound **12**

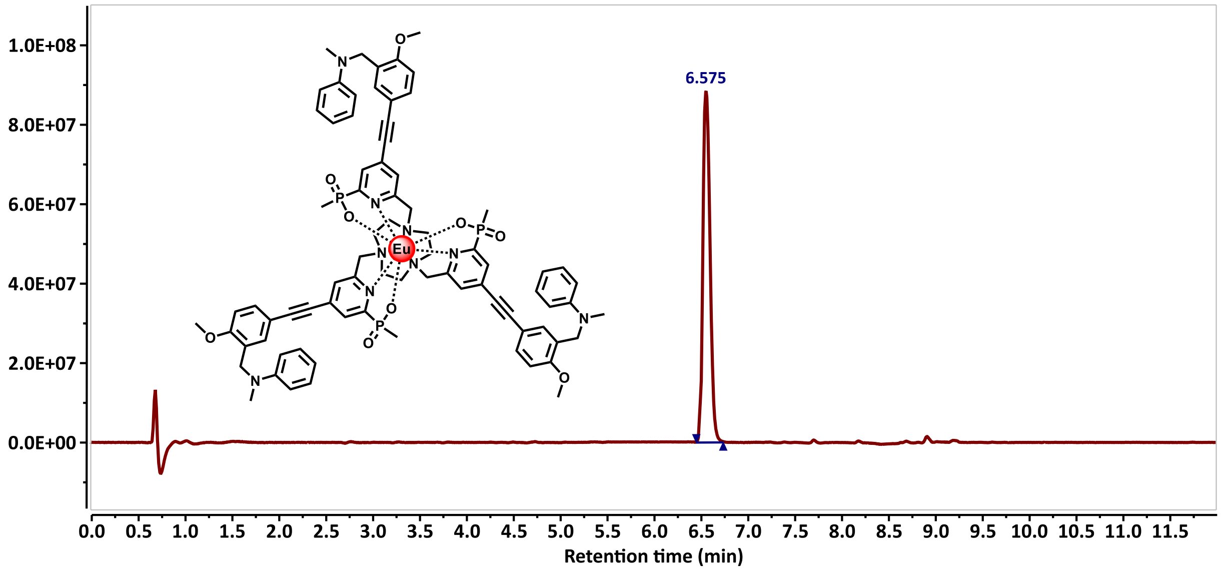

Figure S57. HPLC trace of complex **[Eu*L*²]**

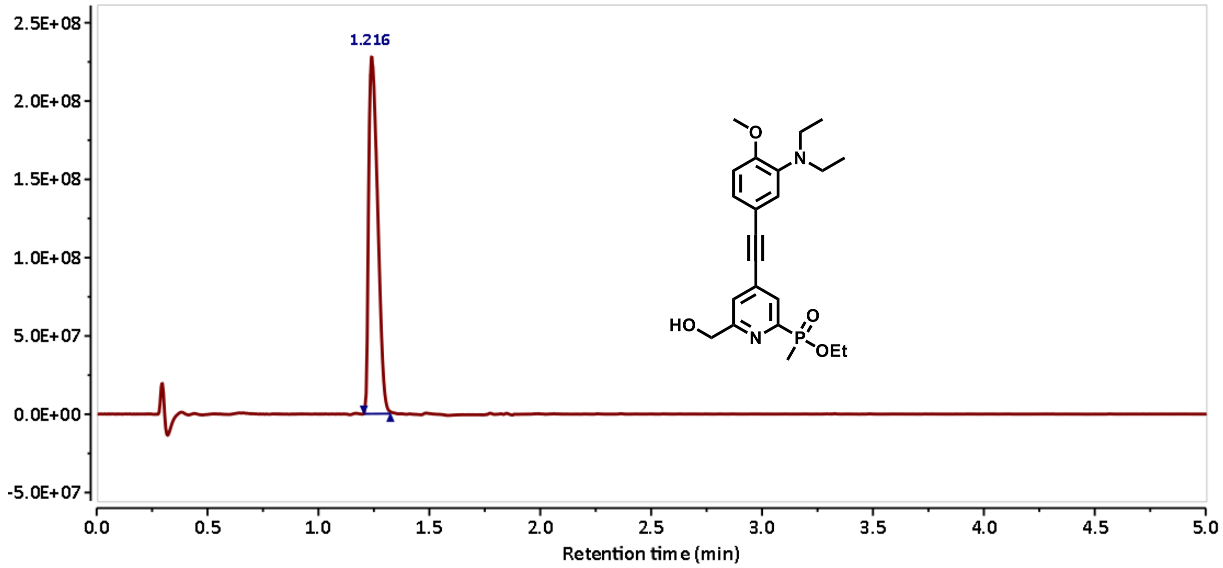

Figure S58. HPLC trace of compound **16**

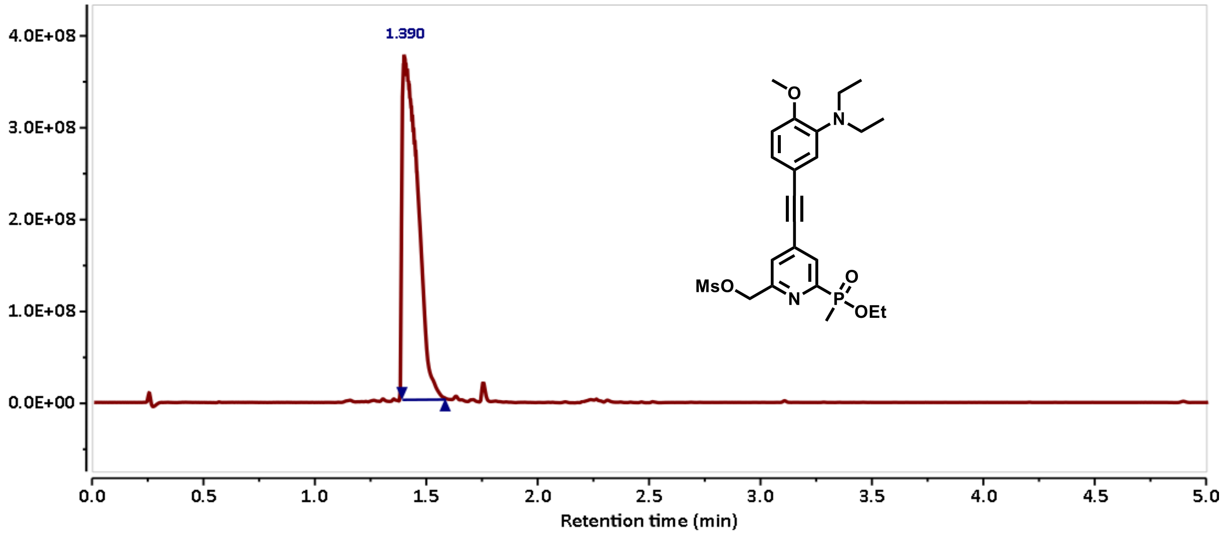

Figure S59. HPLC trace of compound **17**

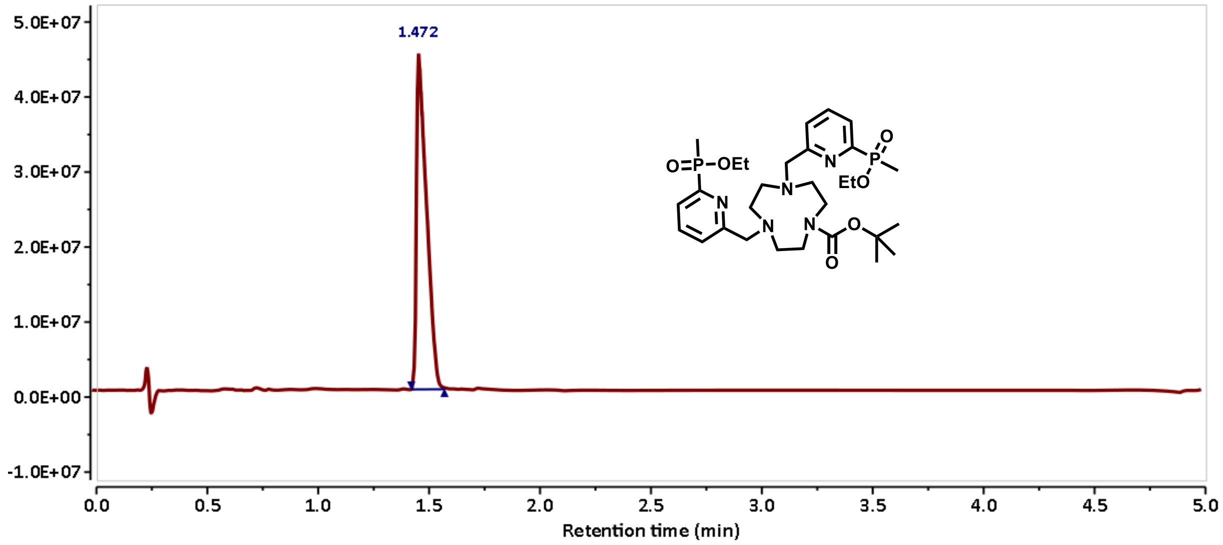

Figure S60. HPLC trace of compound **20**

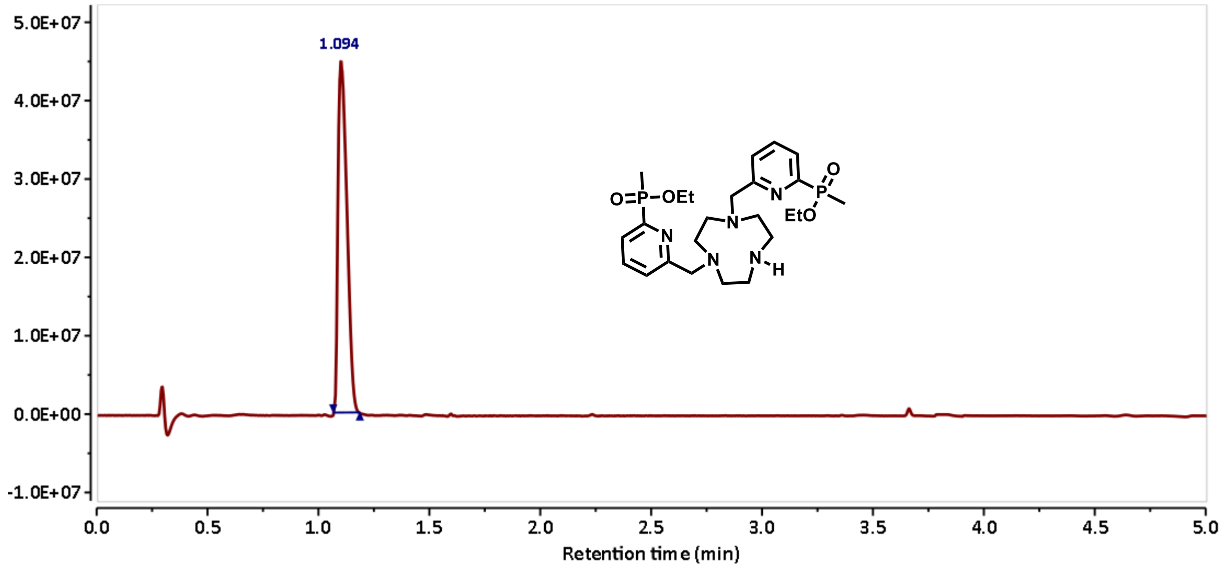

Figure S61. HPLC trace of compound **21**

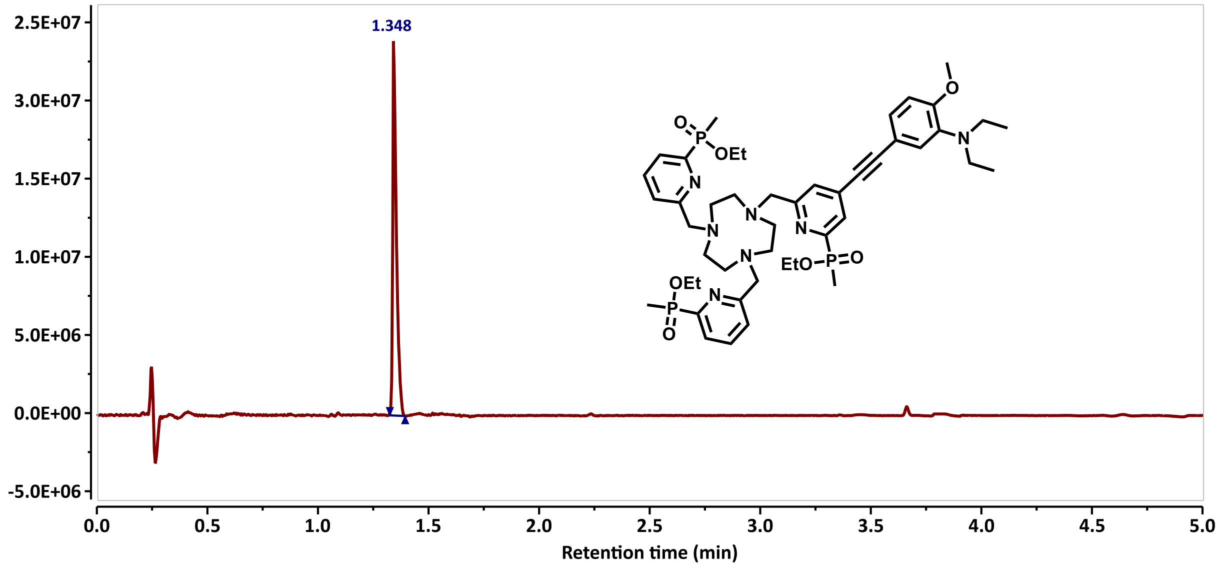

Figure S62. HPLC trace of compound **22**

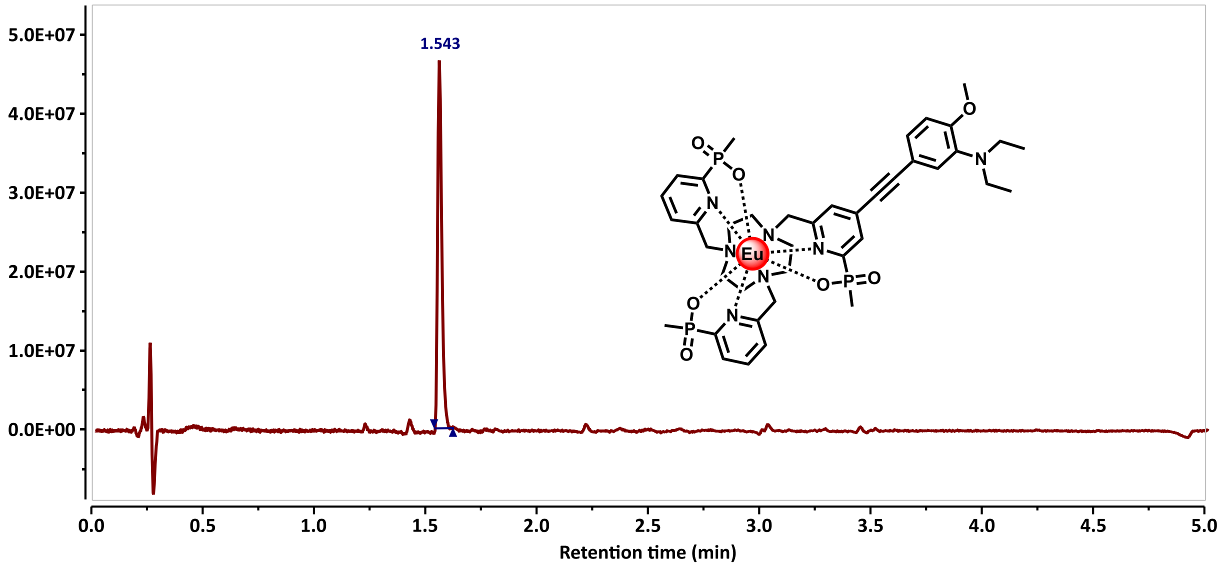

Figure S63. HPLC trace of complex **[Eu*L*[3]]**

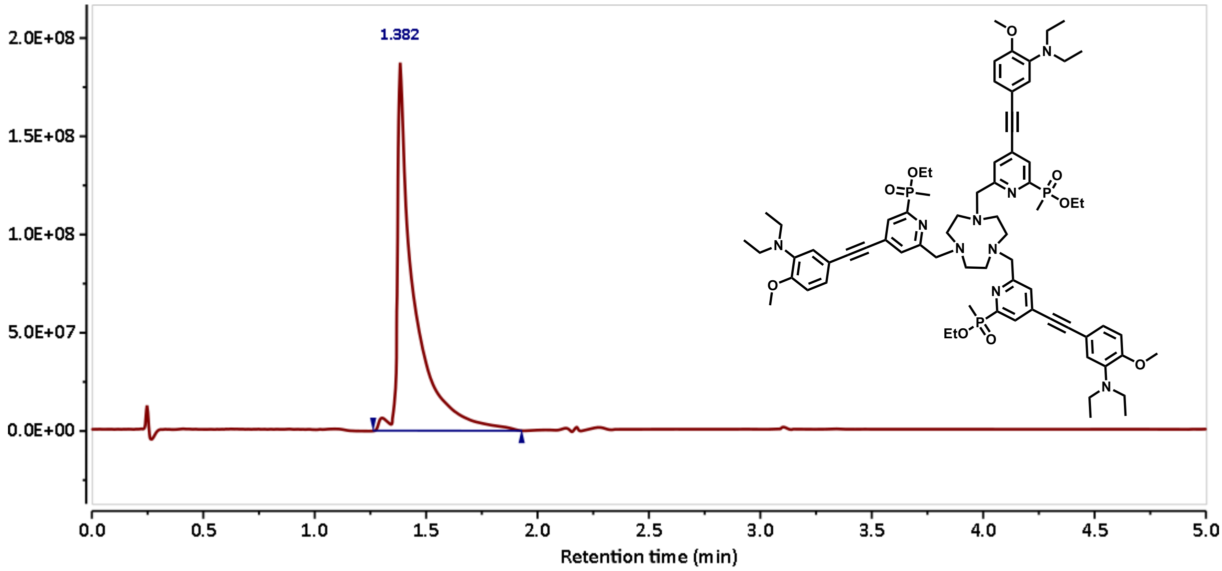

Figure S64. HPLC trace of compound **23**

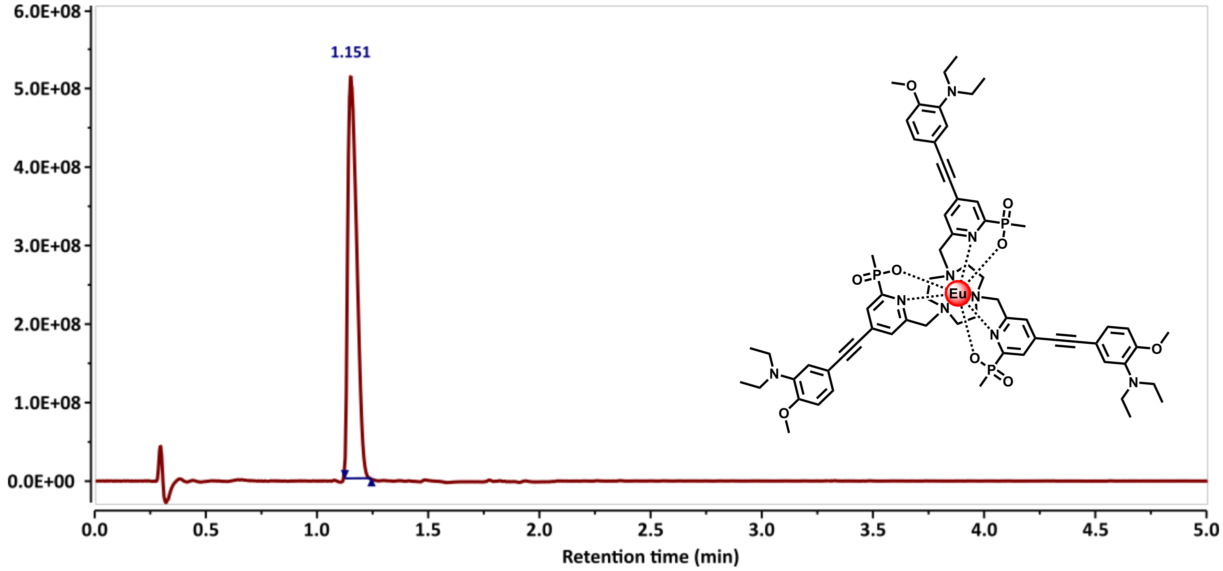

Figure S 65. HPLC trace of complex **[Eu*L*^{4}]**

References

[1] Haas, Y.; Stein, G.; *J. Phys. Chem.,* **1971**, *75*, 3668.

[2] Charbonnière, L. J.; Schurhammer, R.; Mameri, S.; Wipff, G.; Ziessel, R. F.; *Inorg. Chem.,* **2005**, *44*, 7151.

[3] Pal, R.; Beeby, A.; *Methods and Applications in Fluorescence,* **2014**, *2*, 037001.

[4] Pal, R.; *Faraday Discuss.,* **2015**, *177*, 507.
